# Indole‐3‐Propionic Acid Improves Alveolar Development Impairment via Targeting VAMP8‐mediated SNAREs Complex Formation in Bronchopulmonary Dysplasia

**DOI:** 10.1002/advs.202502610

**Published:** 2026-02-06

**Authors:** Beibei Wang, Xu Chen, Haowei Xu, Zhiqi Zeng, Keyu Lu, Yu Mao, Qianru Lv, Hui Shi, Song Liu, Xian Shen, Chunyu Yin, Yang Yang, Yan Guo, Xingyun Wang, Rui Cheng

**Affiliations:** ^1^ Department of Neonatology Children's Hospital of Nanjing Medical University Nanjing China; ^2^ Department of Traditional Chinese Medicine Shanghai Children's Hospital School of Medicine Shanghai Jiao Tong University Shanghai China; ^3^ Department of Pediatrics Suqian Hospital Affiliated to Xuzhou Medical University Suqian China; ^4^ Guangzhou Institute of Pediatrics Guangzhou Women and Children's Medical Center Guangzhou Medical University Guangzhou China

**Keywords:** autophagy, apoptosis, bronchopulmonary dysplasia, indole‐3‐propionic acid, SNARE complex

## Abstract

Bronchopulmonary dysplasia (BPD) disrupts the process of alveolar development, characterized by damage to alveolar epithelial type II cells (AEC II). The present study aims to evaluate the impact of the tryptophan‐derived metabolite indole‐3‐propionic acid (IPA) on postnatal pulmonary development in BPD. Metabolomics indicated that tryptophan metabolic dysfunction is associated with BPD, with IPA emerging as a key metabolite that co‐varies at neonatal levels in both clinical and experimental BPD. Supplementation with IPA protected against hyperoxia‐induced alveolar simplification, which was characterized by increased pro‐proliferative, anti‐apoptotic, and pro‐transdifferentiation activities. Mechanistically, we evaluated circular dichroism (CD), molecular docking, surface plasmon resonance (SPR), and immunoprecipitation techniques, and speculated that IPA exerted its inhibitory effect on phosphorylation of vesicle associated membrane protein 8 (VAMP8) through direct molecular binding. This interaction influenced the assembly of the soluble N‐ethylmaleimide‐sensitive factor attachment protein receptor (SNARE) complex and subsequently promotes autophagosome‐lysosome fusion. In summary, IPA alleviates hyperoxia‐induced alveolar arrest by promoting autophagosome‐lysosome fusion via inhibition of VAMP8 phosphorylation, which is suggestive of a promising therapeutic target of BPD.

## Graphical abstract

This study aims to evaluate the impact of the tryptophan‐derived metabolite indole‐3‐propionic acid (IPA) on lung development and autophagic flux. IPA alleviates hyperoxia‐induced alveolar arrest by promoting autophagosome‐lysosome fusion via inhibition of VAMP8 phosphorylation, which is suggestive of a promising therapeutic target of BPD.

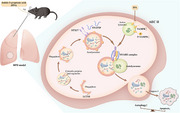

**Abbreviations**
AA: anthranilic acid
AEC II: alveolar epithelial type II cells;
BPD: bronchopulmonary dysplasia
CCK‐8: cell counting kit‐8
CD: circular dichroism
Cdyn: dynamic compliance
CQ: chloroquine
CTL: control
FITC: fluorescein isothiocyanate
HYX: hyperoxic
H&E: hematoxylin and eosin
5‐HIAA: 5‐hydroxyindoleacetic acid
5‐HT: serotonin
IA: indoleacrylic acid
IAN: indole‐3‐acetonitrile
ICA: indole‐3‐carboxaldehyde
ILA: indole‐3‐lactic acid
IPA: indole‐3‐propionic acid
KYN: kynurenine
LC‐MS/MS: liquid chromatography‐tandem mass spectrometry
MLE‐12: murine lung epithelial‐12
MLI: mean linear intercept
MMP: mitochondrial membrane potential
NAS: N‐Acetyl‐5‐hydroxytryptamine
NOX: normoxic
RLairway resistanceSNARE: airway resistanceSNAREsoluble N‐ethylmaleimide‐sensitive factor attachment protein receptor
SPR: Surface plasmon resonance
STX17: syntaxin 17
TEM: Transmission electron microscopy
TRP: Tryptophan
VAMP8: vesicle associated membrane protein 8

## 1 Introduction

Bronchopulmonary dysplasia (BPD) is the most prevalent respiratory complication associated with prematurity. The improved survival among extremely preterm infants, facilitated by antenatal corticosteroids, surfactant therapy, proficient respiratory support, and sophisticated neonatal care techniques, has paradoxically resulted in an increased incidence of BPD‐related morbidity and mortality [1]. At present, there are no definitive treatments for BPD in clinical practice, with current approaches limited to symptomatic management. Therefore, the identification of effective therapeutic targets and strategies is a critical priority for addressing BPD.

Extensive research indicates that hyperoxia‐induced metabolic dysregulation, involving disruptions in amino acid, lipid, and glucose metabolism, significantly contributes to both the onset and progression of BPD [2]. Through metabolic profiling of lung tissue in a mouse model, we observed a reduction in indole‐3‐propionic acid (IPA), a derivative of tryptophan [3, 4], compared to normoxic controls. IPA has been documented to influence mitochondrial function [5] and to contribute to diverse pathophysiological processes such as immune responses, nerve regeneration, and atherosclerosis [6, 7, 8]. Despite its extensive biological roles, no data to data are available about the specific impact of IPA on BPD pathogenesis.

Endogenous autophagy constitutes a critical regulatory mechanism essential for the proper development and morphogenesis of the lung [9]. Impairment of autophagic flux, marked by the accumulation of autophagosomes and disrupted fusion between autophagosomes and lysosomes, can disrupt the equilibrium between cell survival and apoptosis, potentially contributing to BPD development [10]. In this study, we demonstrated that IPA enhances autophagic flux and exhibits pro‐proliferative, anti‐apoptotic, and pro‐transdifferentiation effects in murine lung epithelial‐12 (MLE‐12) cell lines and mouse models of BPD. Furthermore, our findings revealed conformational changes in the soluble N‐ethylmaleimide‐sensitive factor attachment protein receptor (SNARE) components, specifically syntaxin 17 (STX17) localized to autophagosomes and vesicle‐associated membrane protein 8 (VAMP8) localized to lysosomes. Molecular docking combined with surface plasmon resonance (SPR) confirmed high‐affinity binding between IPA and VAMP8. We also observed an increase in VAMP8 phosphorylation under hyperoxic conditions. We hypothesize that IPA's therapeutic efficacy in BPD may be mediated through the promotion of autophagosome‐lysosome fusion via inhibition of VAMP8 phosphorylation. This study underscores the potential of IPA as an innovative therapeutic intervention for BPD, warranting further investigation into its underlying mechanistic pathways and clinical applications.

## 2 Materials and Methods

### 2.1 Clinic Samples Collection

This study enrolled preterm infants born before 32 weeks of gestation who were admitted to the neonatal intensive care unit at Children's Hospital of Nanjing Medical University (Nanjing, China) between January and July 2021. The study protocol was approved by the hospital's ethics committee (Ethics No: 202110094‐1), and written informed consent was obtained from all parents or legal guardians. Participants were divided into two groups: the BPD group and the control (CTL) group. The infants of the BPD group were diagnosed with moderate‐to‐severe bronchopulmonary dysplasia, defined as requiring >21% oxygen for at least 28 days plus supplemental oxygen or positive pressure support at 36 weeks' postmenstrual age [11]. The clinical characteristics of infants are shown in Tables 1 and 2.

**TABLE 1:** Demographic data and clinical variables of infants: for untargeted measurements of metabolites.

Group	CTL(n=21)	BPD(n=19)
Male, n (%)	11 (52.4%)	15 (78.9%)
Gestational age, weeks [M (P25,P75)]$\left(\bar{x}\pm s,\mathrm{weeks}\right)$	30.1 (29.4, 31.3)	28.1 (27.7, 29.6)
Birth weight, g [M (P25, P75)]$\left(\bar{x}\pm s,\;g\right)$	1400 (1175, 1565)	1190 (1020, 1380)
Cesarean section, n (%)	13 (61.9%)	10 (52.6%)
Prenatal hormone, n (%)	8 (38.1%)	7 (36.8%)
1‐min Apgar score ≤ 7, n (%)	2 (9.5%)	6 (31.6%)
5‐min Apgar score ≤ 7, n (%)	0 (0)	4 (21.1%)
NRDS, n (%)	18 (85.7%)	18 (94.7%)
PDA, n (%)	12 (57.1%)	16 (84.2%)
NEC, n (%)	2 (9.5%)	5 (26.3%)
ROP, n (%)	0 (0)	7 (36.8%)

**TABLE 2:** Demographic data and clinical variables of infants: for targeted measurements of tryptophan metabolites.

Group	CTL(n=6)	BPD(n=6)
Male, n (%)	4 (66.7%)	5 (83.3%)
Gestational age, weeks [M (P25, P75)] $\left(\bar{x}\pm s,\mathrm{weeks}\right)$	30.2 (27.9, 31.3)	27.1 (26.4, 29.6)
Birth weight, g [M (P25, P75)]$\left(\bar{x}\pm s,g\right)$	1138 (1068, 1513)	1035 (958, 1150)
Cesarean section, n (%)	4 (66.7%)	3 (50.0%)
Prenatal hormone, n (%)	5 (83.3%)	5 (83.3%)
1‐min Apgar score ≤ 7, n (%)	0 (0)	5 (83.3%)
5‐min Apgar score ≤ 7, n (%)	0 (0)	3 (50.0%)
NRDS, n (%)	6 (100%)	6 (100%)
PDA, n (%)	3 (50.0%)	6 (100%)
NEC, n (%)	1 (16.7%)	1 (16.7%)
ROP, n (%)	2 (33.3%)	3 (50.0%)

### 2.2 Plasma Sample Collection and Metabolites Identification

Peripheral blood samples from participants were collected on the 28th day after birth. After centrifugation at 1250 g for 5 min, the plasma samples were separated and immediately stored at −80°C.

For untargeted measurements of metabolites, a 50 µL aliquot of peripheral blood was mixed with methanol and centrifuged at 13 800g for 15 min at 4°C. Liquid chromatography‐tandem mass spectrometry (LC‐MS/MS) analyses were performed using an UHPLC system (Vanquish, Thermo Fisher Scientific) with a UPLC BEH Amide column (2.1 mm × 100 mm, 1.7 µm) coupled to Q Exactive HFX mass spectrometer (Orbitrap MS, Thermo).

For targeted measurements of tryptophan metabolites, metabolites were extracted and analyzed via a UHPLC system with a Waters HSS T3 column (2.1mm × 150 mm, 2.5 µm) coupled to AB Sciex QTRAP 6500 mass spectrometer.

### 2.3 Animal and Ethics

C57BL/6J mice were acquired from the Animal Core Facility of Nanjing Medical University, Jiangsu, China, and housed under specific pathogen‐free conditions. Through rigorous review, the animal experimental protocol obtained approval from the Animal Care and Use Committee of Nanjing Medical University (Ethics No: IACUC‐2401009).

### 2.4 Murine Model of BPD

Pregnant C57BL/6J mice were individually housed with ad libitum access to food. The environment was upheld under controlled conditions, including a light‐dark cycle of each 12 h, as well as a proper temperature maintained at 23 ± 2°C. Within 12 h after delivery, newborn mice were randomly allocated to either the CTL or BPD group. The CTL group was subjected to ambient room air (21% oxygen), and the BPD group was exposed to 85% oxygen for 14 days. To prevent hyperoxia‐induced toxicity in the nursing dams and ensure consistent nutrition across cohorts, the dams were exchanged between normoxic and hyperoxic conditions every 24 h. Starting from postnatal day 2, the mice were gavaged daily with either 20 mg/kg IPA (Yuanye, B27264) or 50 mg/kg chloroquine (CQ, Selleck, S6999) dissolved in 1.5% DMSO/PBS. For the CTL and BPD groups, 1.5% DMSO/PBS was administered as a vehicle. The supplementation regimen was maintained until postnatal day 14. The mice were then divided into four study groups: CTL, BPD, BPD+IPA, and BPD+IPA+CQ.

### 2.5 Lung Tissue Collection

On the 14th day after birth (P), the experimental animals were euthanized via intraperitoneal injection (i.P.) of pentobarbital sodium at a dosage of 50mg/kg. Following exposure of the thoracic cavity, the lungs were washed with PBS, and then the right main bronchus was ligated. 4% paraformaldehyde (PFA, Servicebio) was used to fix the left lung samples. The right lung samples were frozen to −80 °C for preservation until further use.

### 2.6 Lung Sample Preparation and Metabolomics Analysis

A 50 mg portion of each lung sample was precisely weighed and mixed with methanol. After homogenization at 35 Hz for 4 min, lung samples were then sonicated in an ice‐water bath for 5 min, followed by centrifugation at 13 800 g for 15 min at 4°C. Subsequently, using a centrifugal concentrator, the supernatant was subjected to evaporation until complete dryness was achieved. Eventually, the clear supernatant was subjected to UHPLC‐MS/MS analysis. The UHPLC separation was carried out using an EXIONLC System (Sciex), equipped with a Waters ACQUITY UPLC HSS T3 column (100 × 2.1 mm, 1.8 µm, Waters). The column temperature was set at 40°C. The auto‐sampler temperature was set at 4°C and the injection volume was 5 µL. SCIEX Analyst Work Station Software (Version 1.6.3) and Sciex MultiQuant software (Version 3.0.3) were employed for MRM data acquisition and processing.

### 2.7 Respiratory Function Test

Following anesthesia induced by intraperitoneal injection of pentobarbital sodium, 14‐day‐old mice were secured in a supine position on a surgical platform for tracheal intubation. The mouse was positioned supine with its head and limbs immobilized on the surgical platform. Following depilation of the cervical‐thoracic region, a midline incision was made along the sternum extending toward the neck. The trachea was carefully exposed via blunt dissection, a small proximal incision was created, and a tracheal cannula was inserted and secured with a suture ligature. The animal was then transferred to the platform of the plethysmograph, and the tracheal cannula was connected to the pneumatic circuit of an animal ventilator (RES3050, China). Ventilatory parameters were set as follows: airway pressure 8cm H_2_O, respiratory rate 90 breaths/min, and inspiration‐to‐expiration ratio 20:10. Thoracic excursion and pressure waveforms were monitored to ensure normal ventilation, while airway resistance (RL) and dynamic compliance (Cdyn) were continuously recorded. Physiological data were analyzed using AniRes software.

### 2.8 Lung Histology

Following a series of fixation, dehydration, and embedding procedures, sections with a thickness of 5 µm were prepared and subsequently stained with hematoxylin and eosin (H&E). Except large and small airways, blood vessels, five images from randomly selected fields of each group were examined by light microscopy to assess lung morphometry. The mean linear intercept (MLI) was calculated by superimposing a predetermined grid and randomly positioning lines across the surface. The intersections of these lines with the air‐tissue interface, terminal air space, and secondary septa were examined by artificial counts.

### 2.9 Cell Culture

MLE‐12 cells (CRL‐2110, ATCC) were cultured in DMEM medium (Gibco, C11330500BT) containing 10% FBS (Gibco, 10099‐141) and 1% penicillin/streptomycin (Gibco, 15140122) at 37°C, 5% CO2. The cells were subjected to ambient air (21% oxygen) in the normoxic (NOX) group, while the hyperoxic (HYX) group was exposed to 85% oxygen for 48h. The culture medium was supplemented, as experimental conditions required, with 50 µm IPA (Yuanye, B27264), 10 µm CQ (Selleck, S6999), or 5 µm rapamycin (RAPA, Selleck, S1039).

### 2.10 Western Blotting Analysis

Lung tissues or MLE‐12 cells were lysed in RIPA buffer added with a mixture of protease and phosphatase inhibitors (NCM Biotech, P002) to facilitate the extraction of total protein. A quantity ranging from 20 to 30 µg of proteins, prepared with SDS loading buffer, were segregated through SDS–PAGE using 12.5% gels; transferred onto polyvinylidene fluoride (PVDF) membranes with a pore size of 0.45 µm (Merck millipore, ISEQ00010). PVDF membranes were blocked at room temperature for 2 h using either 5% BSA (Yeasen, 36106ES60) or low‐fat milk powder, followed by incubation with specific antibodies overnight at 4°C: anti‐cleaved caspase‐3 (CST, 9661, 1:1000), anti‐LC3B (Abclonal, A19665, 1:1000), anti‐P62 (Abclonal, A19700, 1:1000), anti‐SFTPC (Proteintech, 10774‐1‐AP, 1:2000), anti‐Rage (Abcam, ab216329, 1:1000), anti‐beta tubulin (Proteintech, 66240‐1‐1g, 1:20000) and anti‐GAPDH (Proteintech, 60004‐1‐1g, 1:50000). Subsequently, horseradish peroxidase (HRP)‐conjugated goat anti‐rabbit IgG (Mulit science, GAR0072, 1:5000) or goat anti‐mouse IgG (H + L) HRP (Mulit science, GAM0072, 1:5000) were incubated with the membranes at room temperature for 2 h. Protein bands were detected with ClarityTM Western ECLSubstrate (Bio‐Rad Laboratories, 170‐5061), and visualized using ChemiDoc XRS+ imaging system (Bio–Rad). Image J software was employed to perform quantitative densitometric analysis.

### 2.11 Immunofluorescence Staining

MLE‐12 cells were fixed with 4% paraformaldehyde and blocked with 5% BSA. Tissue paraffin sections were stained with a TUNEL detection kit (Servicebio, G1505) followed by fluorescent double staining when routine deparaffinization and permeabilization is over. Primary antibodies against RAGE (R&D Systems, MAB1179, 1:200), SFTPC (Proteintech, 10774‐1‐AP, 1:200), Cleaved caspase‐3 (CST, 9661, 1:200), LC3B (Abclonal, A17424, 1:200) were applied at 4 °C overnight, followed by binding to secondary antibody (Invitrogen, A11008; A21422; A21247; 1:500). The sections were counterstained with DAPI after washing with PBS, and observed by using laser scanning confocal microscopy (Leica Stellaris 5, Germany).

### 2.12 Cell Proliferation and Viability Assay

MLE‐12 cells were uniformly plated in 96‐well plates at a concentration of 5 000 cells per well to assess cell proliferation and viability. After cell adhesion, the cells were treated with IPA, indoleacrylic acid (IA), and indole‐3‐lactic acid (ILA) at concentrations of 10, 50, 100, 200, and 500 µm for 48 h. Then, 10 µL of Cell Counting Kit‐8 (CCK‐8) assay solution (Sharebio, SB‐CCK8) was added to each well, followed by incubation for 4 h. The optical density (OD) at 450 nm was measured using a Thermo FC microplate reader (CA, USA).

### 2.13 EdU Assay

The EdU incorporation assay was conducted to evaluate cellular proliferation using the BeyoClick^TM^ EdU‐594 kit (Beyotime, C0078). MLE‐12 cells cultured in confocal dishes were subjected to a 2‐h pulse labeling with EdU solution, followed by fixation with 4% paraformaldehyde and permeabilization with 0.3% Triton X‐100. After incubation with the Azide 594‐labeled Click Reaction Cocktail for 30 min in the dark, the nuclei were counterstained with Hoechst 33342. Imaging was performed using a confocal microscope (Leica Stellaris 5, Germany), capturing multiple random fields for analysis.

### 2.14 Mitochondrial Membrane Potential Detection

Early‐stage cell apoptosis was assessed using a mitochondrial membrane potential assay kit with JC‐1 (Beyotime, C2006). MLE‐12 cells in confocal discs were incubated with JC‐1 staining solution for 20 min at 37°C. The confocal discs were subsequently washed twice with a washing buffer. Next, the monomer and aggregate forms of JC‐1 were visualized using a confocal microscope (Leica Stellaris 5, Germany). Fluorescence density was calculated as the ratio of 596 nm/488 nm fluorescence integrated density by software Image J.

### 2.15 Cell Apoptosis

MLE‐12 cells were seeded at a density of 30,000 cells per well in six‐well plates. Cell culture medium and adherent cells were harvested by centrifugation after intervening for 48 h. Subsequently, in accordance with the Annexin V‐FITC Apoptosis Detection Kit I (Beyotime, C1062L), the cells were resuspended in 195 µL of binding buffer. Under dark conditions, 5 µL of Annexin V‐FITC and 10 µL of propidium iodide were added. The mixture was then incubated at room temperature for 20 min. Ultimately, cellular apoptosis was assessed utilizing the flow cytometer (Becton Dickinson Gallios, USA).

### 2.16 Autophagic Flux Analysis

MLE‐12 cells were infected with pLV‐EF1α‐mCherry‐EGFP‐LC3B adenovirus (Kelsciences) for 12 h. 48 h after transfection, fluorescence density was observed using a Leica Stellaris 5 confocal microscope. Yellow dots (mCherry positive, GFP positive) were visible in the autophagosome, whereas red dots (mCherry positive, GFP negative) indicated an autolysosome, as the green fluorescence was extinguished by acidic lysosomal proteases during the fusion of an autophagosome with a lysosome to form an autolysosome.

### 2.17 Ultrastructural Observation with Transmission Electron Microscopy (TEM)

MLE‐12 cells and lung tissues were fixed with 2.5% glutaraldehyde solution for 30 min at room temperature. After a garose pre‐embedding, the cells were fixed with 1% OsO4 in 0.1 M PB (pH 7.4) for 2 h at room temperature, and then dehydrated in ethanol and acetone gradients. The ultrathin sections were stained with uranyl acetate and lead citrate, and imaged using a H‐7800 TEM (Hitachi, Tokyo, Japan).

### 2.18 Circular Dichrinoism Assay

Circular dichroism (CD) was conducted by Biotech Pack Scientific Company to confirm the secondary structures of VAMP8 (AntibodySystem, YHK73201) and STX17 (FineTest, P5422). The experimental protocol mirrored the methodology reported in existing literature [12].

### 2.19 Molecular Docking of IPA to SNARE Complex

The 3D structure of the Human SNARE complex (PDB ID: 4WY4) was obtained from the RCSB PDB website (https://www.rcsb.org/structure/4WY4) and subsequently processed using Schrödinger's Protein Preparation Wizard, involving hydrogen addition, constrained energy minimization (OPLS2005 force field, 0.30 Å RMSD cutoff), and removal of crystallographic water molecules. Concurrently, the small molecule IPA was converted from 2D to optimized 3D conformation through the LigPrep module with identical force field parameters. Molecular docking simulations were then performed using Schrödinger Maestro 12.8's Glide module in Standard Precision (SP) mode to systematically evaluate binding interactions between the prepared SNARE complex and IPA conformers, generating pose rankings based on GlideScore values.

### 2.20 Surface Plasmon Resonance (SPR)

The SPR technique leverages evanescent field‐induced plasmon resonance at dielectric interfaces to enable label‐free, real‐time quantification of biomolecular interactions on functionalized sensor surfaces, which was used to measure the interaction affinity between compound IPA and recombinant human VAMP8 protein. Briefly, VAMP8 was immobilized on a CM5 sensor chip (BR100530, Cytiva) via amine coupling. Serial dilutions of IPA (0.3125‐10 µm) were injected over the functionalized surface at 30 µL/min. The binding kinetics were evaluated by globally fitting the data to a 1:1 Langmuir interaction model using Biacore Insight Evaluation Software (Cytiva, Marlborough, MA, USA), yielding both association and dissociation rate constants.

### 2.21 Immunoprecipitation

For immunoprecipitation of VAMP8, cells were collected in IP lysis buffer (Thermo Scientific, 87787) supplemented with protease and phosphatase inhibitor cocktails (NCM Biotech, P002). The cellular lysates were subjected with antibody‐conjugated protein A/G magnetic beads (MedChemExpress, HY‐K0202) overnight at 4°C. Then, immunocomplexes were resolved by SDS‐PAGE and analyzed by immunoblot through mouse anti‐Rabbit IgG (Abbkine, A25022). VAMP8 and its phosphorylation levels were analyzed by immunoblot using an antibody against Phospho (Abcam, ab300625, 1:1000) and an anti‐VAMP8 antibody (Abcam, ab76021, 1:10000).

### 2.22 Prediction of Phosphorylation Sites

The VAMP8 protein sequence (UniProt ID: Q9BV40) was retrieved from UniProt and submitted in FASTA format to both NetPhos‐3.1 (DTU Health Tech) and MusiteDeep prediction servers for phosphorylation site analysis.

### 2.23 Statistical Analysis

The data were presented as mean ± SD, and statistical analyses were performed using SPSS Statistics 27. For paired variables, group differences were analyzed by a two‐tailed *t*‐test, while comparison of multiple variables was conducted by using one‐way ANOVA. ^*^ *p* < 0.05, ^**^ *p* < 0.01, ^***^ *p* < 0.001, ^****^ *p* < 0.0001, and NS (no significant).

## 3 Results

### 3.1 IPA is a key Metabolite Downregulated in BPD

To identify specific metabolites associated with BPD, we initially performed liquid chromatography‐tandem mass spectrometry (LC‐MS/MS) on serum samples obtained from neonates diagnosed with or without BPD (Figure S1A). The datasets revealed that the tryptophan metabolic pathway was linked to BPD (Figure S1B). Tryptophan metabolism affects diverse cellular biological functions such as immune response, inflammation, protein synthesis, and oxidative stress, and holds potential as a promising therapeutic target across numerous diseases [13].

**FIGURE 1:**
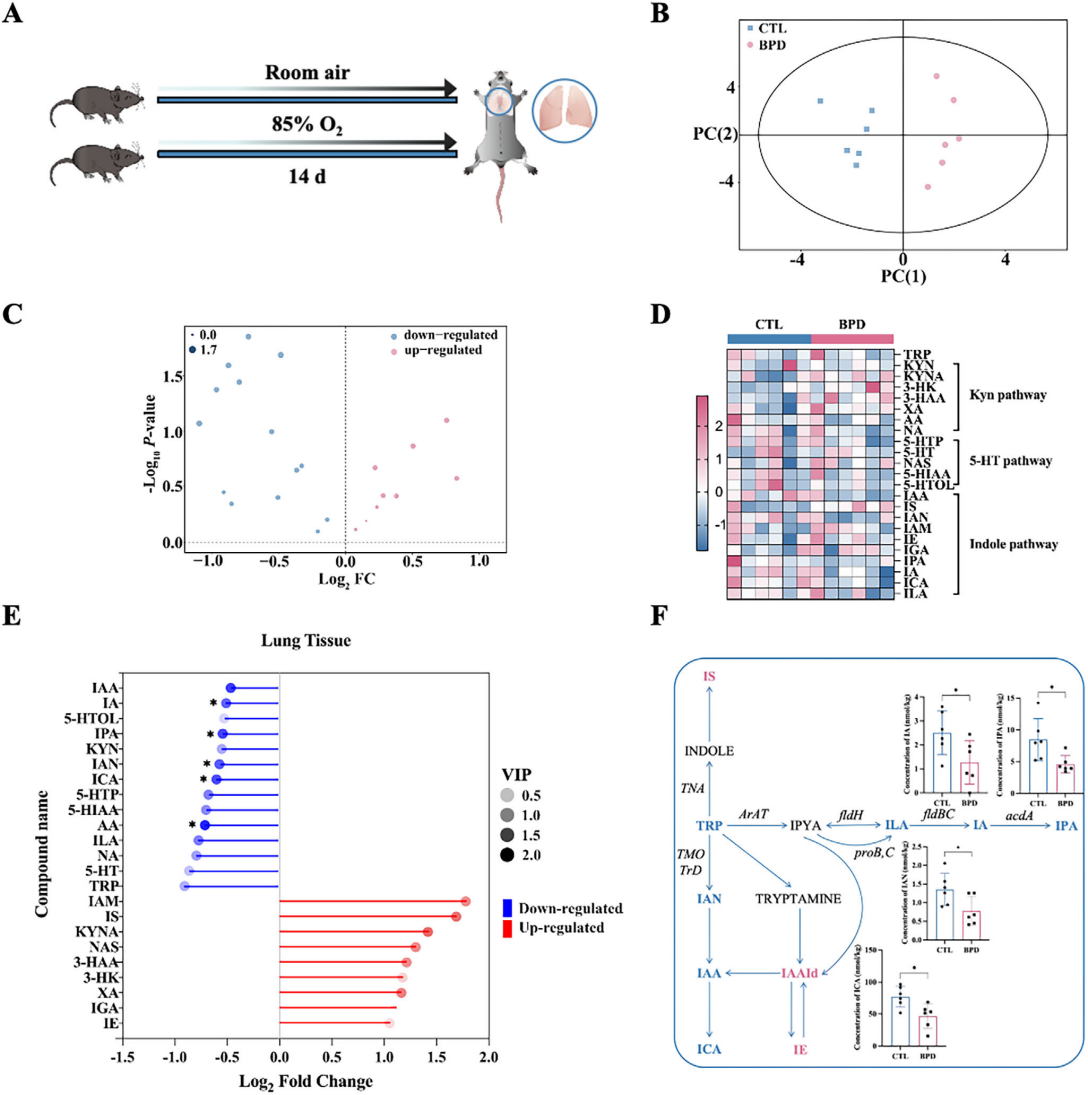
IPA concentration decreases in murine BPD models. (A) Animal model of hyperoxia‐induced BPD. Newborn mice were subjected to either normoxia (ambient air) or hyperoxia (85% O2) exposure with a duration of 14 days. (B) Score plots for the first predictive (PC (1)) and orthogonal (PC (2)) components of the orthogonal partial least squares discriminant analysis (OPLS‐DA) models based on metabolic profiles obtained by LC‐MS/MS (n = 6 biologically independent animals per group). (C) The volcano plot derived from a targeted metabolomic analysis illustrates the metabolites that were increased (red) or decreased (blue) in the BPD group as compared with the CTL group. (D) The heatmap of all detectable tryptophan metabolites. A vectorial version of this figure is provided in Figure S1C. (E) Variations in metabolites within the tryptophan pathway in murine lung tissues. (F) Tryptophan metabolism via the indole pathway and the concentration (nmol/kg) of IAN, ICA, IA, and IPA in the CTL and BPD lung tissues. Data are expressed as mean ± SD, ^*^ *p* < 0.05.

**FIGURE 2:**
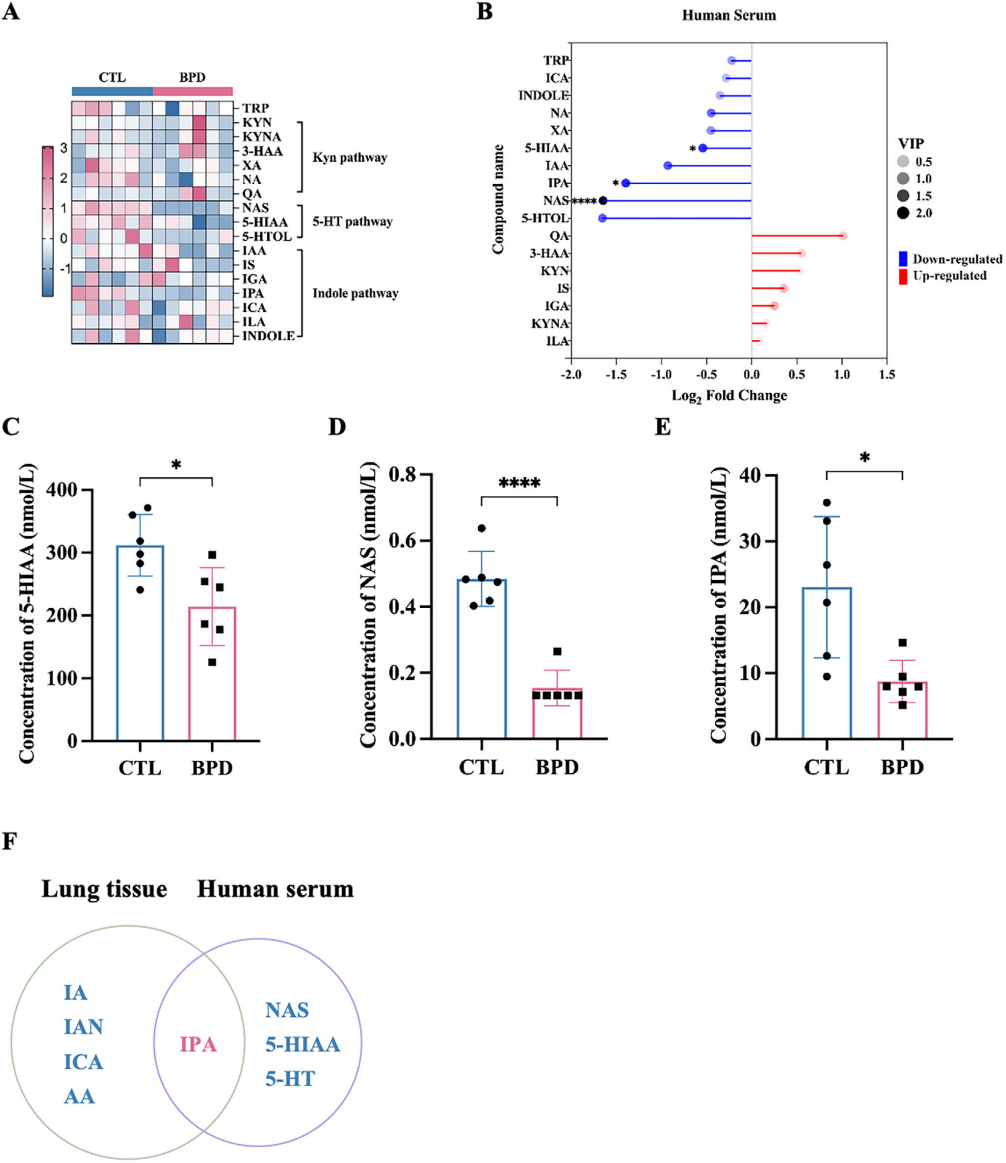
IPA is a key metabolite altered in neonates with BPD. (A) Heatmap of serum tryptophan metabolites. (B) Variations in metabolites within the tryptophan pathway in human serum samples. (C–F) The concentration (nmol/L) of (C) 5‐HIAA, (D)NAS, (E) IPA in human serum samples. (F) Venn diagram of differential metabolites between human serum samples and murine lung tissues. Data are expressed as mean ± SD, ^*^ *p* < 0.05; ^****^ *p* <0.0001.

We subsequently analyzed the differential expression of tryptophan‐related metabolites in lung tissues between the CTL and hyperoxia‐induced BPD murine models using targeted metabolomics (Figure 1A, B). This analysis identified 23 metabolites: 9 were enriched, and 14 were downregulated in BPD (Figure 1C and Figure S1C). Metabolites were classified into kynurenine (Kyn), serotonin (5‐hydroxytryptamine, 5‐HT), and indole pathways (Figure 1D). Notably, significant variations were observed in five tryptophan derivatives: indoleacrylic acid (IA), indole‐3‐acetonitrile (IAN), indole‐3‐carboxaldehyde (ICA), anthranilic acid (AA), and IPA (Figure 1E), with IA, IAN, ICA, and IPA belonging specifically to the indole metabolic pathway (Figure 1F). Consistent with tissue findings, peripheral blood analysis in human BPD infants demonstrated 17 altered tryptophan metabolites (Figure 2A), with significantly decreased levels of 5‐hydroxyindoleacetic acid (5‐HIAA), N‐Acetyl‐5‐hydroxytryptamine (NAS), and IPA compared to controls (Figure 2B–E). IPA exhibited concordant expression patterns across both clinical samples and preclinical animal models (Figure 2F). Together, our findings show that tryptophan metabolism may be implicated in the pathogenesis of BPD, prompting further exploration of IPA's function.

### 3.2 IPA Protects Against Hyperoxia‐Induced Damage

**FIGURE 3:**
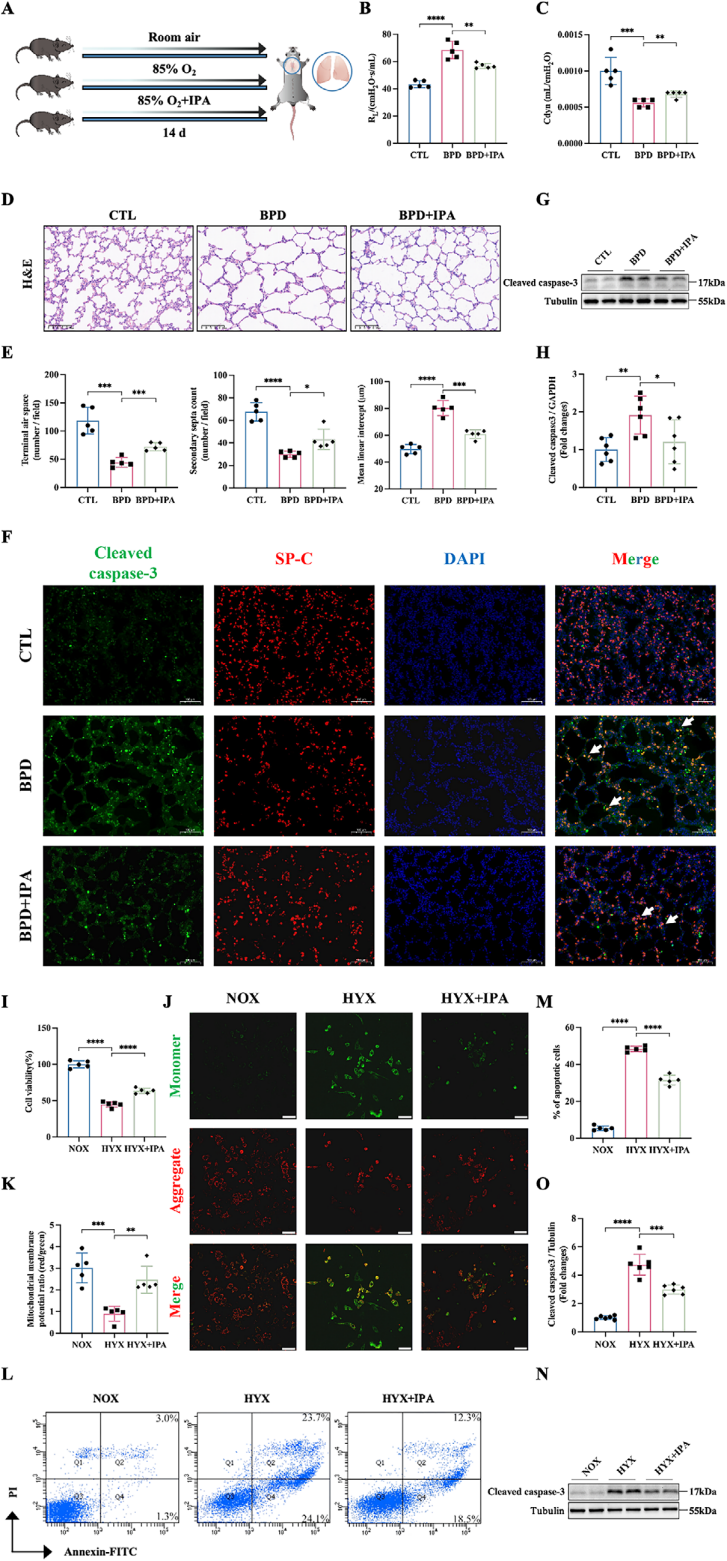
IPA treatment attenuated hyperoxia induced apoptosis. (A) Experimental design schematic. Newborn mice exposed to 85% oxygen were either treated with or without IPA (20mg/kg). (B, C) Pulmonary function tests showing (B) airway resistance (R_L_) and (C) dynamic compliance (Cdyn) in experimental groups (n=5). (D) Representative lung sections stained with H&E. Scale bar: 100 µm. (E) Quantitative morphometric analysis of lung architecture, including terminal airspace, secondary septa, and mean linear intercept (MLI) in the CTL, BPD, and BPD+IPA groups at postnatal day 14 (n=5). (F) Immunofluorescence staining of cleaved caspase‐3 and SP‐C in lung tissues. Scale bar: 100 µm. Arrows indicate the co‐localization (yellow) of cleaved caspase‐3 with SP‐C. (G) Western blot analysis of cleaved caspase‐3 expression in lung tissues. (H) Semi‑quantification of cleaved caspase‐3 expression in (G), normalized to Tubulin (n=6). (I) Cell proliferation assessed by CCK‐8 assay under normoxic (NOX), hyperoxic (HYX), and HYX+IPA conditions. (J) Representative images of mitochondrial membrane potential by JC‐1 staining in experimental groups. Scale bar: 50 µm. (K) Quantitative analysis of JC‐1 fluorescence ratio (red/green) in (J) (n=5). (L) Representative flow cytometry plots of annexin V‐FITC / PI staining for apoptosis detection after 48‐h treatments. FITC, fluorescein isothiocyanate. (M) The percentages of apoptotic cells were quantified by FACS (n=5). (N) Western blot analysis of cleaved caspase‐3 expression in MLE 12 cells. (O) Semi‑quantification of cleaved caspase‐3 expression in (N), normalized to Tubulin (n=6). Data are expressed as mean ± SD, ^*^ *p* < 0.05; ^**^ *p* < 0.01; ^***^ *p* <0.001; ^****^ *p* <0.0001.

To verify the role of IPA, a murine model of BPD induced by postnatal hyperoxia for 14 days was used, and the BPD group was treated with IPA or vehicle via daily oral gavage (Figure 3A). Pulmonary function assessment demonstrated significantly elevated airway resistance (RL) and impaired dynamic compliance (Cdyn) in BPD mice compared to controls. Notably, IPA administration partially restored both respiratory mechanics parameters (Figure 3B, C). Histological analysis showed arrested alveolar development in the BPD group, characterized by enlarged alveoli, diminished terminal airspace, decreased secondary septum, as well as elevated mean linear intercept (MLI). IPA treatment alleviated the pathological changes, significantly increasing alveolarization regions (Figure 3D, E). While the upstream of IPA, such as IA and indole‐3‐lactic acid (ILA), did not improve the pathological changes in the murine BPD model (Figure S2A, B).

Caspase‐dependent apoptosis of AEC II represents a pivotal mechanism in BPD pathogenesis [14]. We postulated that IPA confers anti‐apoptotic protection. Immunofluorescence revealed that IPA administration markedly suppressed hyperoxia‐induced cleaved caspase‐3 overexpression in BPD lung tissues (Figure 3F) and diminished TUNEL‐positive cell prevalence (Figure S2C). Corroborative western blot analysis verified a significant reduction of cleaved caspase‐3 expression (Figure 3G, H). To delineate the dose‐response relationship, mice were subjected to a regimen of escalating IPA concentrations based on the literature [15, 16]. All dosage groups ‐ low (10 mg/kg), medium (20 mg/kg), and high (40 mg/kg) ‐ demonstrated good tolerance to IPA without observable adverse effects. Histopathological analysis revealed substantial improvement in lung pathology at medium and high doses (Figure S2D, E). Western blot analysis confirmed decreased cleaved caspase‐3 expression after medium and high dose IPA treatment (Figure S2F, G).

To confirm IPA's direct effect on hyperoxia‐induced cellular damage, MLE‐12 cells were exposed to hyperoxia (85% oxygen) and treated with a dose titration of IPA, IA, and ILA, followed by CCK‐8 assessment. It was found that only exogenous IPA administration improved cell viability compromised by high oxygen exposure, with an optimal therapeutic concentration of 50 µmol/L (Figure 3I; Figure S3A–C). The proliferative effect of IPA was further evaluated using an EdU assay. It was found that exposure to IPA significantly stimulated the proliferation of MLE‐12 cells, with an efficacy threshold of 50 µmol/L (Figure S3D, E). Depolarization of mitochondrial membrane potential (MMP) is commonly recognized as a critical event marking the initial stages of apoptosis. The JC‐1 probe was utilized to evaluate MMP in the cells. The administration of IPA counteracted the depolarization of MMP observed in the BPD group (Figure 3J, K). As expected, flow cytometry analysis validated that IPA treatment attenuated hyperoxia‐induced apoptosis (Figure 3L, M), with the most pronounced effect observed at 50 µmol/L (Figure S3F, G). Subsequently, a decreased level of cleaved caspase‐3 was also detected by Western blotting in the IPA treatment group (Figure 3N, O).

We next investigated whether IPA facilitates transdifferentiation. Preliminary results revealed that IPA increased the levels of SP‐C, a marker for AEC II, and RAGE, a marker for alveolar epithelial type I cells (AEC I) (Figure S2C), as confirmed by western blot analysis (Figure S4A–C). Critically, immunofluorescence analysis demonstrated a significant increase in cells co‐expressing Rage and SP‐C following IPA treatment (Figure S4D–G). The upregulation of these markers was consistently observed in additional western blot assays (Figure S4H–J). This converging evidence indicates that IPA exerts a pro‐transdifferentiation effect.

Collectively, these results provide strong evidence that IPA mitigates hyperoxia‐induced damage both in vitro and in vivo, underscoring its potential as a therapeutic strategy for BPD.

### 3.3 IPA Enhances Autophagosome‐Lysosome Fusion

**FIGURE 4:**
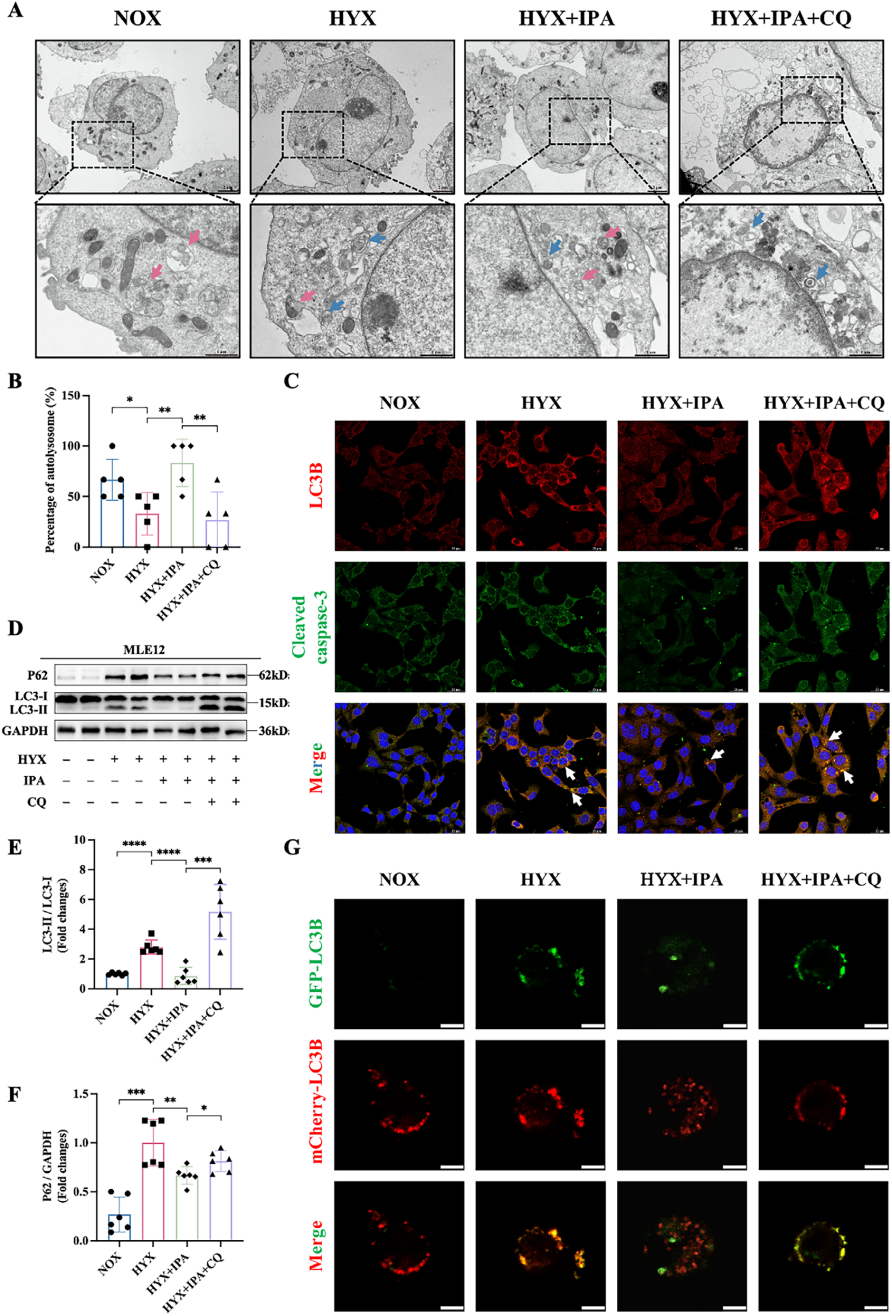
IPA promotes the maturation of autophagosomes into autolysosomes. (A) Transmission electron micrographs of MLE‐12 cells showing autophagic structures (blue arrows: autophagosomes; red arrows: autolysosomes). Scale bars: upper panel = 2 µm; lower panel = 1 µm.(B) Quantitative analysis of autolysosome percentage from TEM images (n=5).(C) Immunofluorescence staining of LC3B (red) and cleaved caspase‐3 (green). Nuclei were counterstained with DAPI (blue). Arrows indicate the co‐localization (yellow) of cleaved caspase‐3 with LC3B. Scale bar: 25 µm.(D) Western blot analysis of LC3‐II/LC3‐I and P62 expression under different treatment conditions.(E‐F) Semi‑quantification of (E) LC3‐II/LC3‐I and (F) P62 expression, normalized to GAPDH (n = 6).(G) GFP‐mCherry‐LC3B assays demonstrating autophagic flux. Yellow puncta represent autophagosomes, while red‐only puncta indicate autolysosomes. Scale bar: 5 µm.Data are expressed as mean ± SD, ^*^ *p* < 0.05; ^**^ *p* < 0.01; ^***^ *p* <0.001, ^****^ *p* <0.0001.

To further elucidate the mechanism of IPA, TEM was employed to examine the ultrastructure of AEC II cells. TEM analysis revealed that hyperoxia increased autophagosomes with a concomitant reduction in autolysosomes, which was reversed by IPA treatment (Figure 4A, B). Autophagy is a critical contributor to the process of cellular apoptosis. Activation of autophagy has been shown to attenuate hyperoxia‐induced lung injury via an anti‐apoptotic mechanism [17]. In our research, as evidenced by Western blot analysis, rapamycin, an autophagy inducer, markedly enhanced autophagic degradation and concurrently reduced apoptosis in hyperoxia‐exposed MLE‐12 cells (Figure S5A–C). Using immunofluorescence staining for LC3B and cleaved caspase‐3, we observed a significant increase in autophagosome accumulation alongside increased apoptotic activity under hyperoxic conditions (Figure 4C). Therefore, autophagic flux was detected by immunoblot assay. As shown by Western blotting, the results of decreased expression of LC3‐II and P62 in hyperoxia‐induced MLE‐12 cells revealed that IPA exhibited an enhancing effect on autophagic activity (Figure 4D–F). Nevertheless, this promotion of autophagy was reversed by chloroquine (CQ) (Figure 4A–F), an inhibitor of late‐stage autophagy [18]. LC3‐II is generated through the conjugation of LC3‐I to phosphatidylethanolamine (PE) molecules. It specifically targets the elongated phagophore and remains on autophagosomes until their subsequent fusion with lysosomes [19]. Therefore, we hypothesized that IPA promotes cargo degradation by facilitating autophagosome‐lysosome fusion.

To test this hypothesis, we used GFP‐mCherry tandemly tagged LC3B to monitor autophagosome‐lysosome fusion. Autophagosomes are labelled by both GFP and mCherry, whereas autolysosomes are marked by mCherry alone due to the quenching of GFP signals in acidic lysosomes. In comparison to the hyperoxia group, GFP^−^ mCherry^+^ LC3B puncta were markedly increased in MLE‐12 cells following IPA intervention (Figure 4G). Thus, IPA promotes the maturation of autophagosomes into autolysosomes.

### 3.4 IPA Promotes Autophagic Flux to Attenuate BPD

**FIGURE 5:**
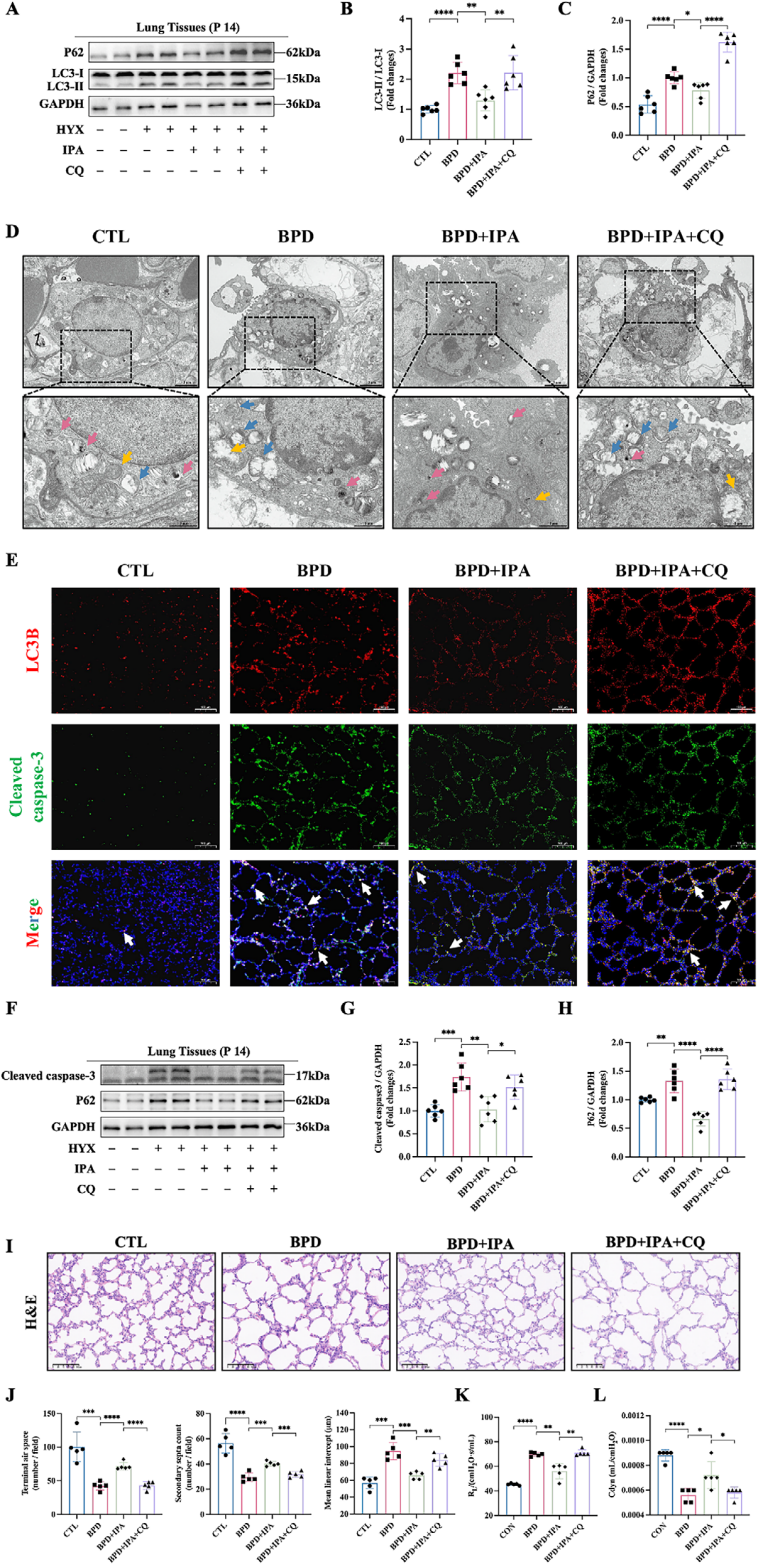
IPA promotes autophagic flux to attenuate BPD. (A) Western blot analysis of LC3‐II/LC3‐I, P62 expression in the CTL, BPD, BPD+IPA and BPD+IPA+CQ groups. (B, C) Quantification of (B) LC3‐II/LC3‐I ratio and (C) P62 expression, normalized to GAPDH (n = 6). (D) Transmission electron micrographs of lung tissues showing autophagic structures (blue arrows: autophagosomes; red arrows: autolysosomes) and mitochondria (yellow arrows). Scale bars: upper panel = 2 µm; lower panel = 1 µm. (E) Immunofluorescence staining of LC3B (red) and cleaved caspase‐3 (green). Nuclei were counterstained with DAPI (blue). Arrows indicate the co‐localization (yellow) of cleaved caspase‐3 with LC3B. Scale bar: 100 µm. (F) Western blot analysis of cleaved caspase‐3 and P62 expression. (G‐H) Semi‐quantification of (G) cleaved caspase‐3 and (H) P62 expression, normalized to GAPDH (n = 6). (I) Representative lung sections stained with H&E. Scale bar: 100 µm. (J) Quantitative morphometric analysis of lung architecture including terminal airspace, secondary septa, and mean linear intercept (MLI) (n=5). (K, L) Pulmonary function tests showing (K) airway resistance (R_L_) and (L) dynamic compliance (Cdyn) (n=5). Data are expressed as mean ± SD, ^*^ *p* < 0.05; ^**^ *p* < 0.01; ^***^ *p* <0.001; ^****^ *p* <0.0001.

To mechanistically interrogate the functional role of autophagic flux in BPD pathogenesis, we pharmacologically inhibited autophagosome‐lysosome fusion through CQ administration in neonatal mice (Figure S6A). No statistically significant difference in body weight and mortality was observed between the BPD+IPA and BPD+IPA+CQ group (Figure S6B, C). Molecular characterization revealed that both BPD and BPD+IPA+CQ groups exhibited accumulated LC3‐II and P62 (Figure 5A–C), indicating autophagic flux impairment. Furthermore, P62 levels demonstrated a dose‐dependent reduction, with significant decreases observed specifically at medium and high doses of IPA (Figure S6D, E). Ultrastructural analysis demonstrated that IPA protects AEC II in BPD by restoring autophagic flux, evidenced by an increased number of autolysosomes and preventing organelle damage, particularly mitochondrial injury. These protective effects were completely blocked by CQ, confirming their dependence on functional autolysosomal degradation (Figure 5D).

Immunofluorescence staining indicated that IPA treatment concurrently reduced LC3B accumulation and suppressed cleaved caspase‐3 expression in BPD lungs (Figure 5E). Western blot analysis further revealed that hyperoxia induced an increase in the autophagy cargo P62, accompanied by cleaved caspase‐3 levels, both of which were reversed by IPA (Figure 5F–H). These findings collectively suggest that IPA modulates apoptosis through its critical influence on autophagy. Importantly, CQ administration not only blocked IPA‐induced autophagic flux but also exacerbated apoptotic signaling (Figure 5E–H) and worsened alveolar simplification (Figure 5I, J) as well as pulmonary function (Figure 5K, L), collectively establishing that IPA ameliorates BPD through autophagy‐dependent modulation of apoptotic pathways.

### 3.5 IPA Targets the SNARE Complex to Promote Autophagic Flow

**FIGURE 6:**
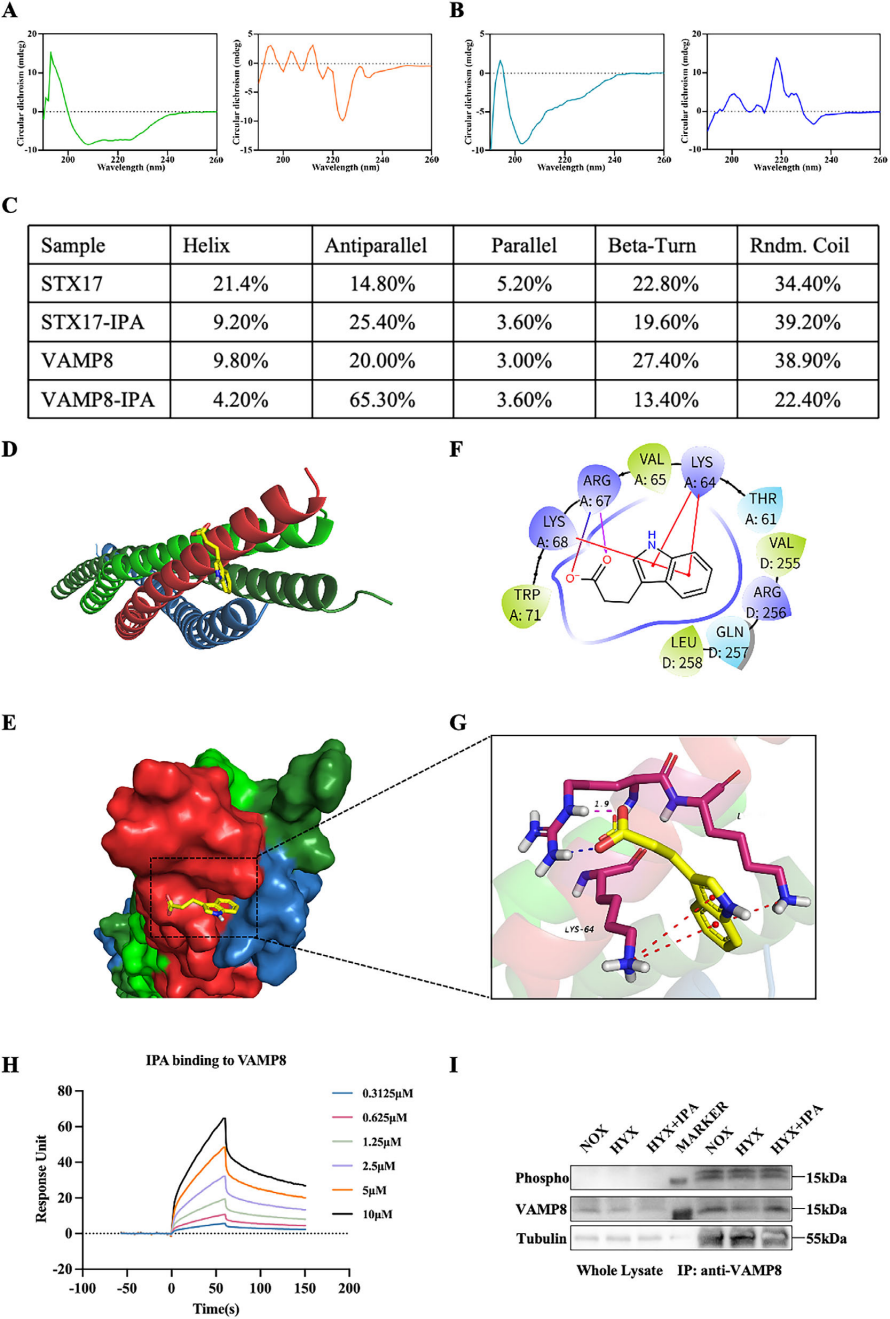
IPA targets SNARE complex to promote autophagic flow. (A, B) Far‐UV circular dichroism spectra (190–260 nm) of (A) STX17 and (B) VAMP8 in the presence or absence of IPA, demonstrating conformational changes. (C) Quantitative secondary structure analysis of STX17 and VAMP8 by CDNN software, showing percentage composition of α‐helix, β‐sheet (parallel/antiparallel), β‐turn, and random coil structures. (D) Molecular docking of IPA (yellow sticks) into the SNARE binding pocket. Protein chains are depicted as cartoon models: VAMP8 (Chain A, red), STX17 (Chain B, green), and SNAP29 (Chains C/D, dark green/blue). (E) Structure‐based protein interaction interface analysis between IPA and SNAREs. (F) 2D diagram of IPA‐SNARE complex interactions with labeled hotspot residues. (G) 3D diagram of IPA‐SNARE complex interactions (atom colors: N = blue, O = red, H = white; IPA = yellow stick). Interactions: salt bridges (blue dashes), cation‐π (red dashes), H‐bonds (purple dashes). (H) Surface plasmon resonance (SPR) sensorgrams quantifying IPA‐VAMP8 binding affinity. (I) Immunoprecipitation analysis of VAMP8 phosphorylation status following IPA treatment.

The SNARE complex, which consists of STX17, SNAP29, and VAMP8, is crucial for the fusion of autophagosomes with lysosomes [20]. To explore the underlying mechanism of IPA‐promoted autophagic flux, we first employed circular dichroism (CD) spectroscopy, a well‐established approach for probing drug‐induced conformational changes in proteins, to assess the secondary structures of STX17 and VAMP8. The CD spectra demonstrated that IPA induced significant structural perturbations in both proteins (Figure 6A–C). We further performed molecular docking of IPA into the SNARE binding pocket. Prediction results from Schrödinger Maestro 12.8 suggested that IPA and the SNARE complex form a protein interaction interface (Figure 6D, E). Molecular docking analysis indicated that IPA directly interacts with Chain A of VAMP8, forming one hydrogen bond, one salt bridge, and three cation‐π interactions. Specifically, the carboxyl oxygen of IPA acts as a hydrogen bond acceptor, engaging with ARG67 at a distance of 1.9 Å. This same carboxyl oxygen also participates in a salt bridge with ARG67. Furthermore, the indole group of IPA establishes three distinct cation‐π interactions with LYS68 and LYS64 (Figure 6F, G). To further validate this interaction, we conducted SPR experiments, which demonstrated that IPA has binding affinity for VAMP8 (Ka = 0.00547 M^−1^ S^−1^, Kd = 0.0278 S^−1^, KD = 5.08 × 10^−6^ M) (Figure 6H).

Phosphorylation of VAMP8 impedes the formation of the STX17‐SNAP29‐VAMP8 complex and inhibits the fusion of autophagosomes and lysosomes [21]. Following this, we screened for the phosphorylation status of autophagic proteins. Based on the molecular mass of the proteins and previous literature [21], we identified a band with pronounced phosphorylation near 15 kD, speculating it to be VAMP8 (Figure S7A). Immunoprecipitation assays demonstrated a significant decrease in VAMP8 phosphorylation levels following IPA treatment, as evidenced by anti‐phospho antibody detection (Figure 6I; Figure S7B).

Using NetPhos‐3.1 and MusiteDeep, we predicted 11 Ser/Thr phosphorylation sites in VAMP8, identifying 8 and 6 high‐confidence sites (score ≥0.5), respectively (Figure S7C, D). The predictions showed strong concordance, with sites clustering in two functional regions: (1) N‐terminal Ser5/Ser18 potentially regulating protein folding/localization, and (2) a central helical segment (Thr28‐Ser55) likely mediating conformational changes and protein interactions through multi‐site phosphorylation (Figure S7E). Notably, these sites exhibit a stepwise helical distribution, suggesting stage‐dependent phosphorylation regulation. Molecular docking revealed IPA binding to this central phosphorylation region (Figure S7F), supporting its potential phosphorylation‐inhibitory mechanism.

Collectively, our main results show that IPA exerted its inhibitory effect on VAMP8 phosphorylation through direct molecular binding. This interaction may influence the assembly of the SNARE complex and subsequently promote autophagosome‐lysosome fusion.

## 4 Discussion

BPD is a common respiratory dysfunction related to prolonged oxygen inhalation in preterm infants, leading to long‐term complications for the respiratory and nervous systems. Recent research has demonstrated that aberrant metabolism of glucose, lipids, and amino acids contributes to the pathogenesis and progression of BPD [22]. By untargeted metabolomics, we found that tryptophan metabolism was dysregulated in infants with developed BPD. Further validation using clinical peripheral blood samples and a hyperoxia‐induced murine BPD model confirmed that hyperoxia is associated with tryptophan metabolic dysfunction and decreased IPA levels in both peripheral circulation and murine lung tissues. IPA supplementation protected against alveolar simplification during BPD progression and therapeutic management of BPD phenotypes. Mechanistically, we elucidated the regulatory mechanism of tryptophan‐derived metabolite IPA through SNARE complex‐mediated autophagosome‐lysosome fusion to alleviate hyperoxia‐induced BPD.

Tryptophan (Trp) is a vital amino acid necessary for protein synthesis and acts as a precursor for the synthesis of multiple important bioactive metabolites. There are three principal pathways of Trp metabolism: the Kyn pathway, the 5‐HT pathway, and the indole pathway. The Kyn pathway is implicated in inflammation, immune responses, and excitatory neurotransmission [3]. 3‐Hydroxyanthranilic acid (3‐HAA), a Kyn pathway metabolite, was significantly decreased in tracheal aspirates from BPD infants. Nebulized 3‐HAA administration enhanced alveolarization and attenuated hyperoxia‐induced AEC II injury in experimental BPD models [23]. The 5‐HT pathway synthesizes 5‐HT, a pivotal neurotransmitter, growth factor, and hormone mediating diverse physiological functions [24]. Indole derivatives, including compounds such as ILA, IAA, and IPA, are involved in intestinal permeability, immune response, and the regulation of inflammation [4]. Alterations in Trp metabolism are observed in various metabolic, neurological, gastrointestinal, and psychiatric disorders, highlighting its potential as a therapeutic target [25]. Herein, our findings indicate that dysregulated Trp metabolism functions as a negative regulatory mechanism affecting lung development.

BPD may result in the dysregulation of amino acid metabolism, encompassing irregularities in amino acid metabolic pathways, metabolites, or essential metabolic enzymes [2]. Metabolomics analyses have revealed disrupted amino acid metabolism in the blood, tracheal aspirates, and amniotic fluid of preterm infants diagnosed with BPD [26, 27, 28]. Animal studies have demonstrated that hyperoxic exposure leads to a reduction in plasma levels of L‐citrulline and L‐arginine in newborn rats [29]. Disordered amino acid metabolism significantly influences the regulation of cellular proliferation, apoptosis, differentiation, senescence, and autophagy [30]. In this study, we found an inverse correlation between IPA and BPD development. More specifically, IPA has been shown to confer protection against diastolic dysfunction [31], mitigate radiotoxicity [32], modulate cancer immunotherapy [6], and extend posttraumatic survival time [33]. However, its impact on improving alveolar simplification has not been reported. We discovered that IPA exhibited pro‐proliferative, anti‐apoptotic, and pro‐transdifferentiation effects via activating autophagic flow in relation to hyperoxia induced BPD.

Autophagy serves as a crucial regulatory mechanism that is present at basal levels throughout lung development, potentially contributing to the phenotype of BPD when its function is compromised [9]. Autophagic flux refers to the complete sequence of autophagic events occurring over a duration, which encompasses multiple stages: initiation of autophagy, autophagosome formation, autophagosome‐lysosome fusion, and subsequent degradation within autolysosomes [19, 34]. Despite the established role of hyperoxia in activating autophagy, as evidenced by significant upregulation of multiple autophagic markers (e.g., ATG5, BECN1, and LC3‐II) in both cellular and animal models, its effects on late‐stage autophagy remain insufficiently investigated [17]. Hyperoxia‐induced blockade of autophagic flux, characterized by increased LC3‐II and P62 expression, may be a key factor in BPD pathogenesis, leading to increased pulmonary apoptosis and abnormal alveolar development [35, 36]. Recent researches have shown that arrest of lung development in BPD could be rescued by regulating autophagic flow [37, 38]. Our study reveals that IPA protects against hyperoxia‐induced apoptosis by preserving autophagic flux in BPD mice and alveolar epithelial type II cells.

This study confirmed that hyperoxia‐induced BPD inhibits the fusion of autophagosomes with lysosomes, blocking autophagic flux, consistent with previous research [35, 36]. The SNARE complex, comprising STX17, SNAP29, and VAMP8, is essential for autophagosome‐lysosome fusion [39]. The assembly of the complex is governed by post‐transcriptional modifications that modify essential ionic charges necessary for protein interactions or introduce steric hindrance within the SNARE complex [39, 40, 41]. Phosphorylation of VAMP8 has been reported to disrupt the assembly of the STX17‐SNAP29‐VAMP8 complex, ultimately resulting in impaired autophagosome‐lysosome fusion [21]. The presented results in our study suggest that IPA‐mediated inhibition of VAMP8 phosphorylation promotes autophagosome‐lysosome fusion. Indeed, our study provides the first evidence that interfering with SNARE complex formation may affect apoptotic cell death in BPD.

## 5 Conclusion

Taken together, we found that IPA was inversely correlated with lung development in preclinical animal models for BPD. Exogenous administration of IPA rescued hyperoxia‐induced autophagic flux blockage and apoptosis in BPD. This protective role of IPA was mediated, in part, by its inhibitory effect on VAMP8 phosphorylation to stabilize the SNARE complex. Our results suggest that tryptophan‐derived metabolites could facilitate the development of preventive and therapeutic strategies against BPD.

## Author Contributions

R. C., X. W., and C. Y. designed the research. B. W., X. C., K. L., Y. M., Q. L., H. S., S. L., X. S., Y. Y., and Y. G. performed the experiments. B. W. analyzed the data and wrote the manuscript. R. C. and X. W. provided valuable comments and revised the manuscript. All the authors read and approved the final version of the manuscript.

## Funding

This work was supported by the National Natural Science Foundation of China [Grant No. 82171705 to Rui Cheng, No. 82371721 and No. 82571982 to Xingyun Wang]; Jiangsu Province Association of Maternal and Child Health (Grant No. FYX202327 to Rui Cheng); Shanghai Rising‐Star Program (Grant No. 22QB1401000 to Xingyun Wang).

## Conflicts of Interest

The author declares no conflicts of interest.

## Ethics Approval and Consent to Participate

This clinic sample collection was approved by the hospital's ethics committee (Approval No. 202110094‐1), and written informed consent was obtained from all parents or legal guardians. We declare that we have received the written informed consent from participants in this study. The animal studies protocols were approved by the Animal Care and Use Committee of Nanjing Medical University with Protocol numbers: IACUC‐2401009.

## Consent for Publication

Data/manuscript publication was approved by all authors. This manuscript does not contain data from any individual person.

## Supporting information




**Supporting File**: advs74104‐sup‐0001‐SuppMat.docx.

**Supporting File**: advs74104‐supp‐0002‐DataFile.

## Data Availability

The data supporting the findings of this study are available from the corresponding author upon reasonable request.
