# Supplementary material for: Indole‐3‐Propionic Acid Improves Alveolar Development Impairment via Targeting VAMP8‐mediated SNAREs Complex Formation in Bronchopulmonary Dysplasia
Source: Adv Sci (Weinh). 2026 Feb 6;13(19):e02610. doi: 10.1002/advs.202502610 (PMC13045412; doi:10.1002/advs.202502610)
Supplement: Supplementary file 1 — Supporting File: advs74104‐sup‐0001‐SuppMat.docx. [file ADVS-13-e02610-s002.docx]

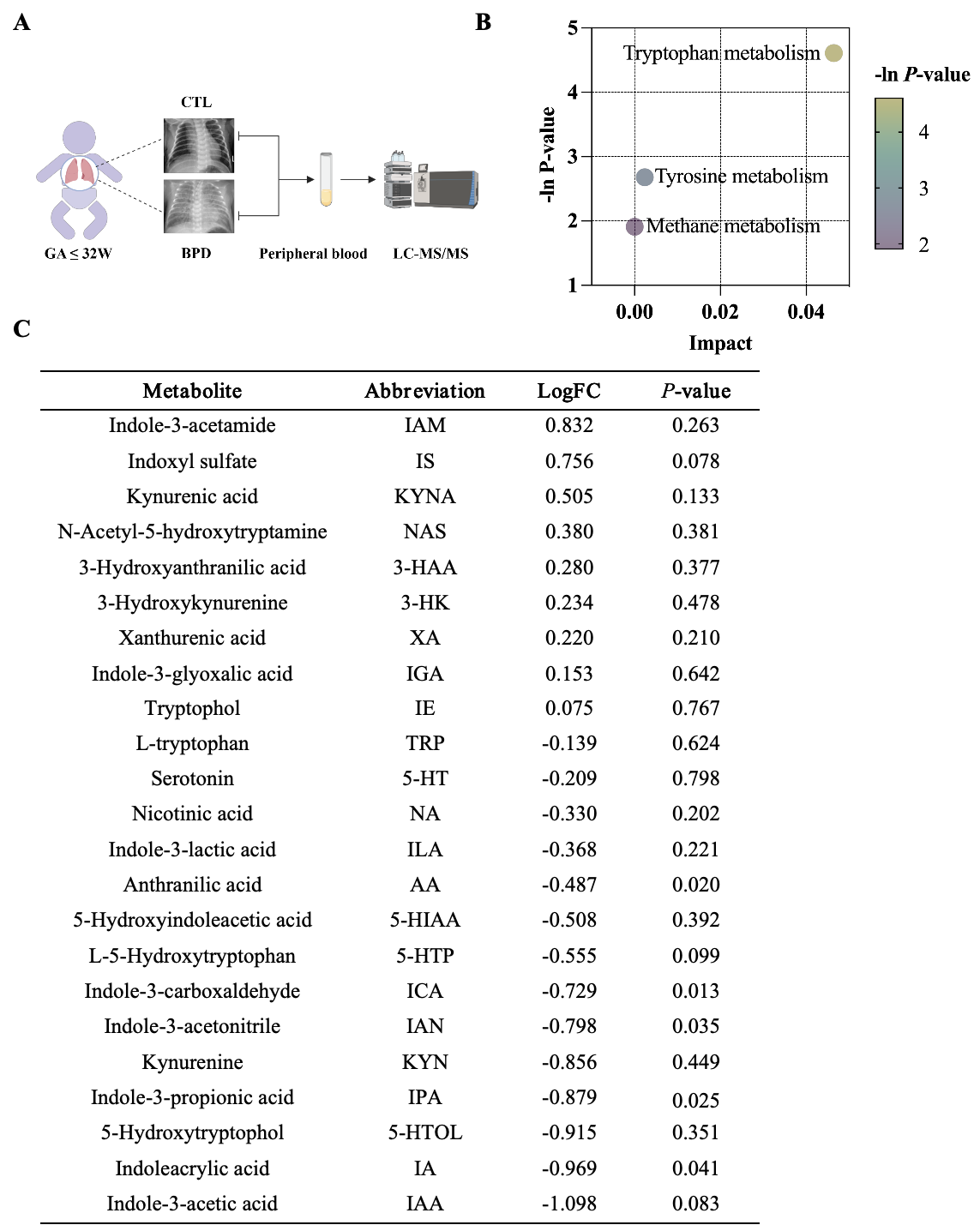


**Figure. S1**

1. Study design and sample collection. Premature infants born before 32 weeks of gestation were enrolled from the neonatal intensive care unit at Children’s Hospital of Nanjing Medical University between January 2021 and July 2021. Peripheral blood samples were collected at 28 days after birth from both the CTL and BPD groups.
2. KEGG pathway analysis of differentially expressed metabolites.
3. Table displays upregulated and downregulated metabolites in lung tissues from the CTL and hyperoxia-induced BPD murine models. Data represent logarithmic fold changes (logFC) and corresponding *P*-values (n = 6).


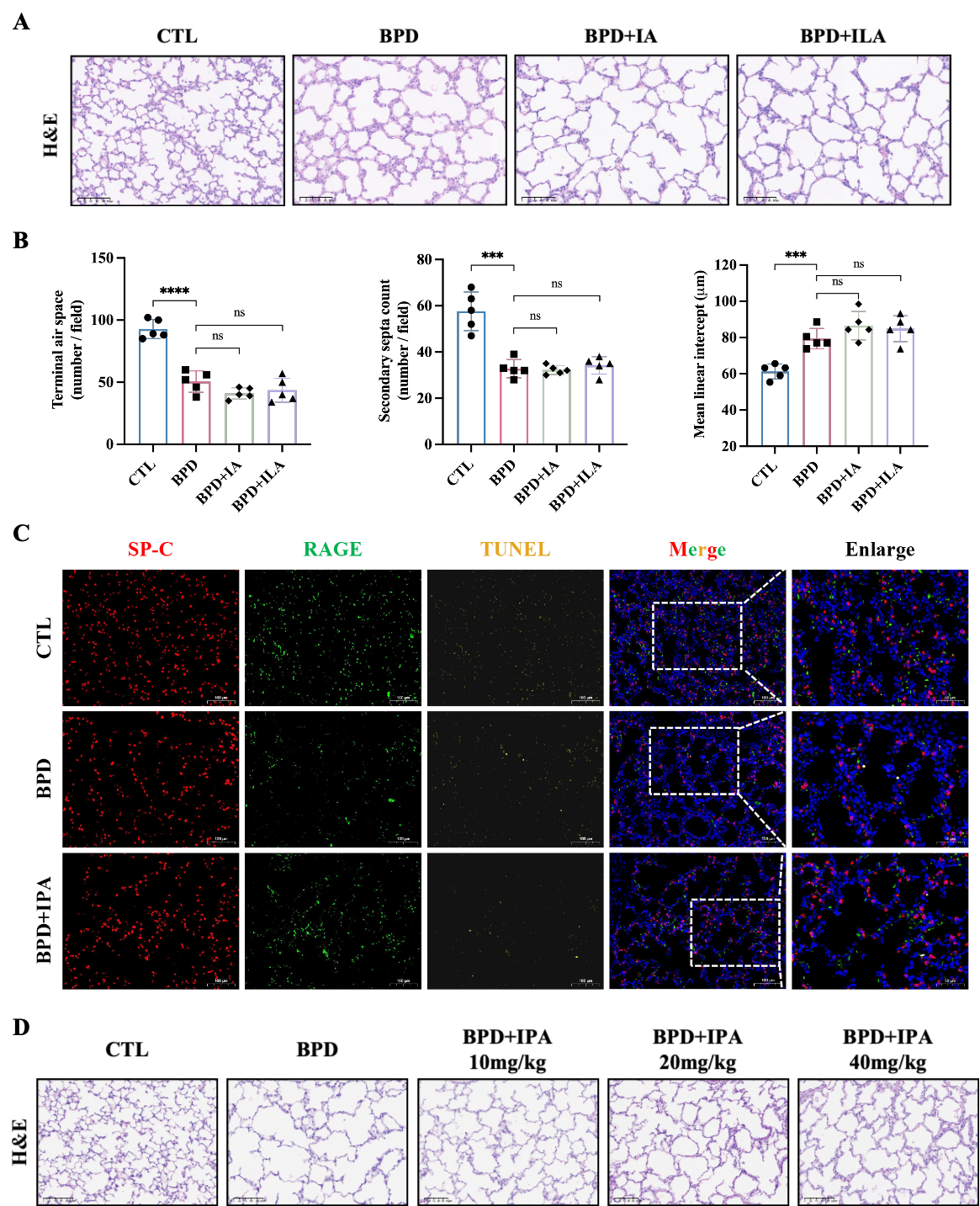

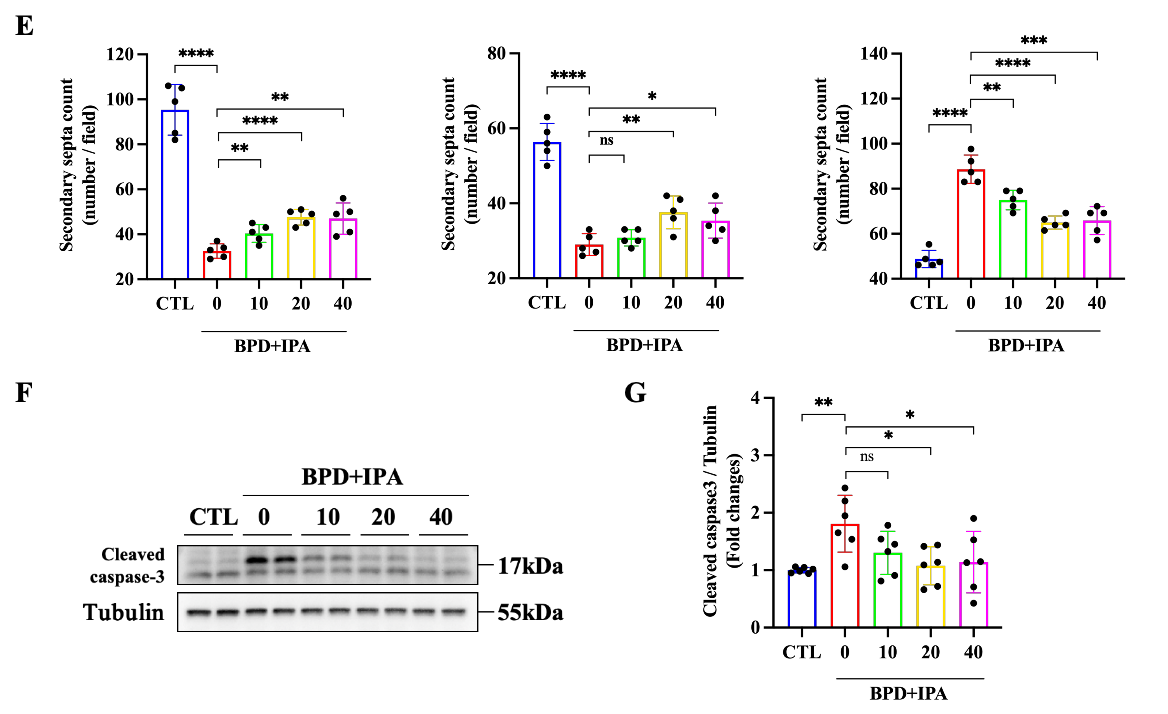


**Figure. S2**

1. Representative lung sections stained with H&E. Scale bar: 100 μm.
2. Quantitative morphometric analysis of lung architecture including terminal airspace, secondary septa, and mean linear intercept (MLI) in the CTL, BPD, BPD+IA, BPD+ILA groups at postnatal day 14 (n=5).
3. Immunofluorescence staining of TUNEL, SP-C and RAGE in lung tissues. Scale bar: 100 μm.
4. Representative lung histomorphology following a regimen of escalating IPA concentrations. Scale bar: 100 μm.
5. Quantitative morphometric analysis of lung architecture including terminal airspace, secondary septa, and mean linear intercept (MLI).
6. Western blot analysis of cleaved caspase-3 expression in lung tissues after treatment with increasing doses of IPA (10, 20, and 40 mg/kg).
7. Semi‑quantification of cleaved caspase-3 expression in (F) , normalized to Tubulin (n=6).

Data are expressed as mean ± SD, **p* < 0.05; ***p* < 0.01; ****p* <0.001; *****p* <0.0001.


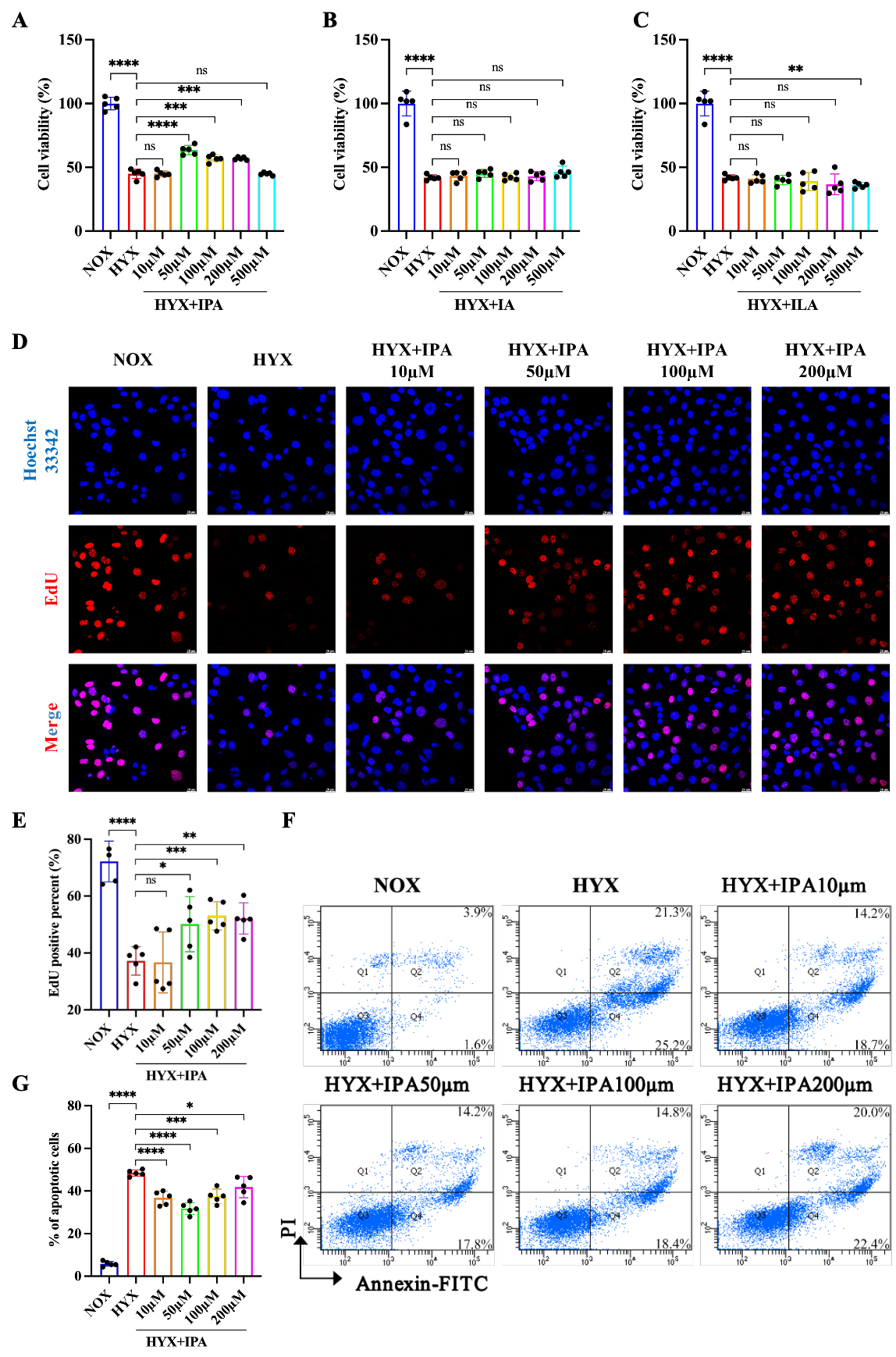


**Figure. S3**

**(**A-C) Cell viability of MLE-12 cells incubated with varying concentrations of (A) IPA, (B) IA, (C) ILA assessed by CCK-8 assay.

(D) EdU Assay under different IPA concentrations.

(E) Quantification of EdU-positive cells.

(F) Representative flow cytometry plots of annexin V-FITC / PI staining for apoptosis detection after 48-hour treatment with different concentrations.

(G) The percentages of apoptotic cells were quantified by FACS (n=5).

Data are expressed as mean ± SD, **p* < 0.05; ***p* < 0.01; ****p* <0.001; *****p* <0.0001.


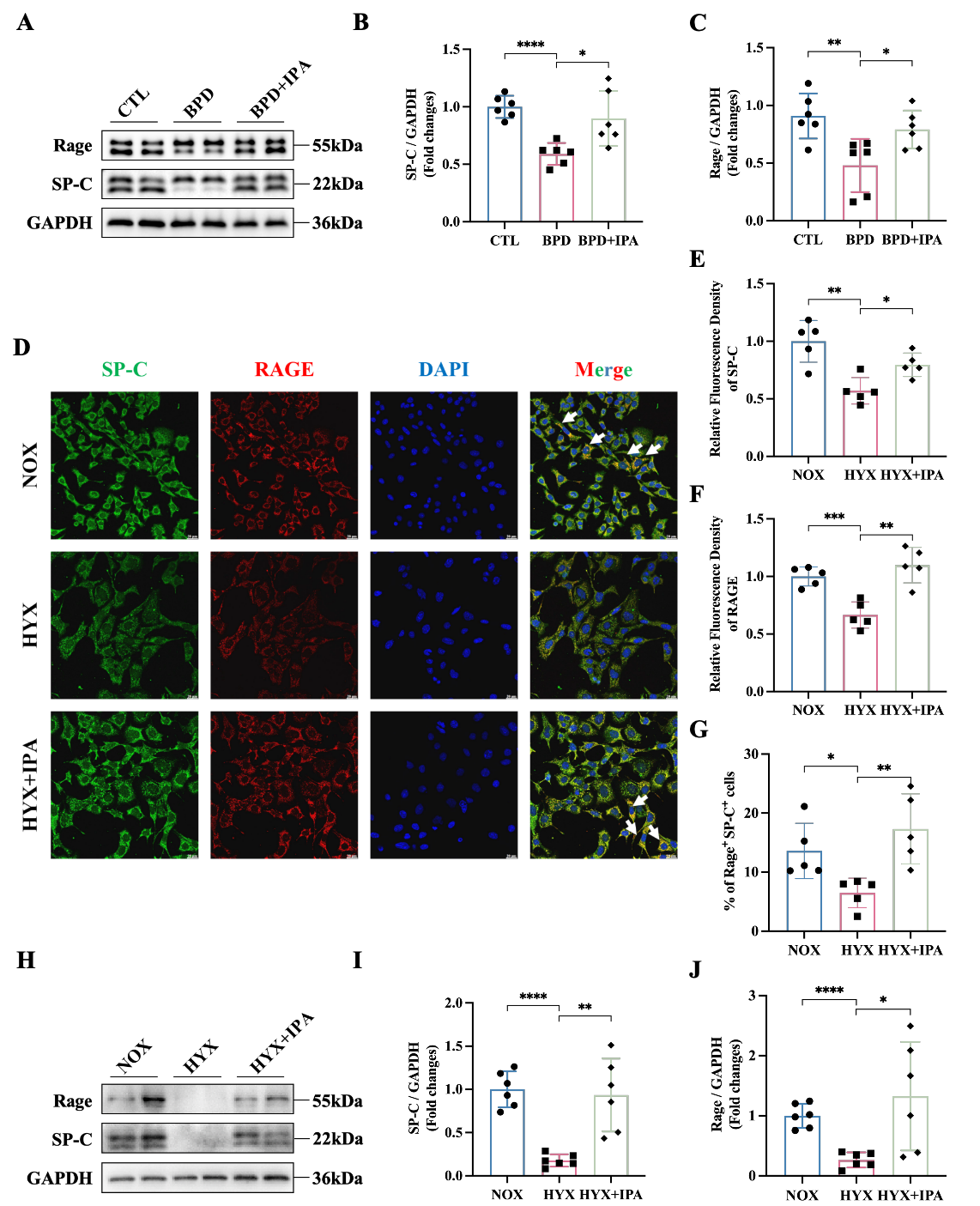


**Figure. S4**

(A) Western blot analysis of SP-C and Rage expression in lung tissues.

(B-C) Semi‑quantification of (B) SP-C and (C) Rage expression, normalized to GAPDH (n = 6).

(D) Immunofluorescence staining of SP-C and RAGE in MLE-12 cells. Arrows indicate the co-localization (yellow) of Rage with SP-C. Scale bar: 20 μm.

(E-F) Quantitative fluorescence intensity analysis of (E) SP-C and (F) RAGE in (D) (n=5).

(G) The percentage of Rage^+^ SP-C^+^ cells in MLE-12 cells.

(H) Western blot analysis of SP-C and Rage expression in MLE-12 cells..

(I-J) Semi‑quantification of (I) SP-C and (J) Rage expression, normalized to GAPDH (n = 6).

Data are expressed as mean ± SD, **p* < 0.05; ***p* < 0.01; ****p* <0.001; *****p* <0.0001.


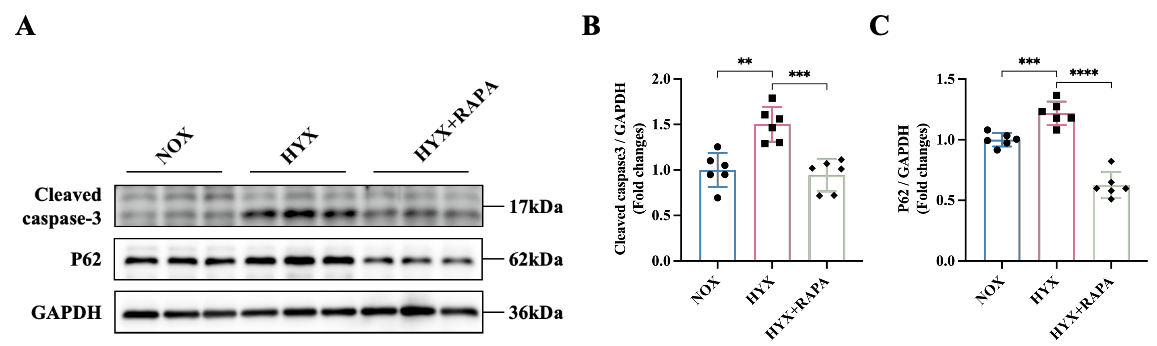


**Figure. S5**

1. Western blot analysis of cleaved caspase-3 and P62 expression in the NOX, HYX, and HYX+RAPA groups.

(B-C) Semi-quantification of (B) cleaved caspase-3 and (C) P62 expression, normalized to GAPDH (n = 6).

Data are expressed as mean ± SD, ***p* < 0.01; ****p* <0.001; *****p* <0.0001.


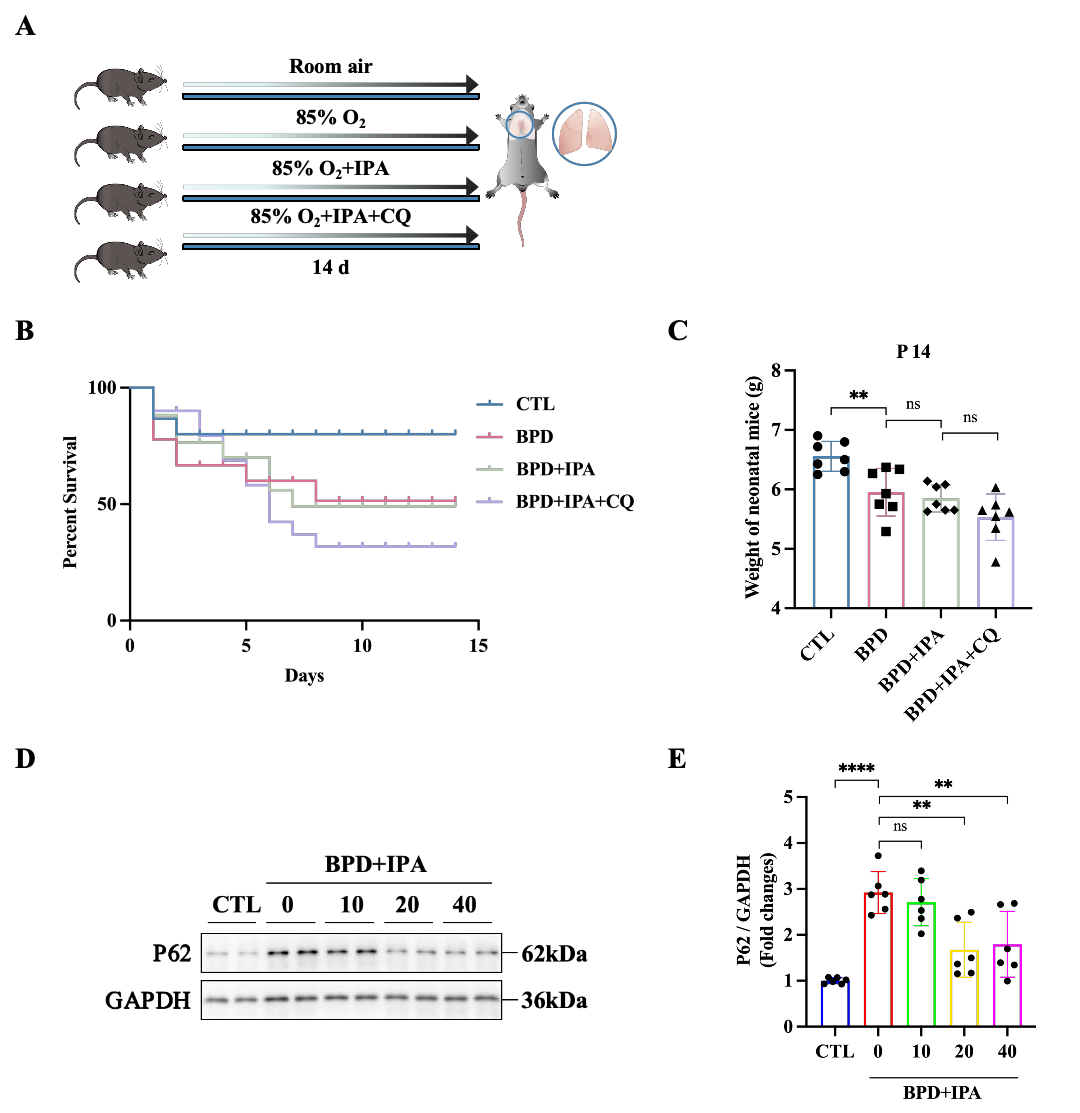


**Figure. S6**

1. Experimental design schematic. Newborn mice exposed to 85% oxygen with daily gavage of vehicle, IPA (20mg/kg) with or without CQ (50mg/kg) until 14 days of age.
2. Survival analysis across experimental groups.
3. Evaluation of body weights across experimental groups.
4. Western blot analysis of P62 expression in lung tissues after treatment with increasing doses of IPA (10, 20, and 40 mg/kg).
5. Semi-quantification of P62 expression in (D), normalized to GAPDH (n = 6).

Data are expressed as mean ± SD, ***p* < 0.01; *****p* <0.0001.


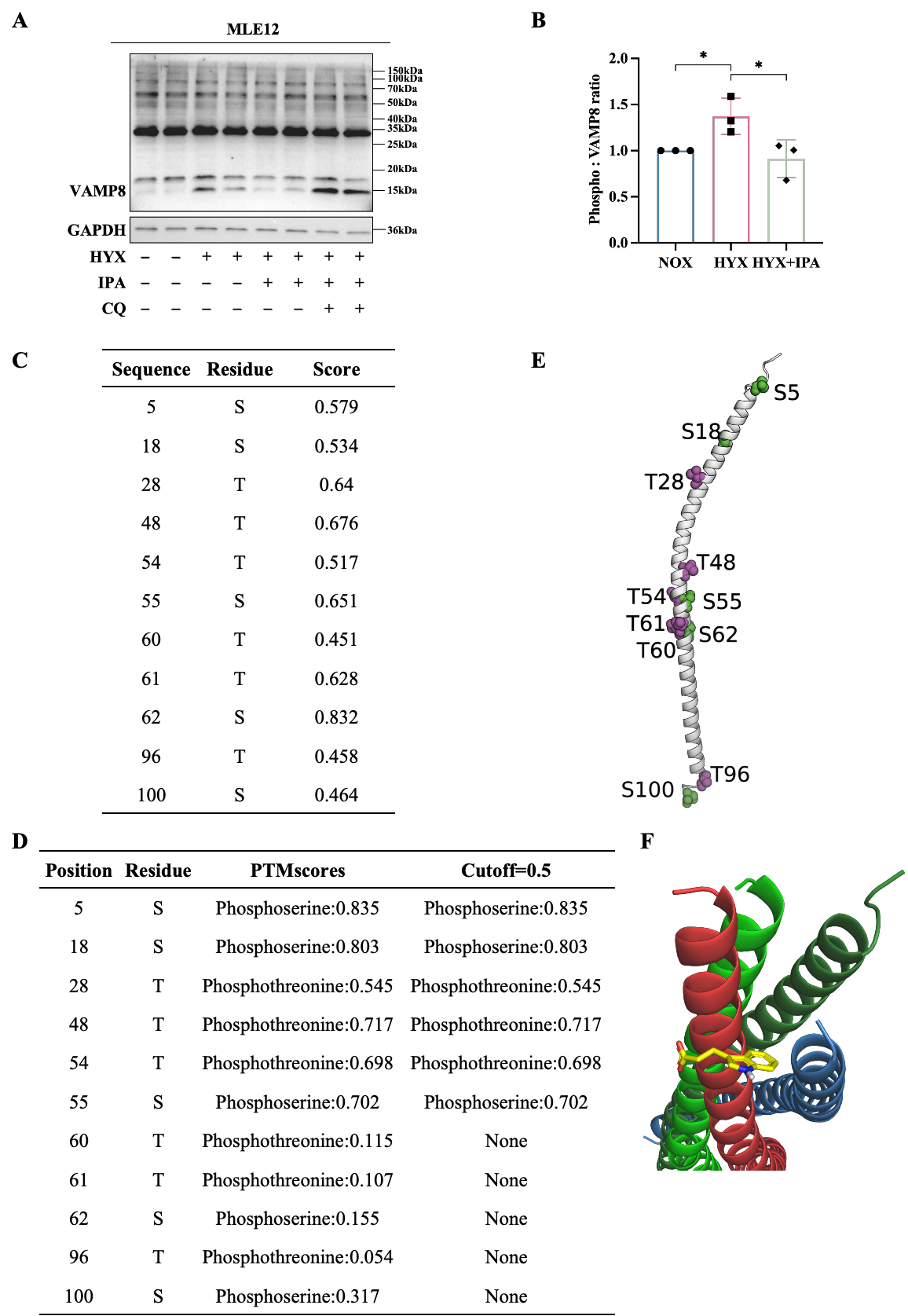


**Figure. S7**

1. Phosphorylation on protein was determined by western blots using anti-phospho antibody.
2. Densitometric analysis of VAMP8 phosphorylation in VAMP8 immunoprecipitates. Values represent the ratio of phospho to VAMP8 band intensity from three independent experiments. **P* < 0.05.

(C-D) Bioinformatic identification of Ser/Thr phosphorylation sites using (C) NetPhos-3.1 and (D) MusiteDeep algorithms.

(E) Spatial mapping of all putative phosphorylation sites within the VAMP8 protein structure.

(F) Structural modeling demonstrates specific interaction between IPA and the central phosphorylation domain of VAMP8.
