# Supplementary material for: Indole‐3‐Propionic Acid Improves Alveolar Development Impairment via Targeting VAMP8‐mediated SNAREs Complex Formation in Bronchopulmonary Dysplasia
Source: Adv Sci (Weinh). 2026 Feb 6;13(19):e02610. doi: 10.1002/advs.202502610 (PMC13045412; doi:10.1002/advs.202502610)

Full unedited gel for Figure 3";

Full unedited gel for Figure 3G";

(1)

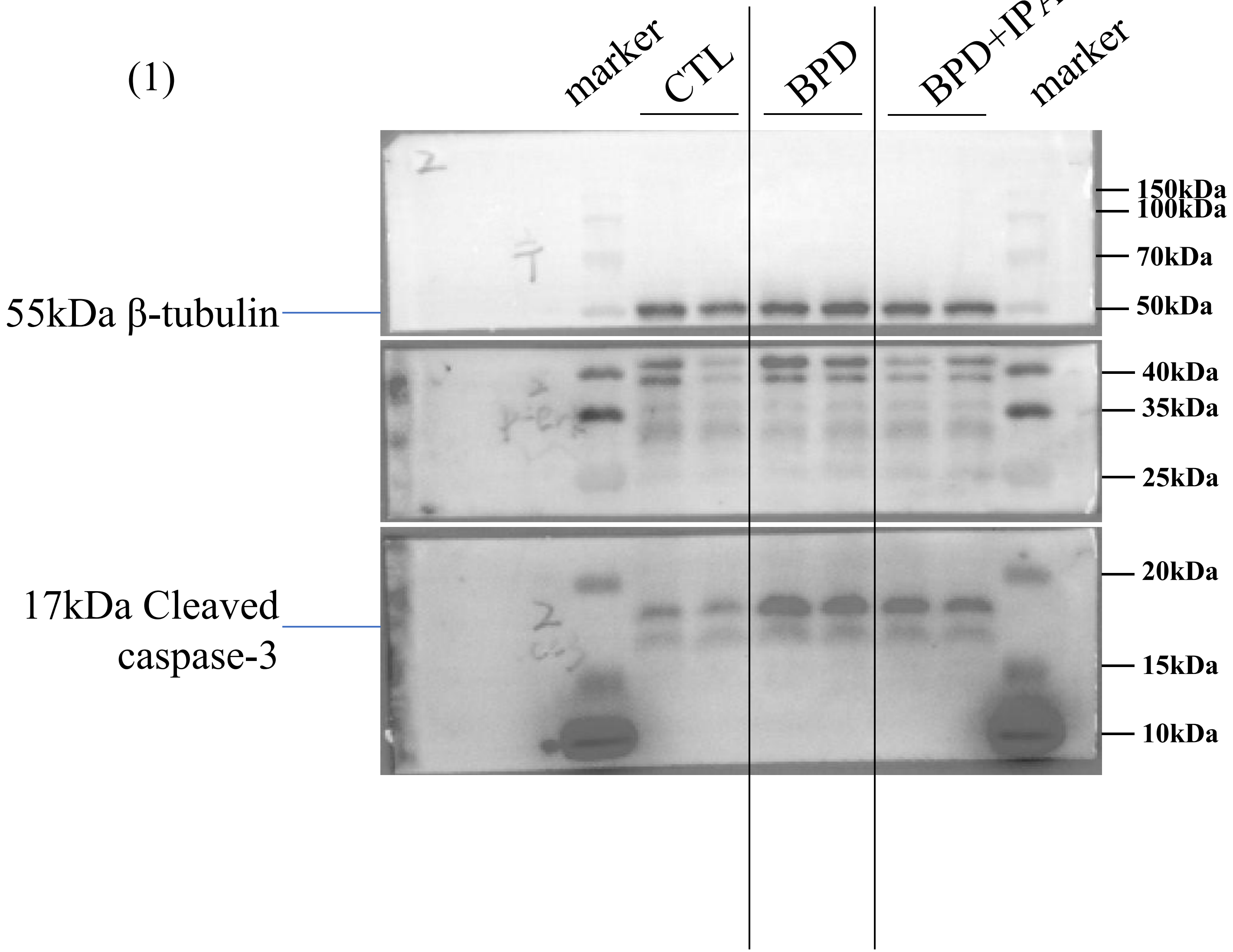

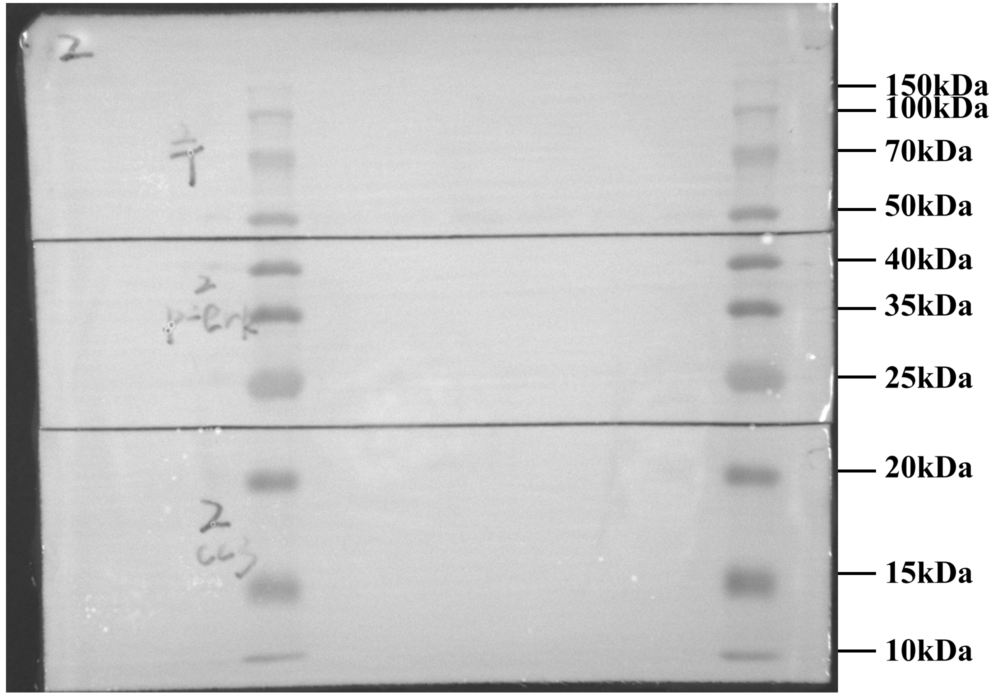

Full unedited gel for Figure 3G";

(2)

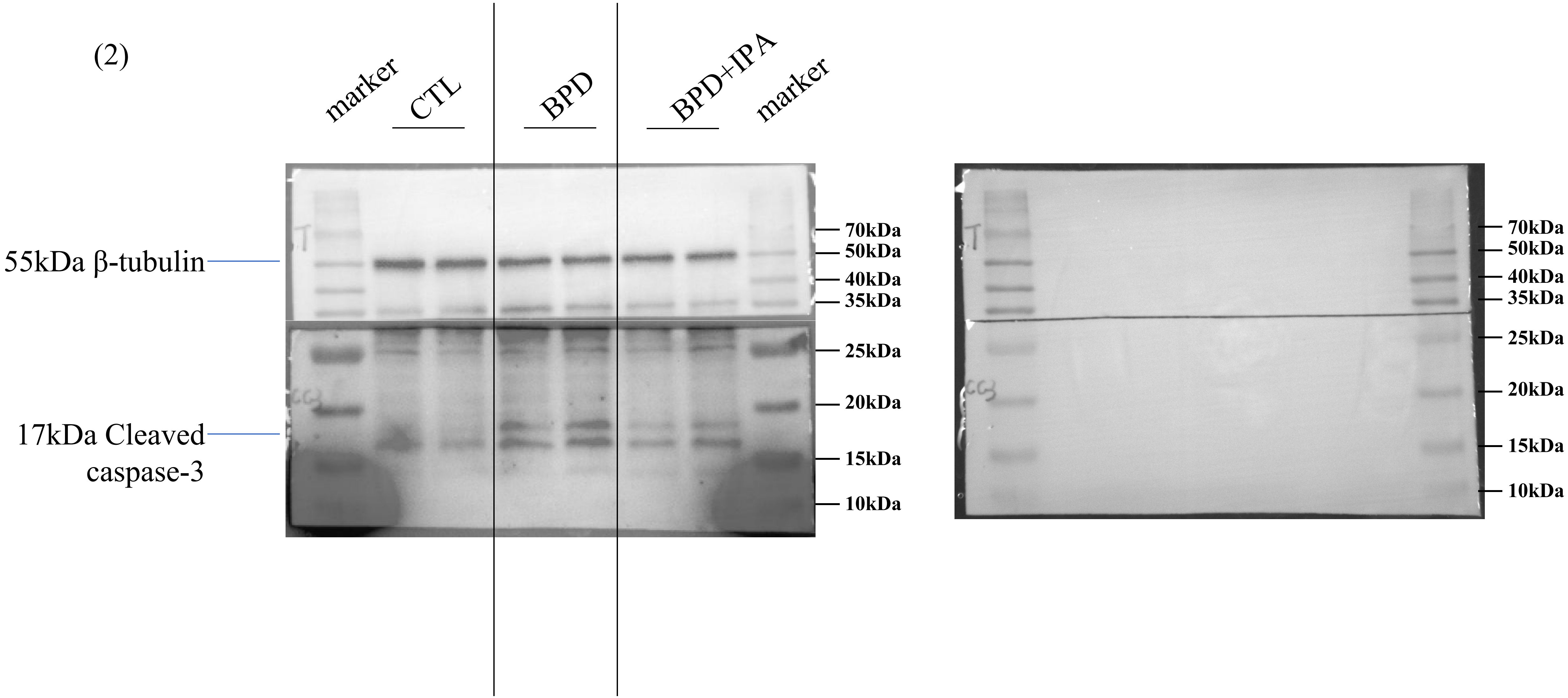

Full unedited gel for Figure 3G";

(3)

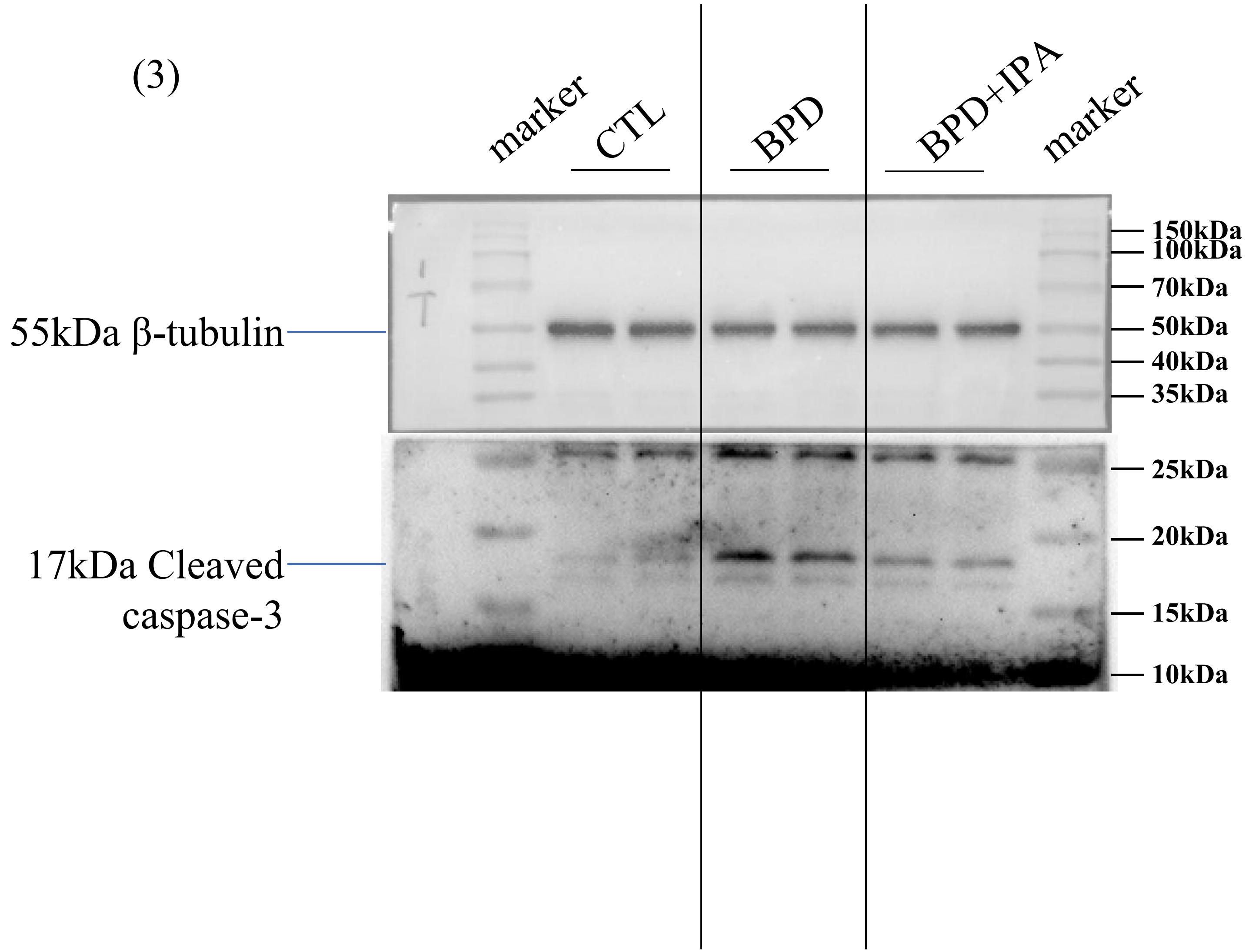

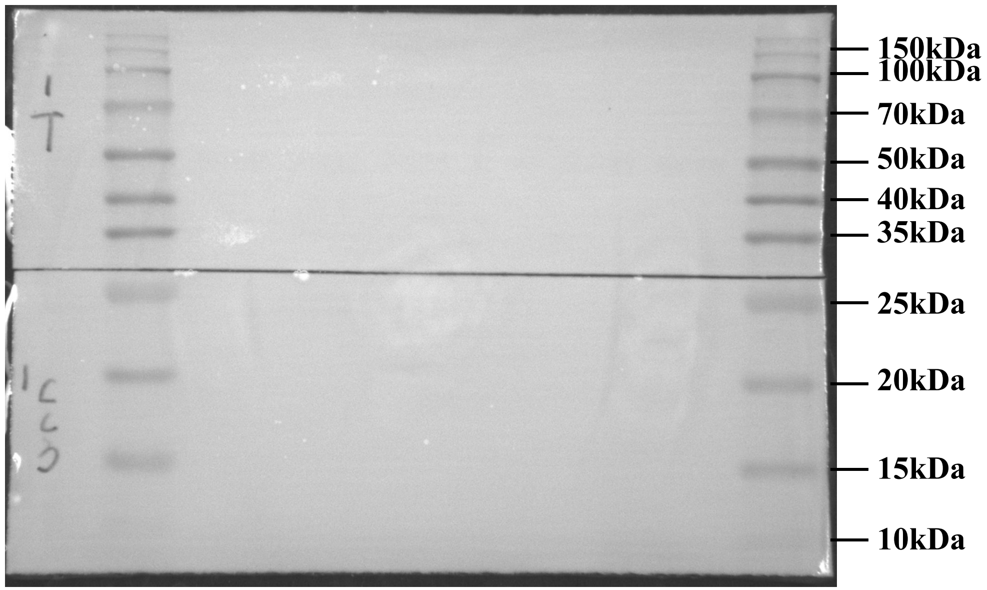

Full unedited gel for Figure 3N";

(1)

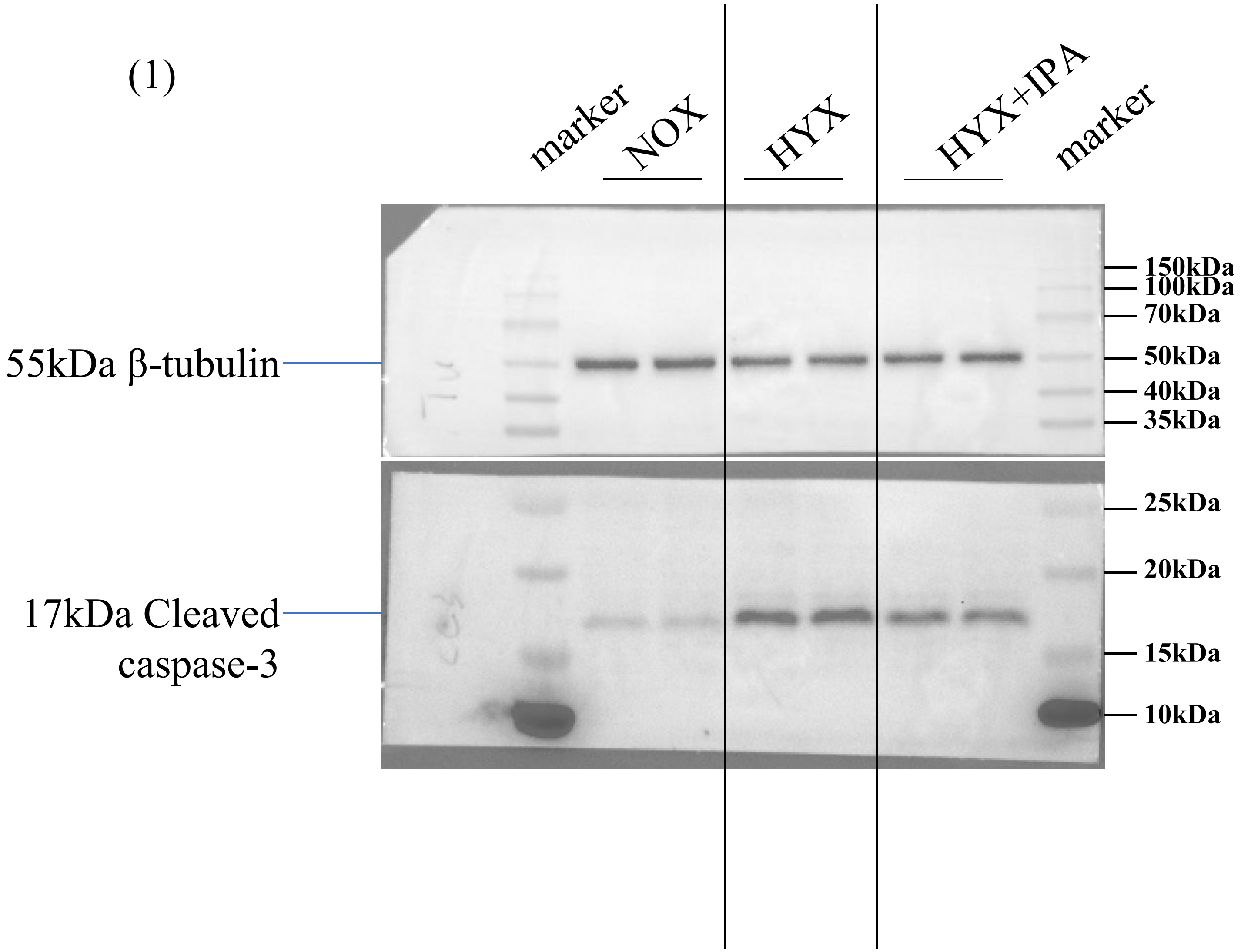

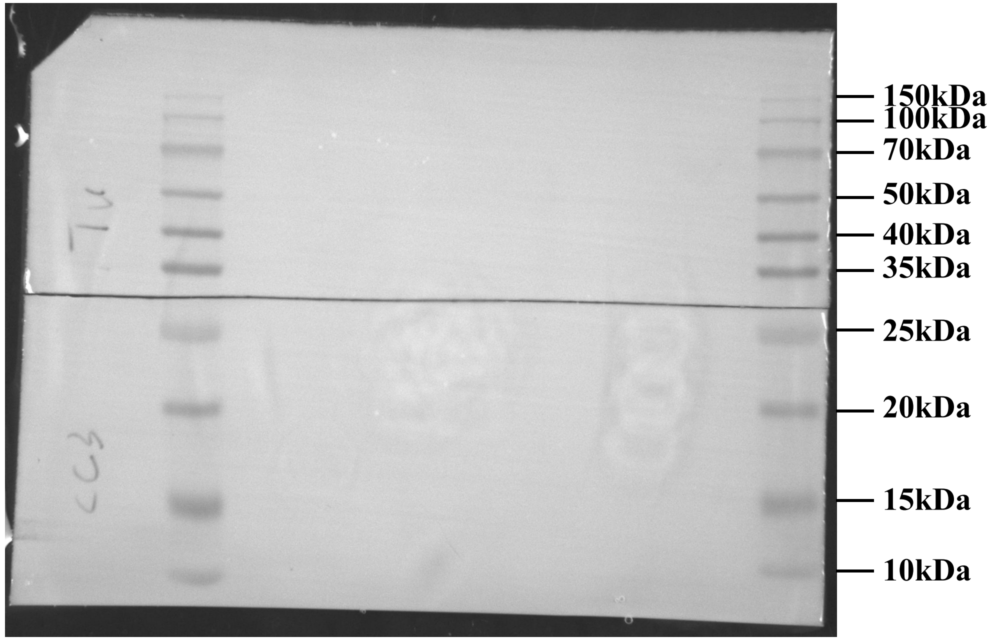

Full unedited gel for Figure 3N";

(2)

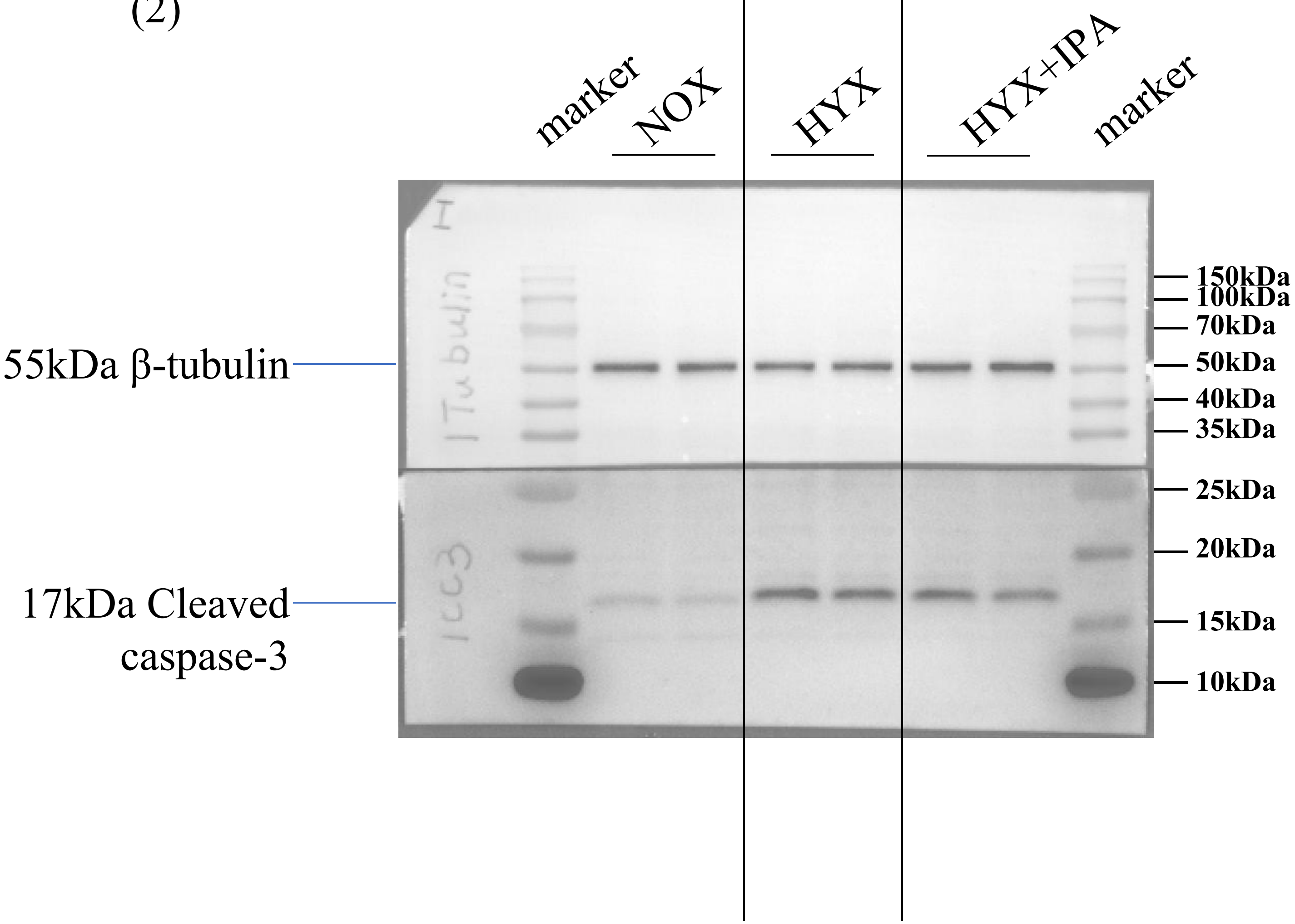

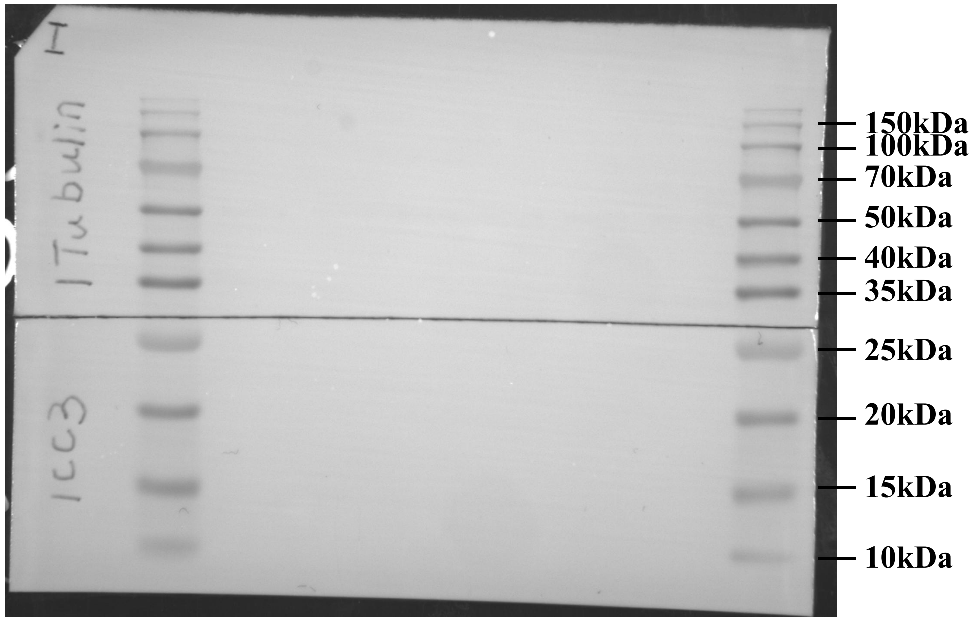

Full unedited gel for Figure 3N";

(3)

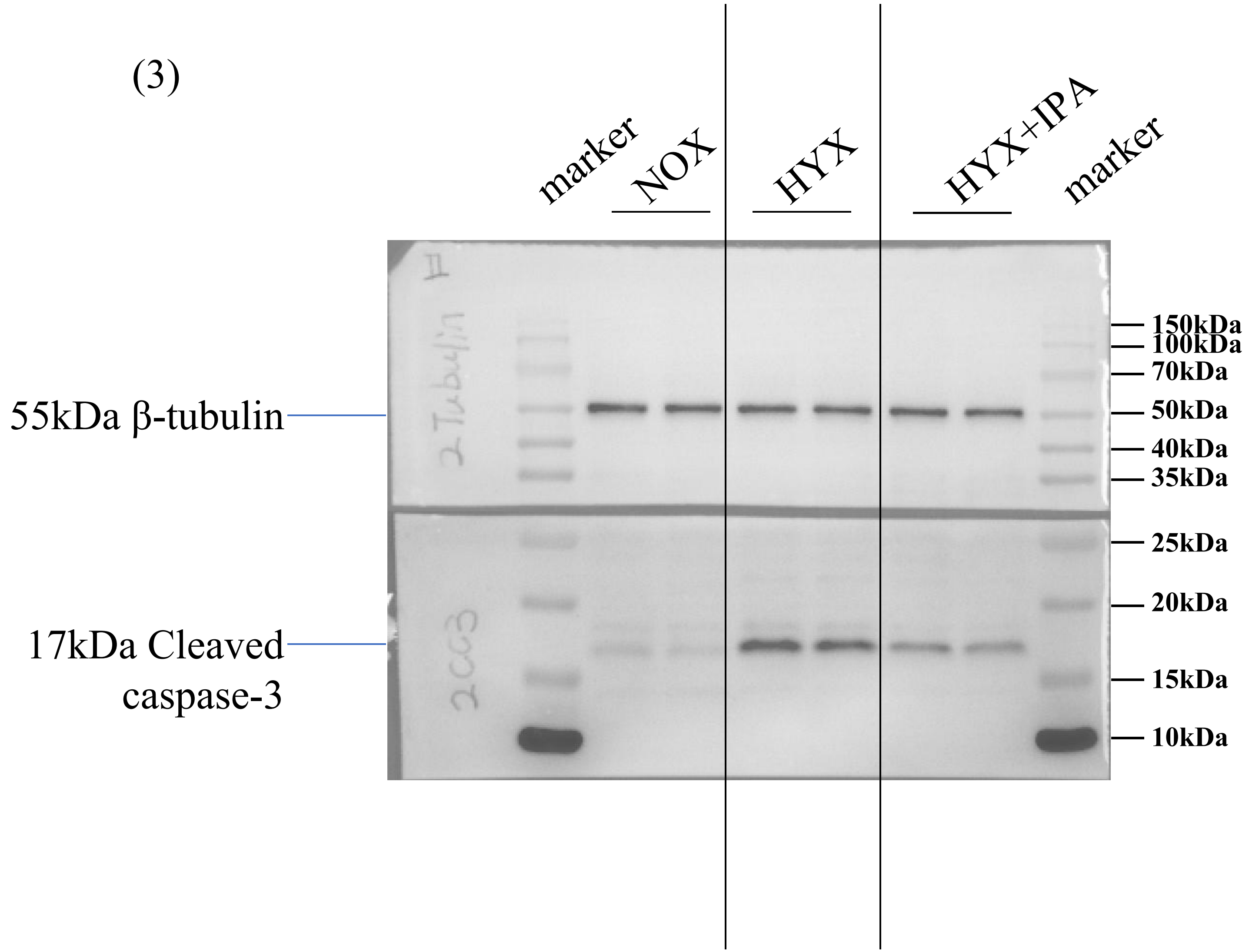

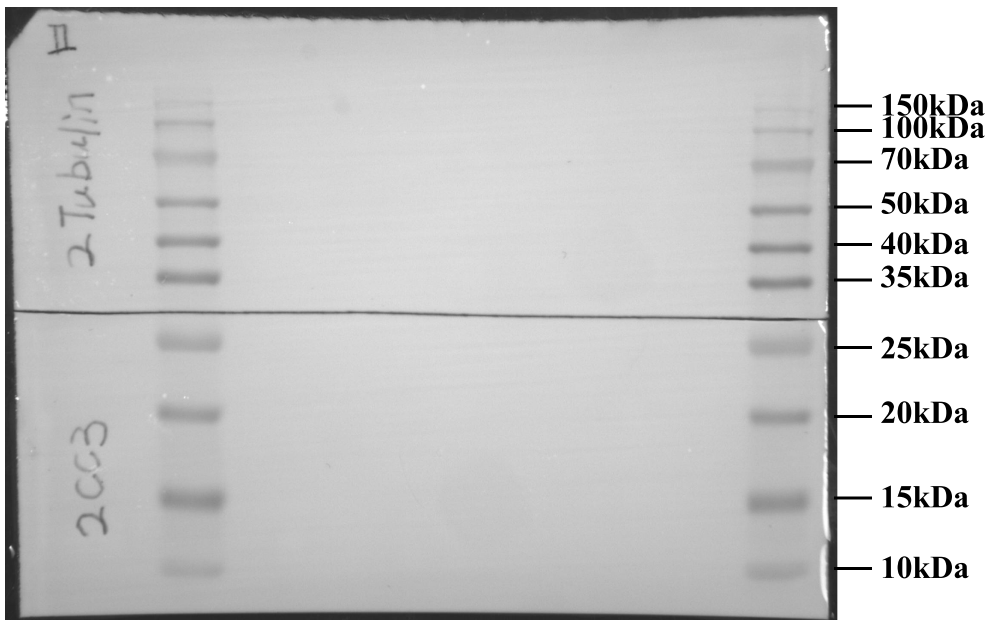

Full unedited gel for Figure 4";

Full unedited gel for Figure 4D";

(1)

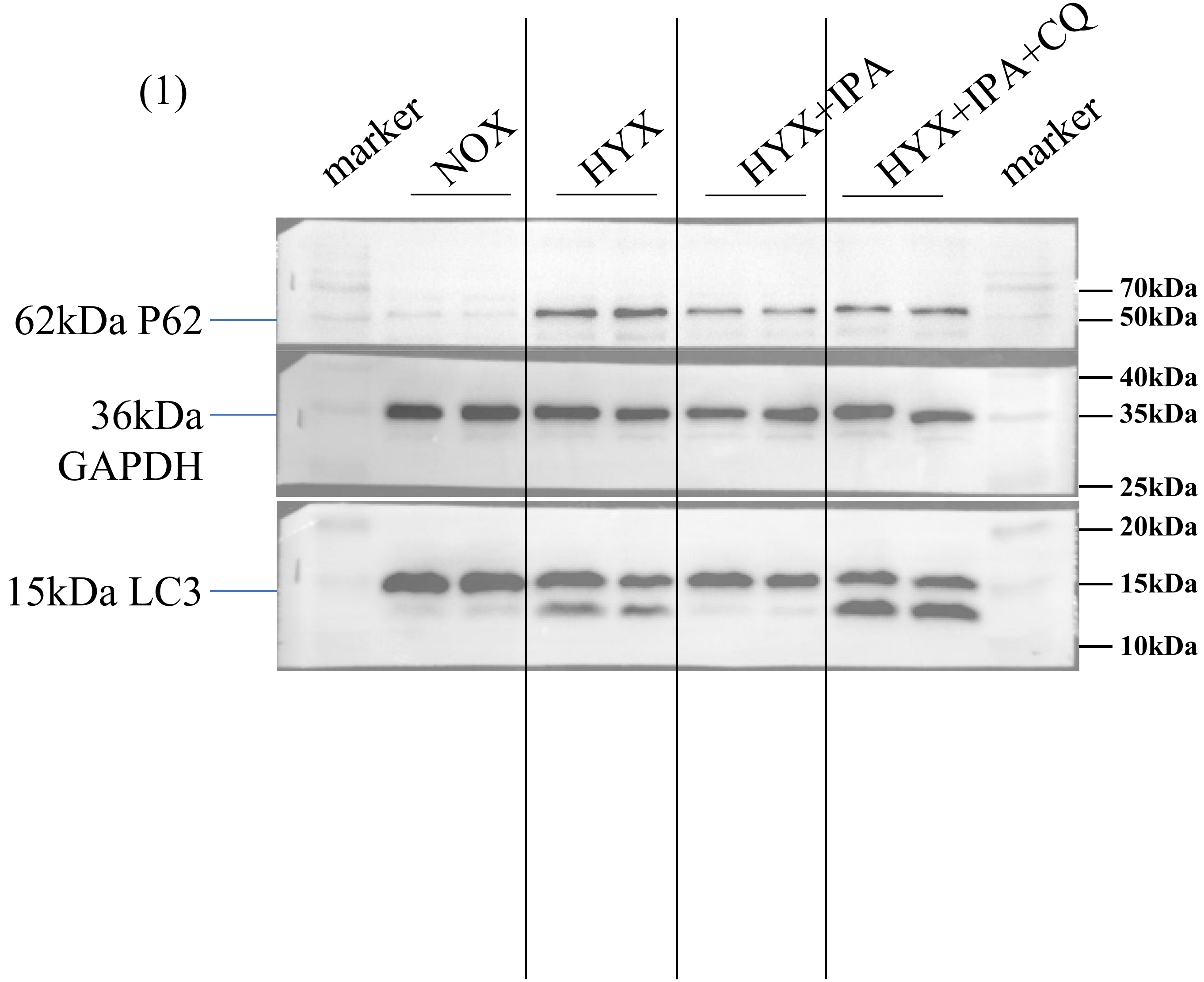

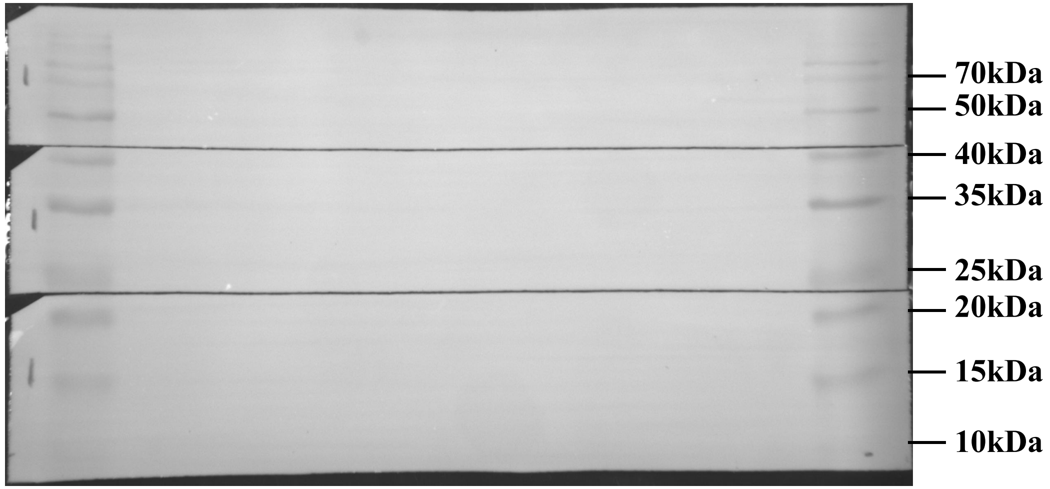

Full unedited gel for Figure 4D";

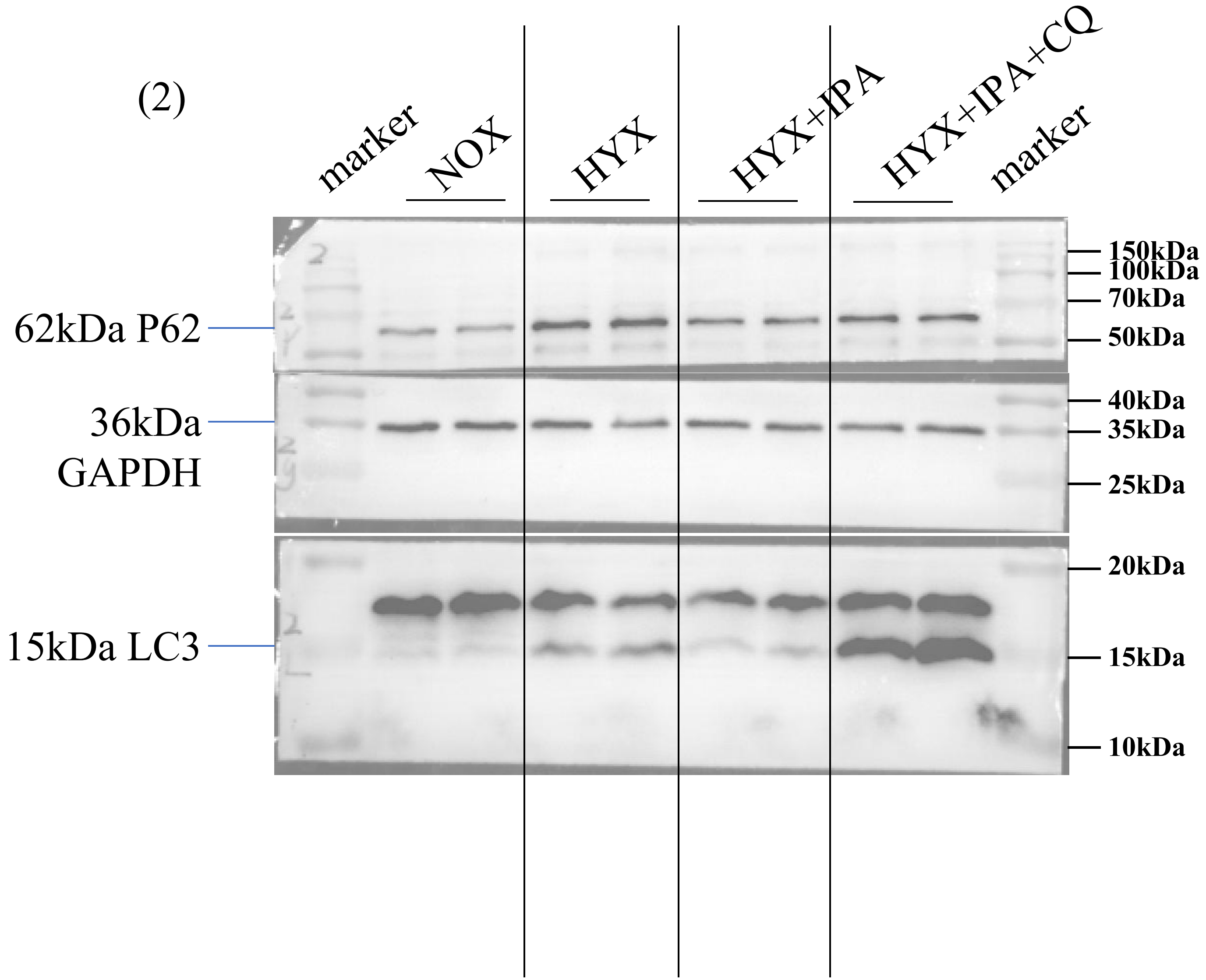

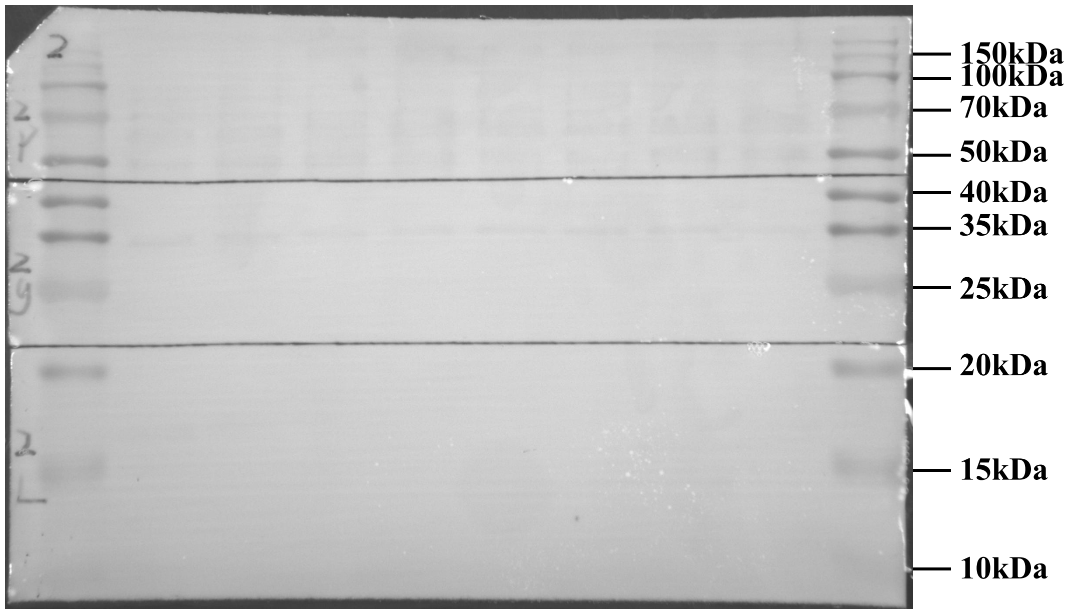

Full unedited gel for Figure 4D";

(3)

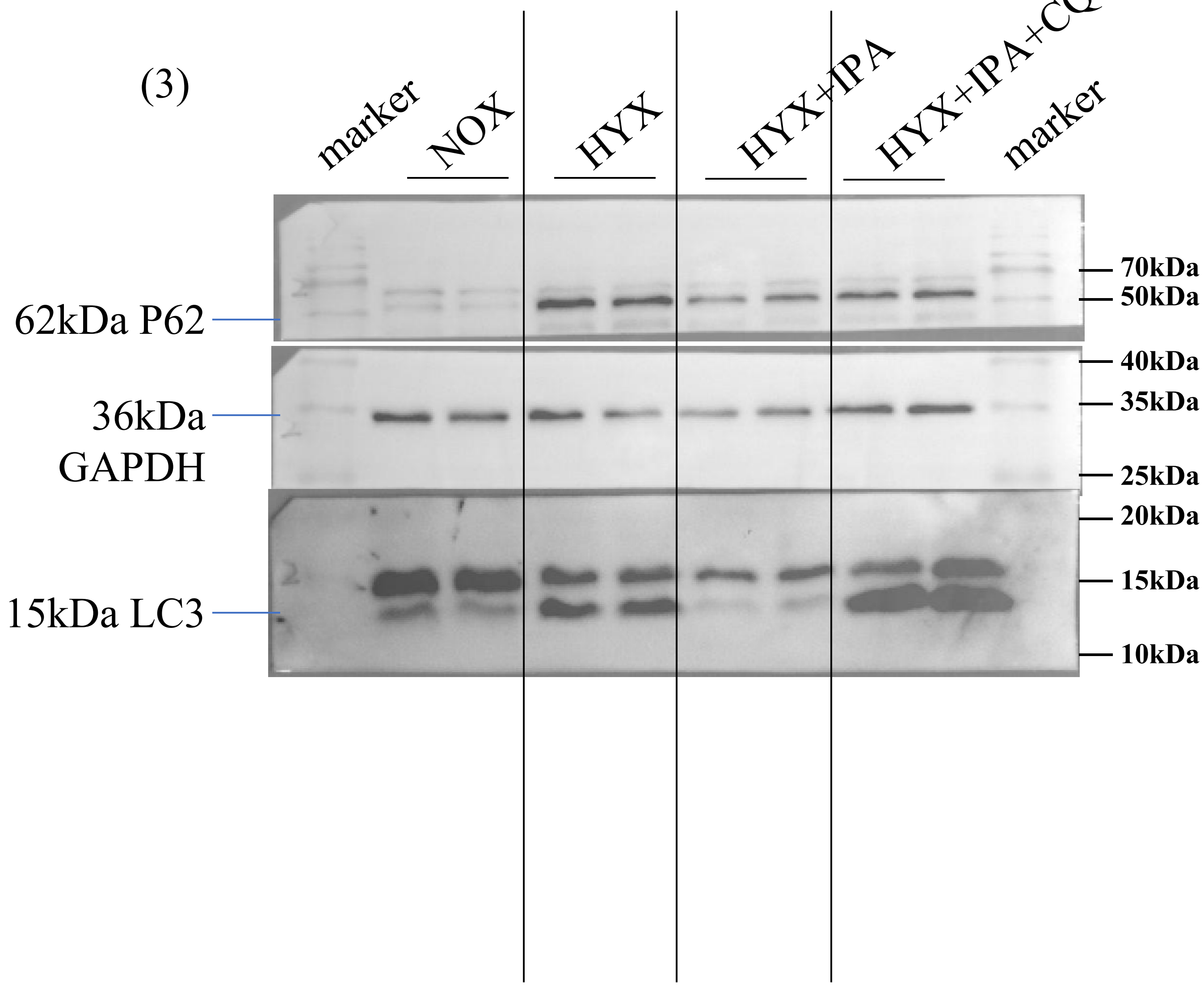

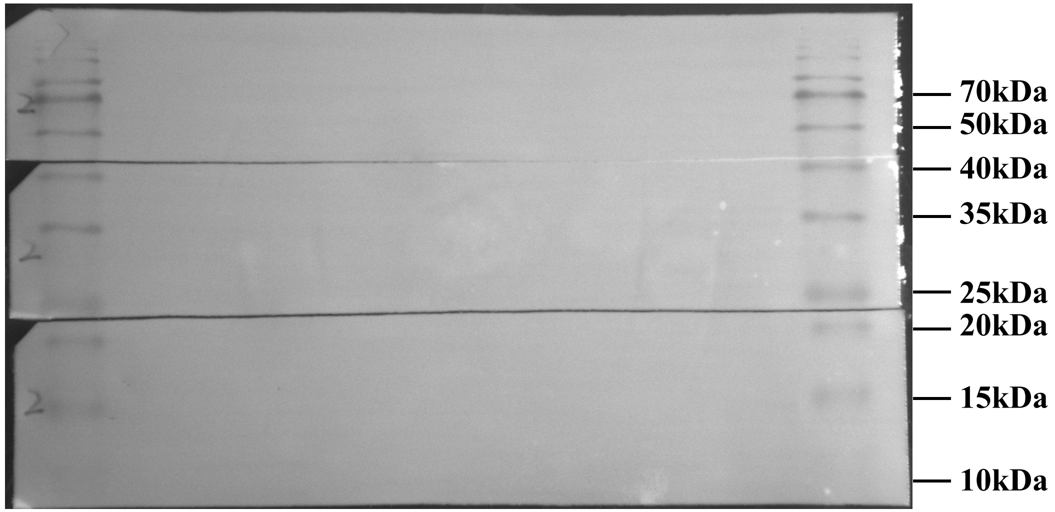

Full unedited gel for Figure 5";

Full unedited gel for Figure 5A";

(1)

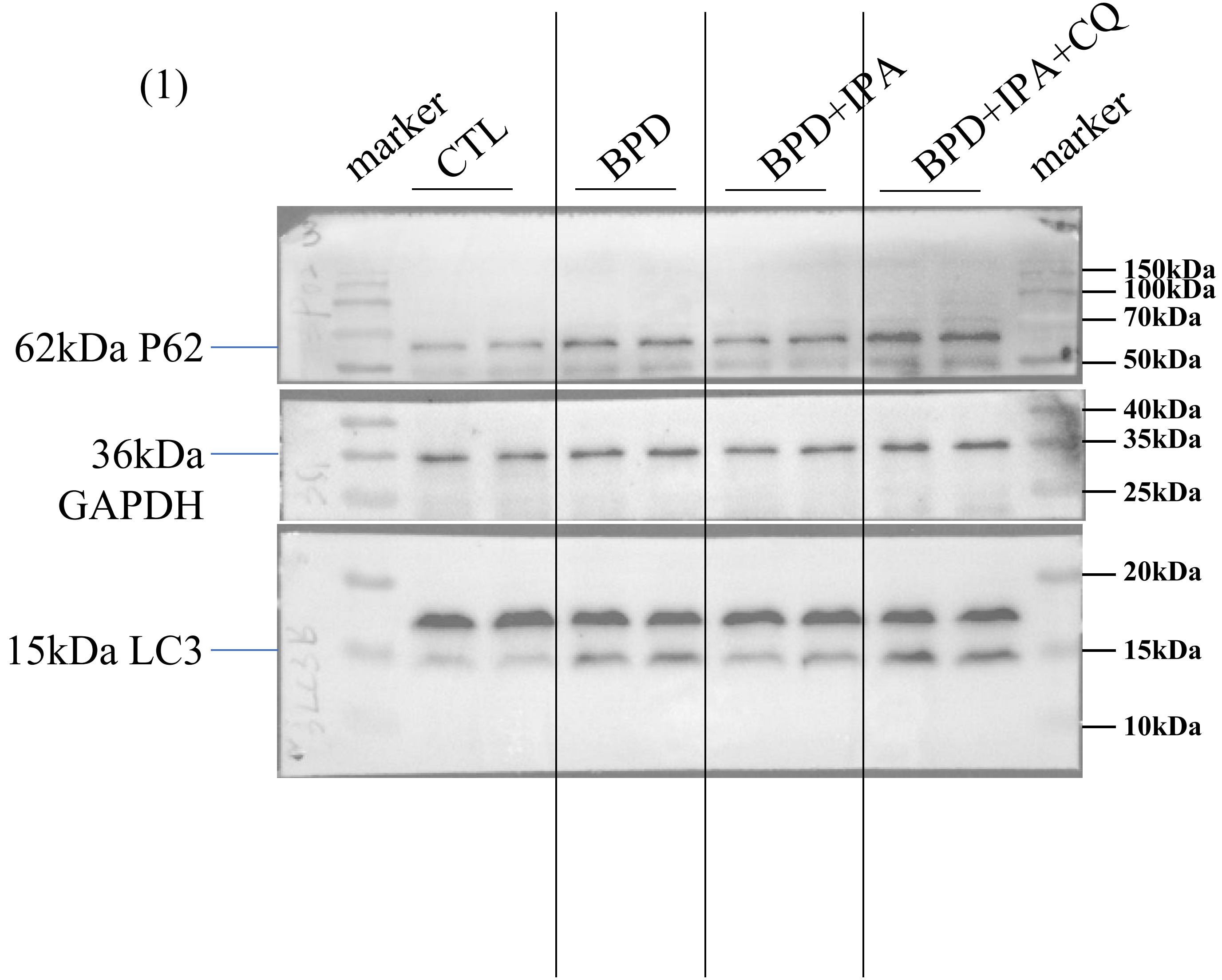

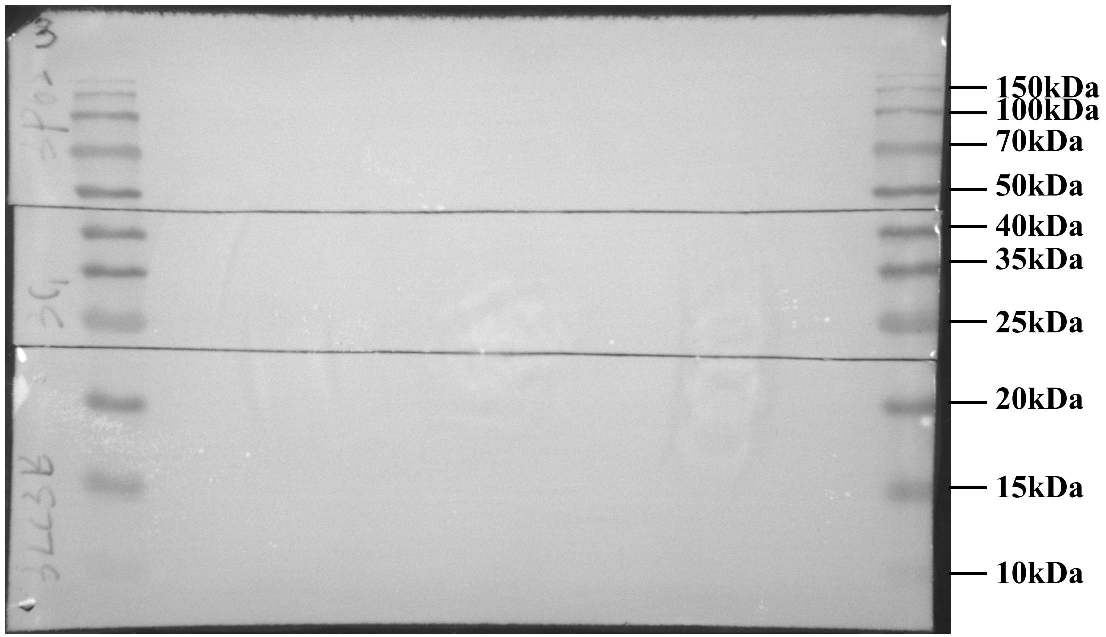

Full unedited gel for Figure 5A";

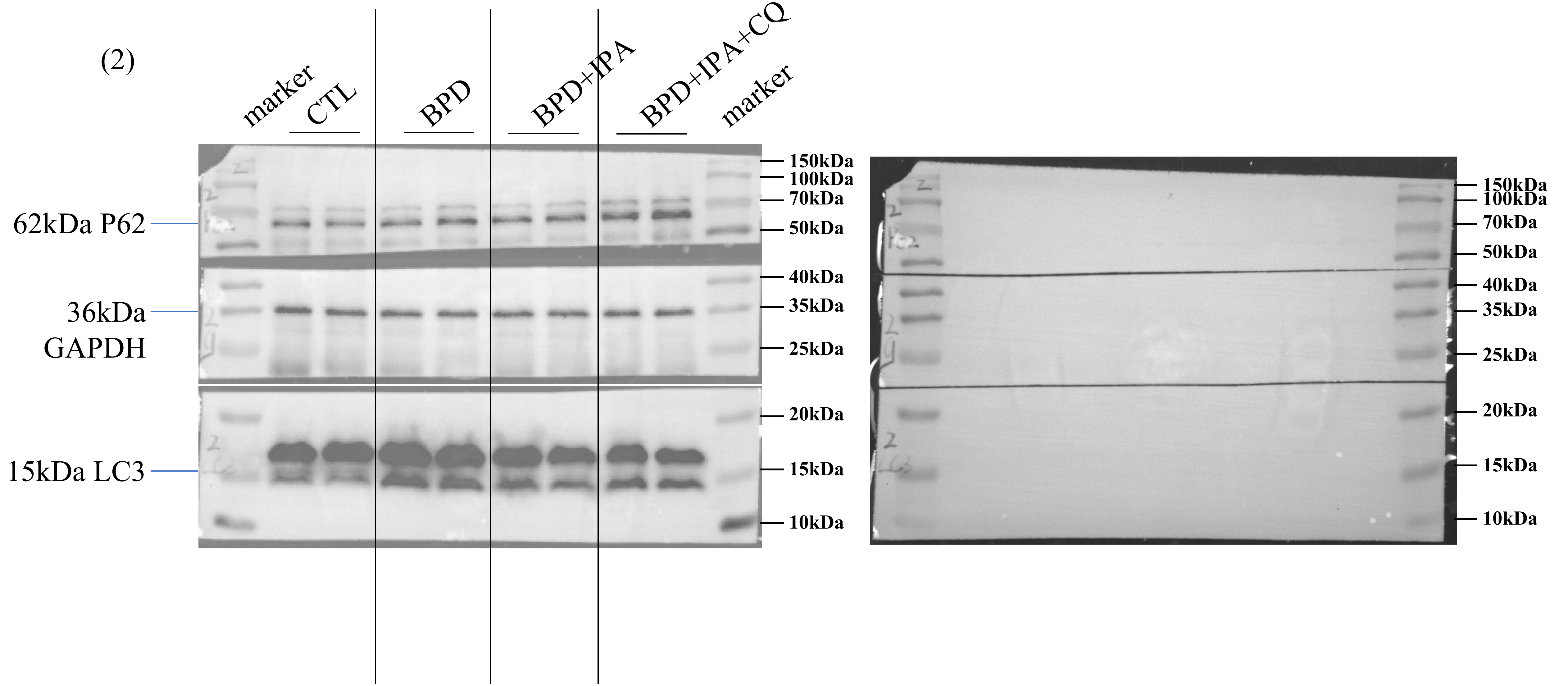

Full unedited gel for Figure 5A";

(3)

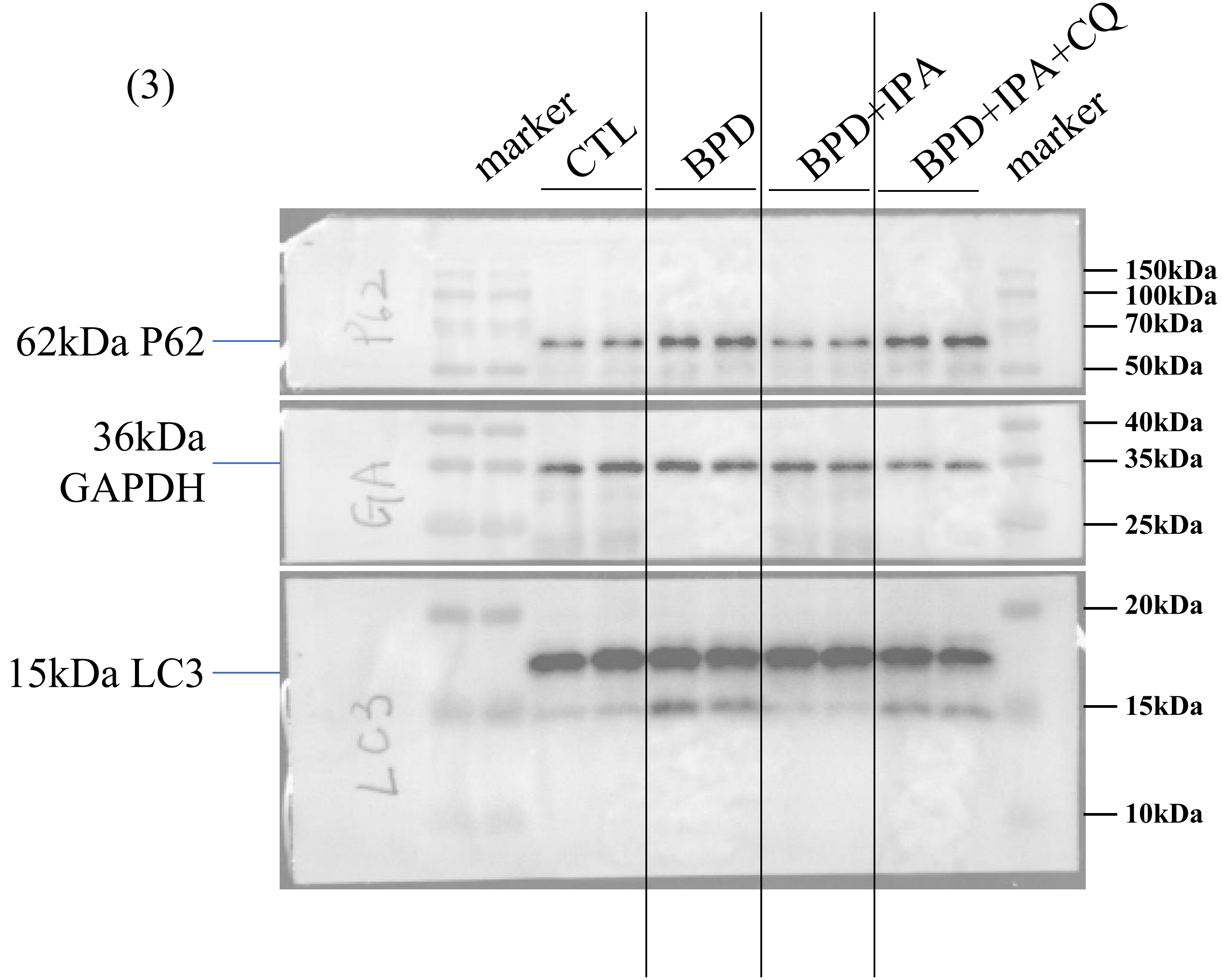

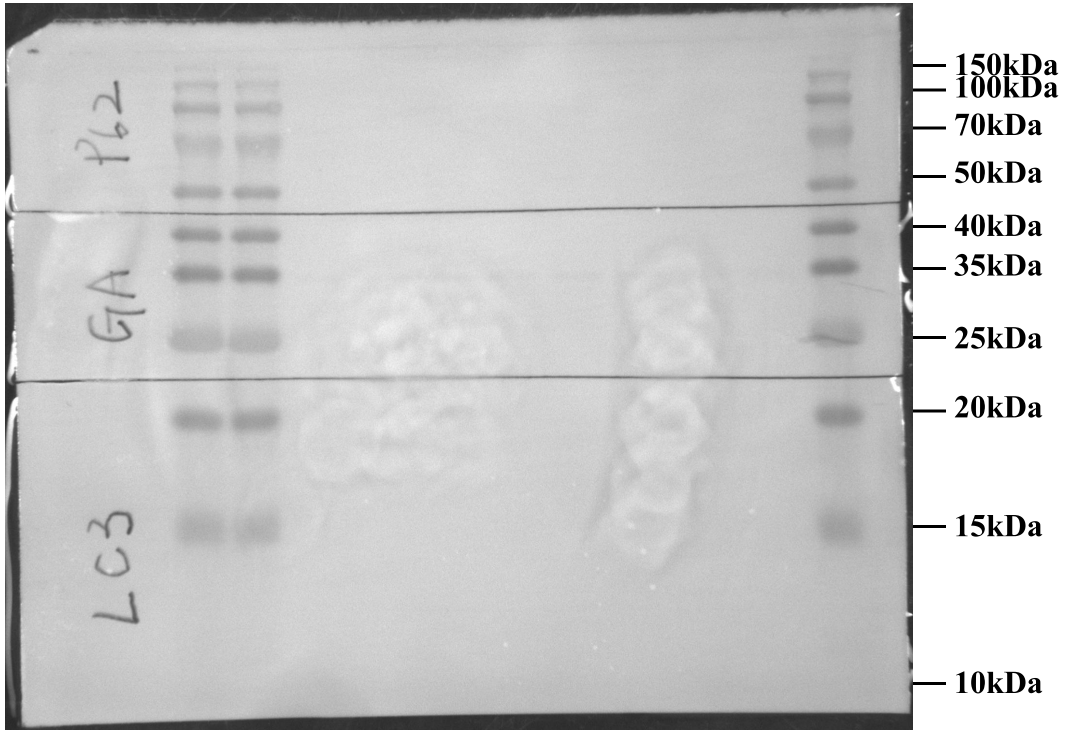

Full unedited gel for Figure 5F";

(1)

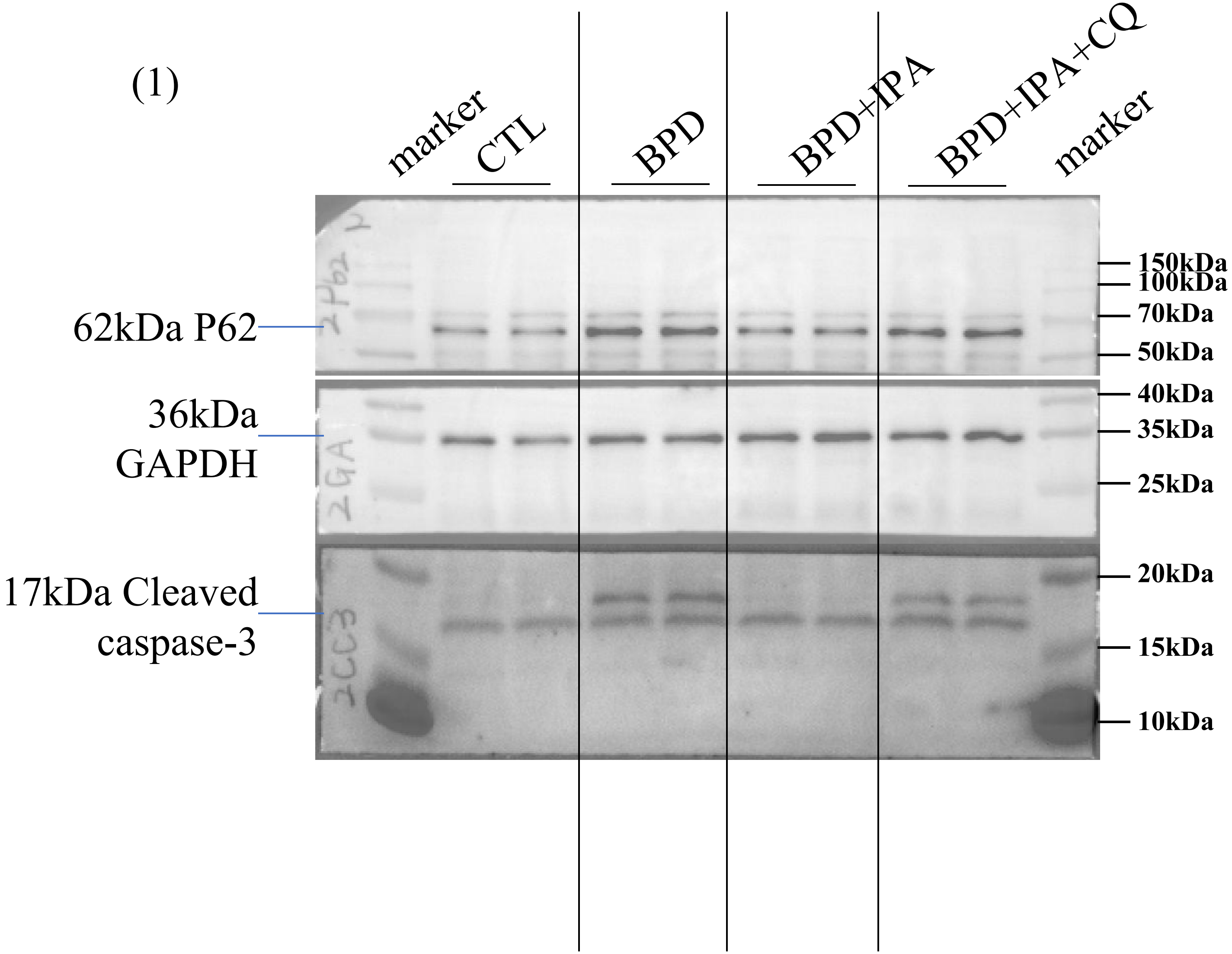

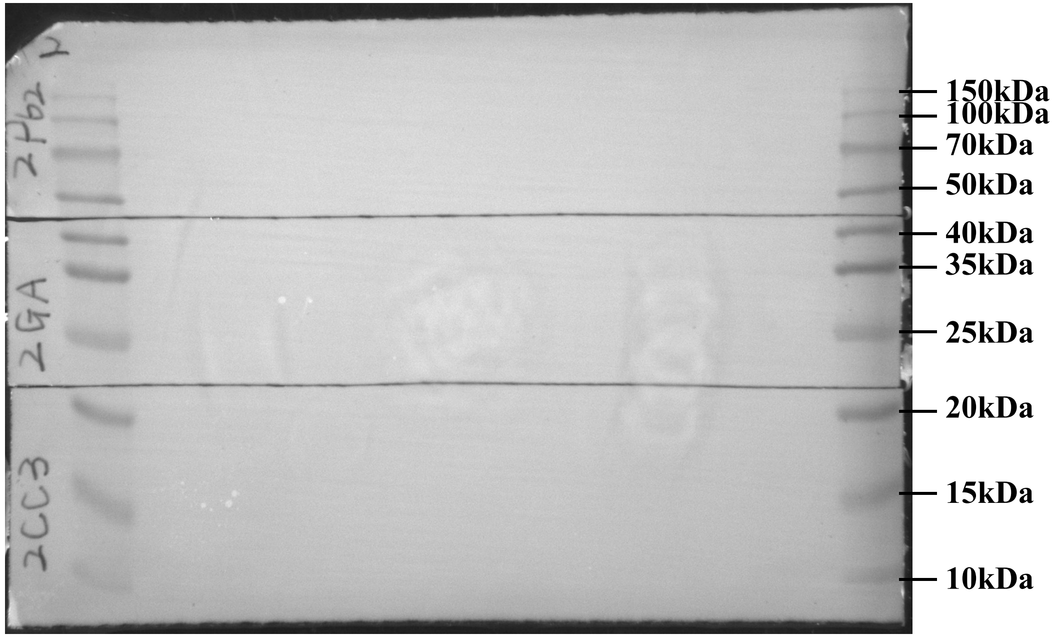

Full unedited gel for Figure 5F";

(2)

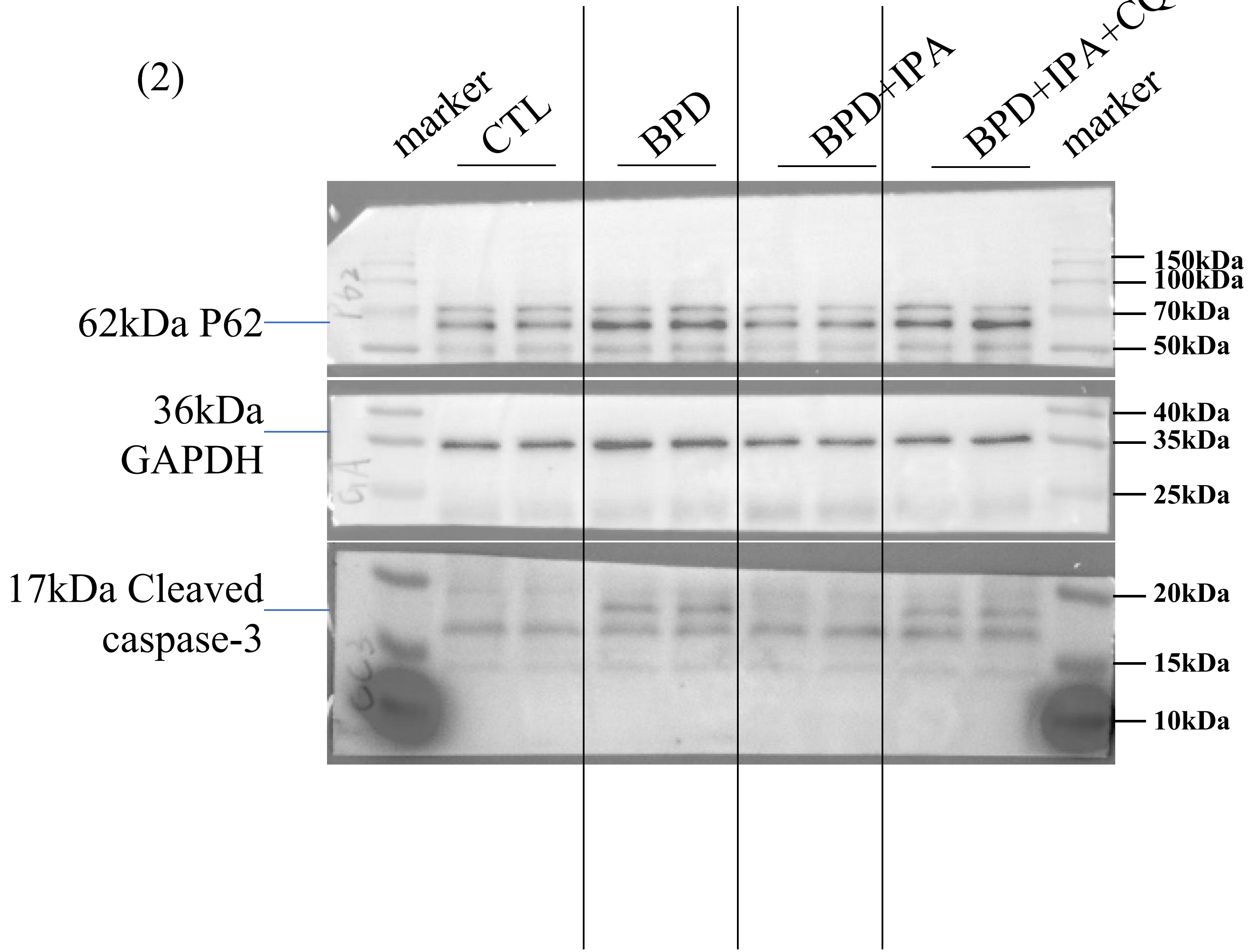

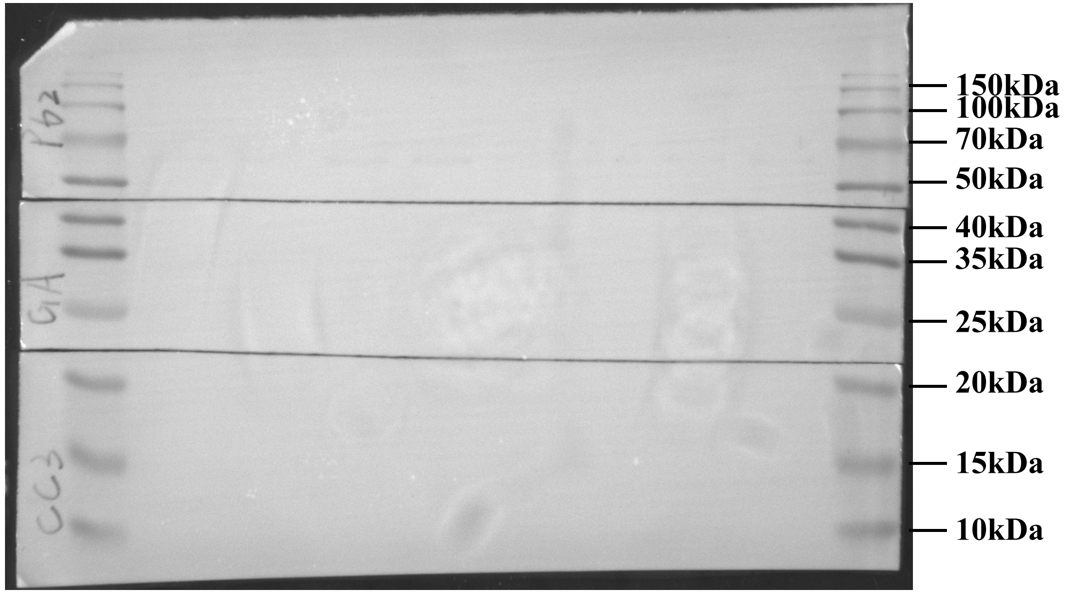

Full unedited gel for Figure 5F";

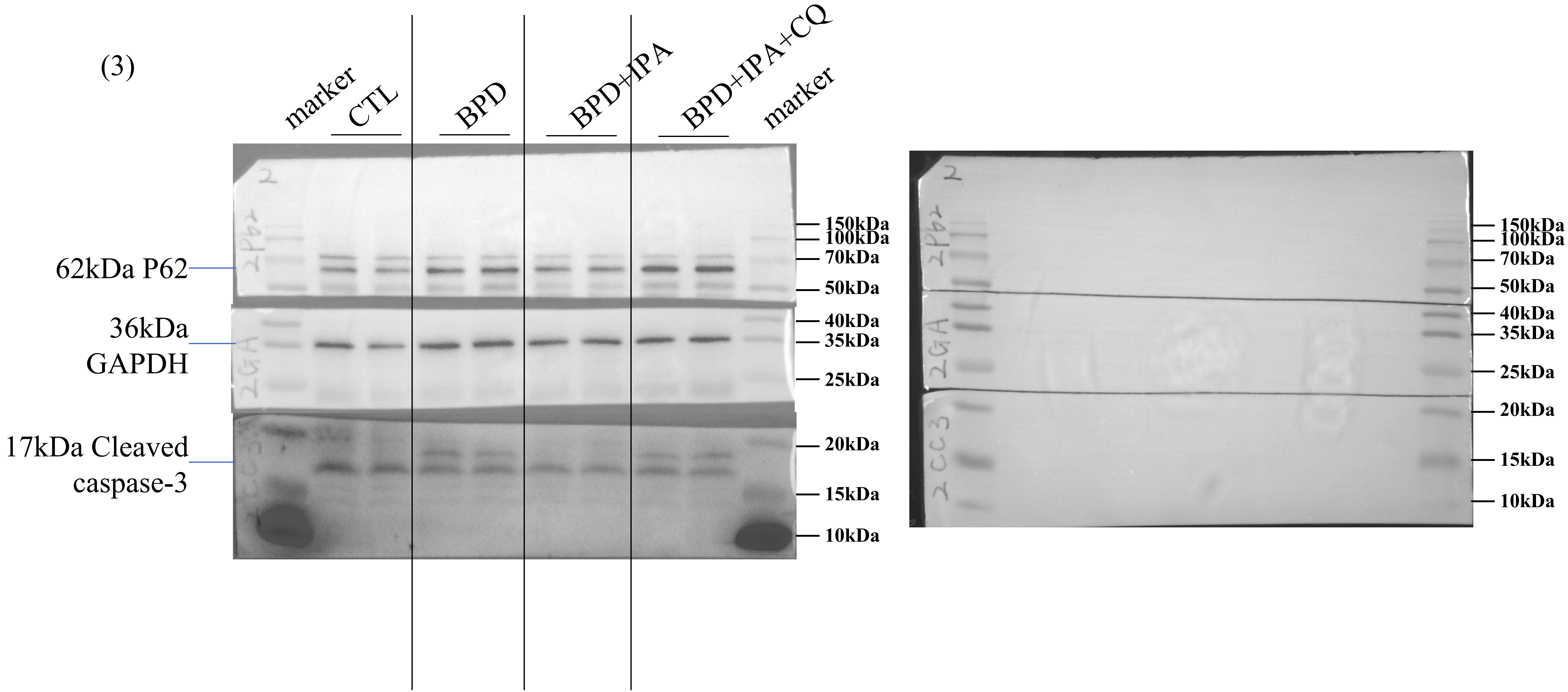

Full unedited gel for Figure 6";

Full unedited gel for Figure 6I";

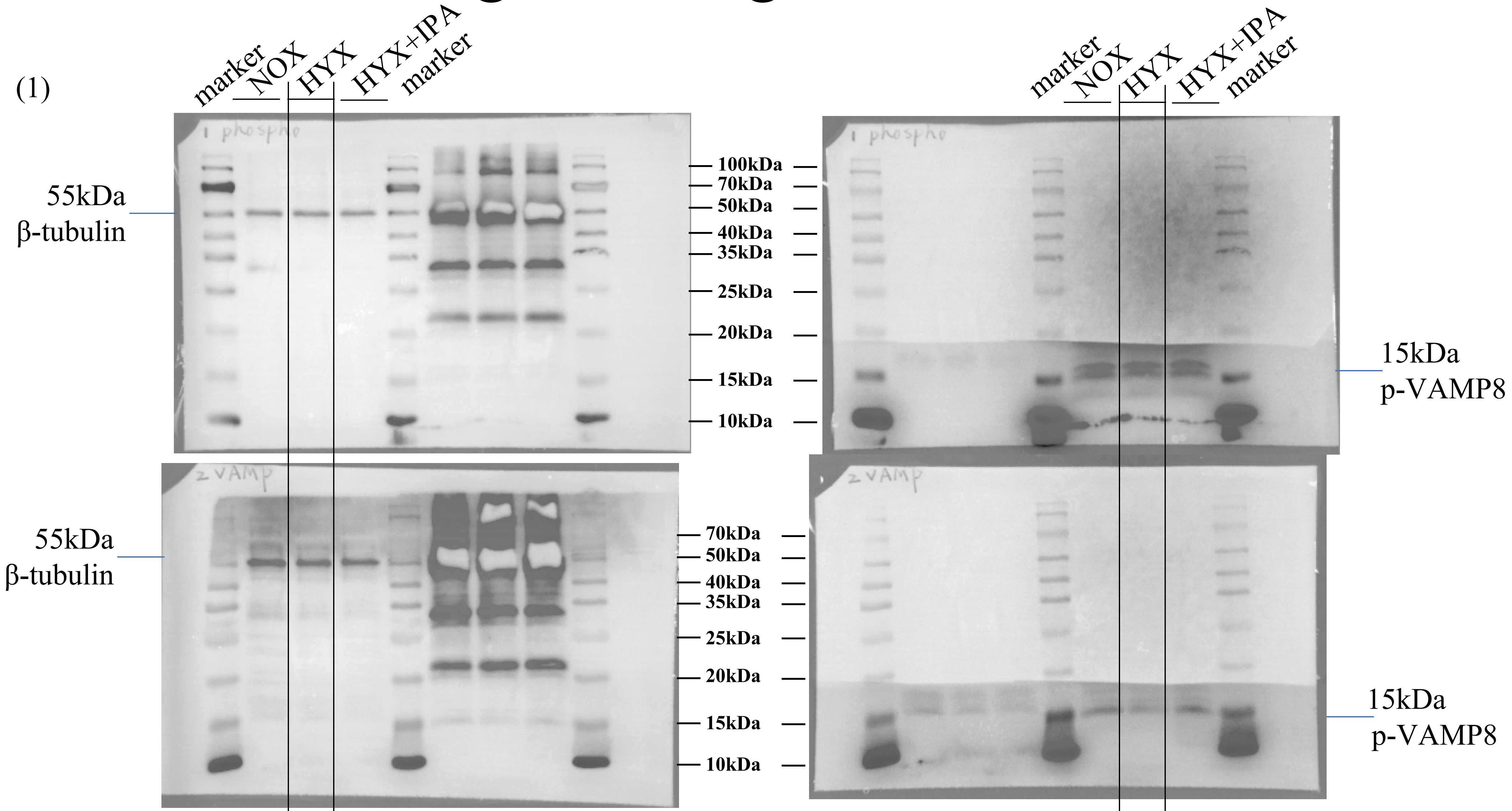

Full unedited gel for Figure 6I";

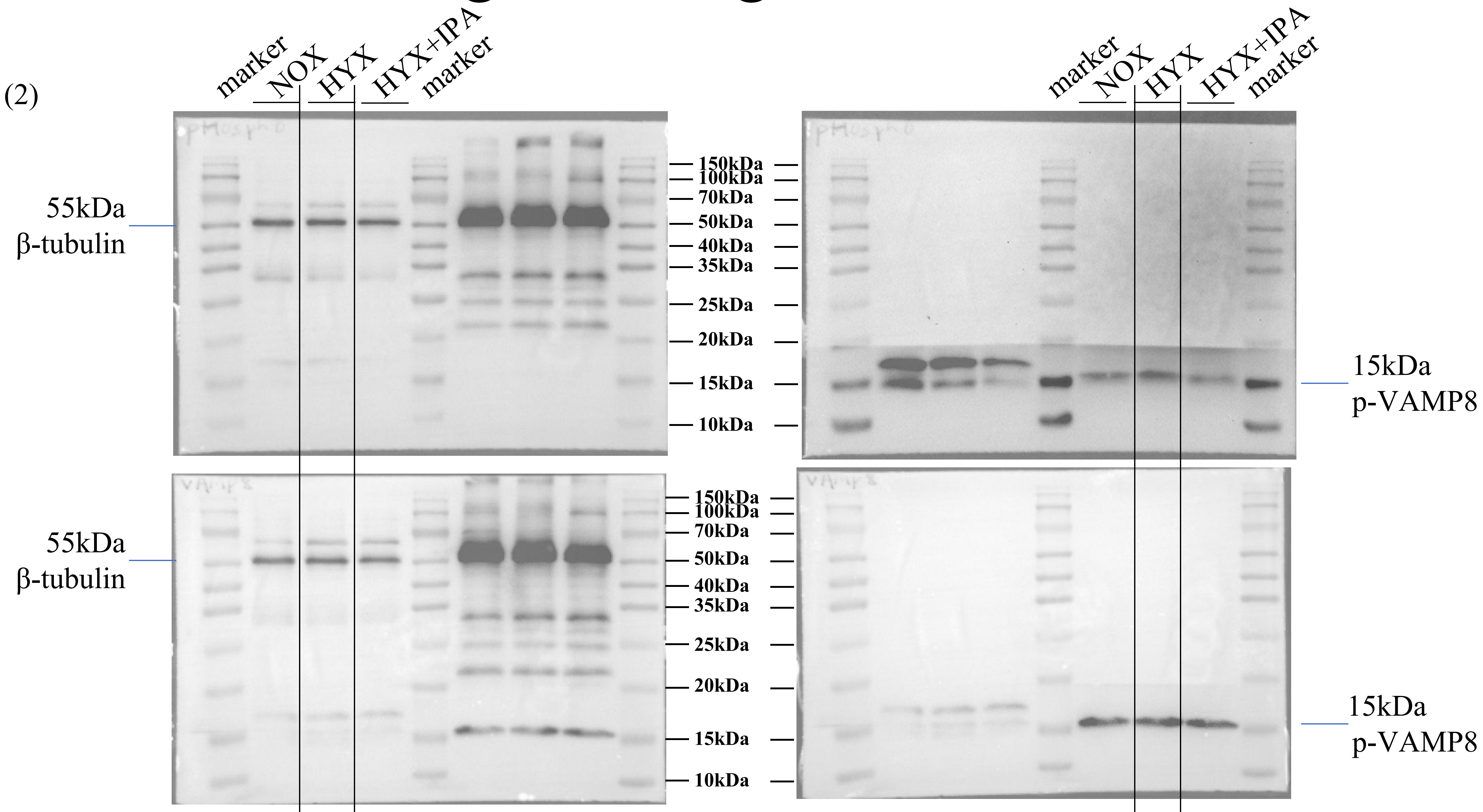

Full unedited gel for Figure 6I";

(3)

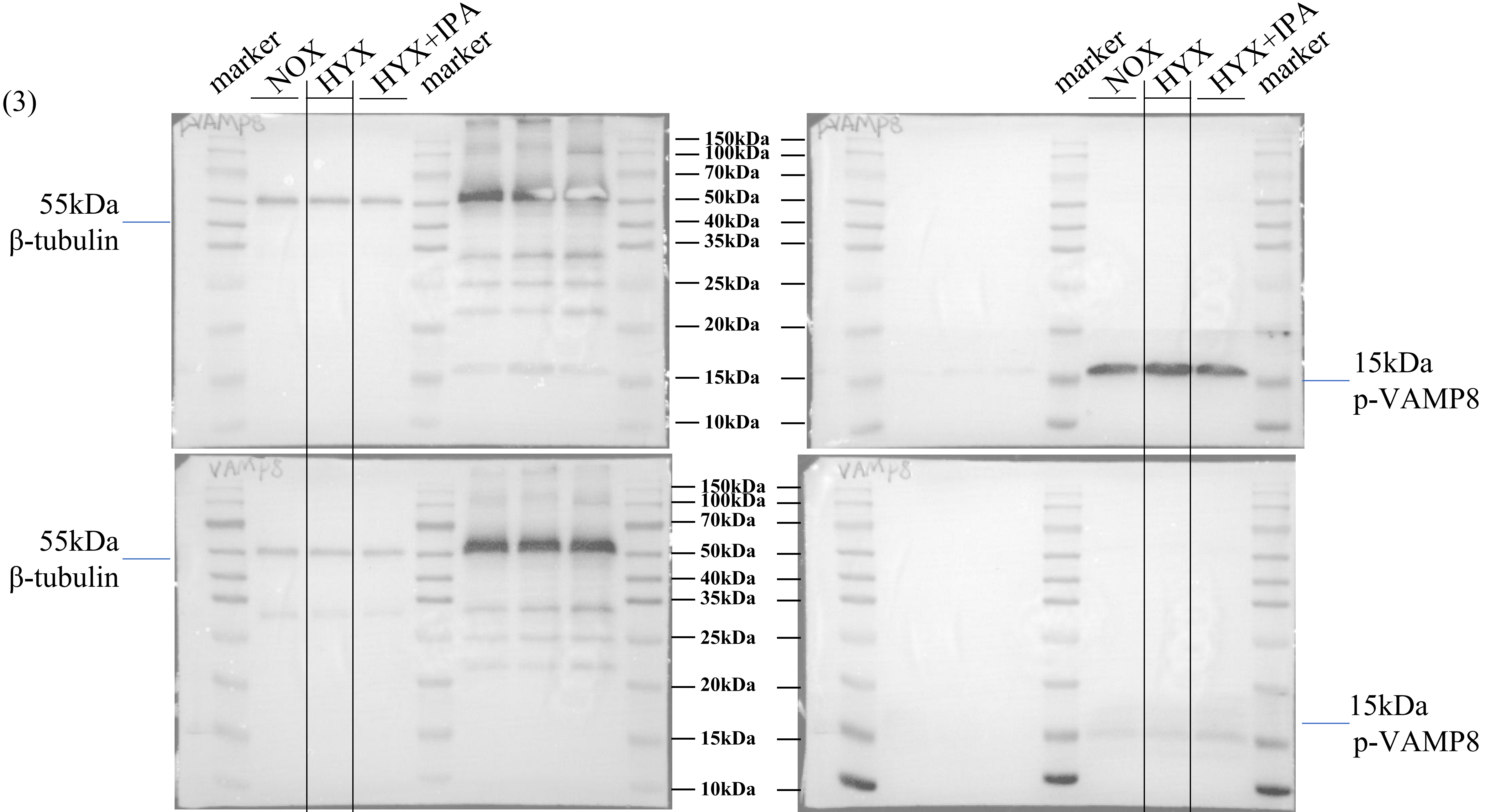

Full unedited gel for Figure S2";

Full unedited gel for Figure S2F";

(1)

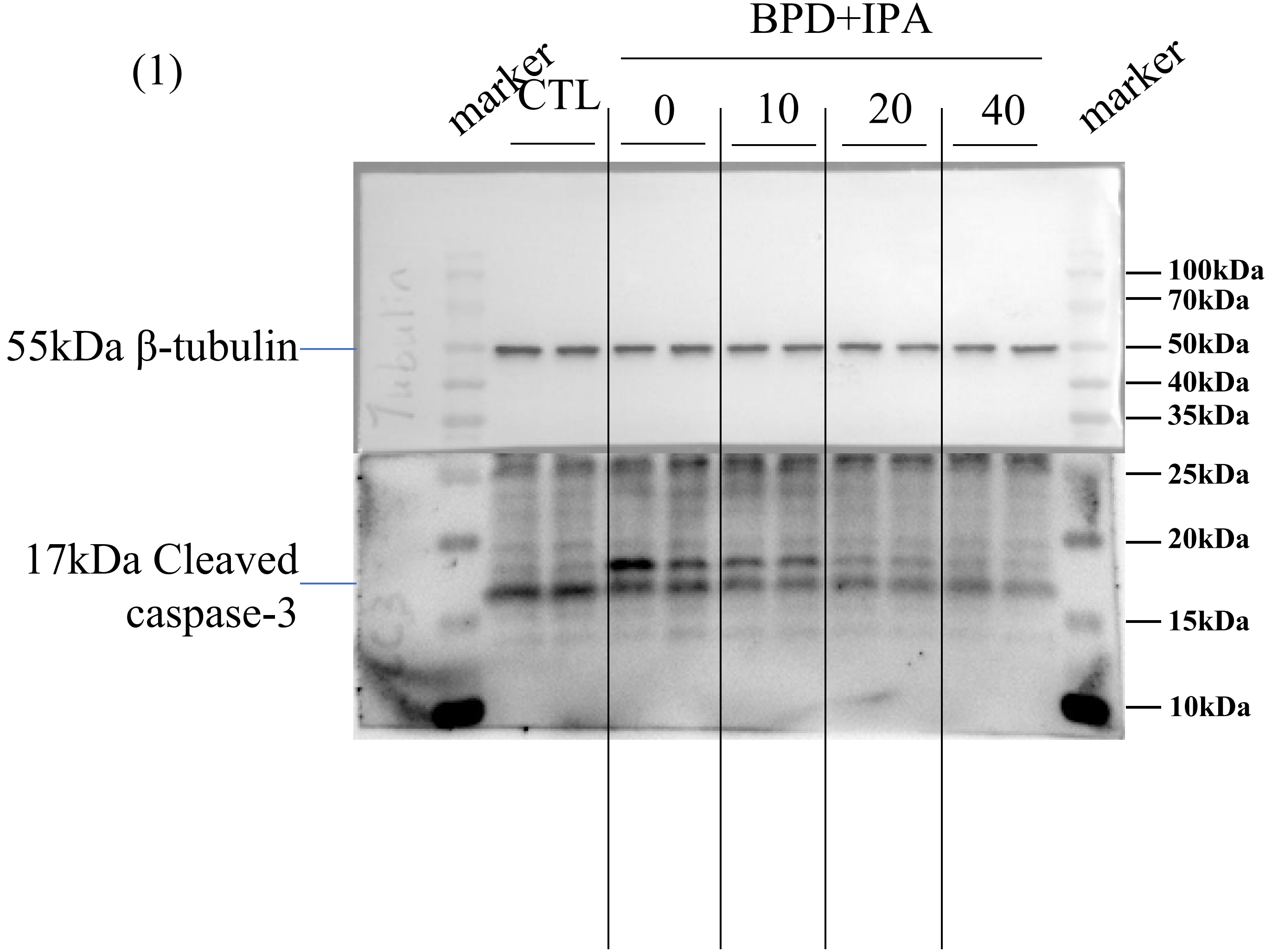

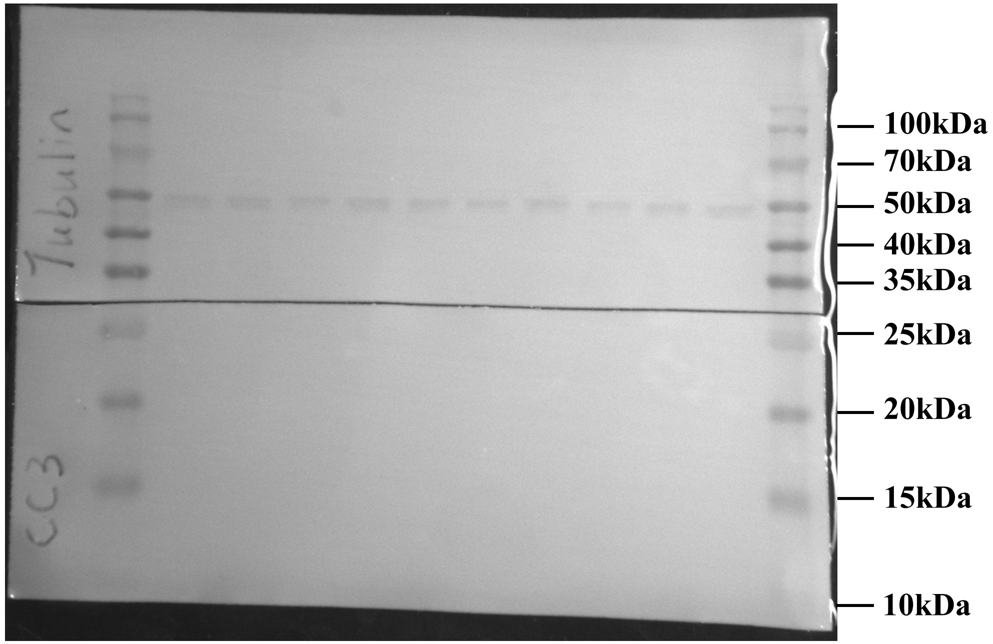

Full unedited gel for Figure S2F";

(2)

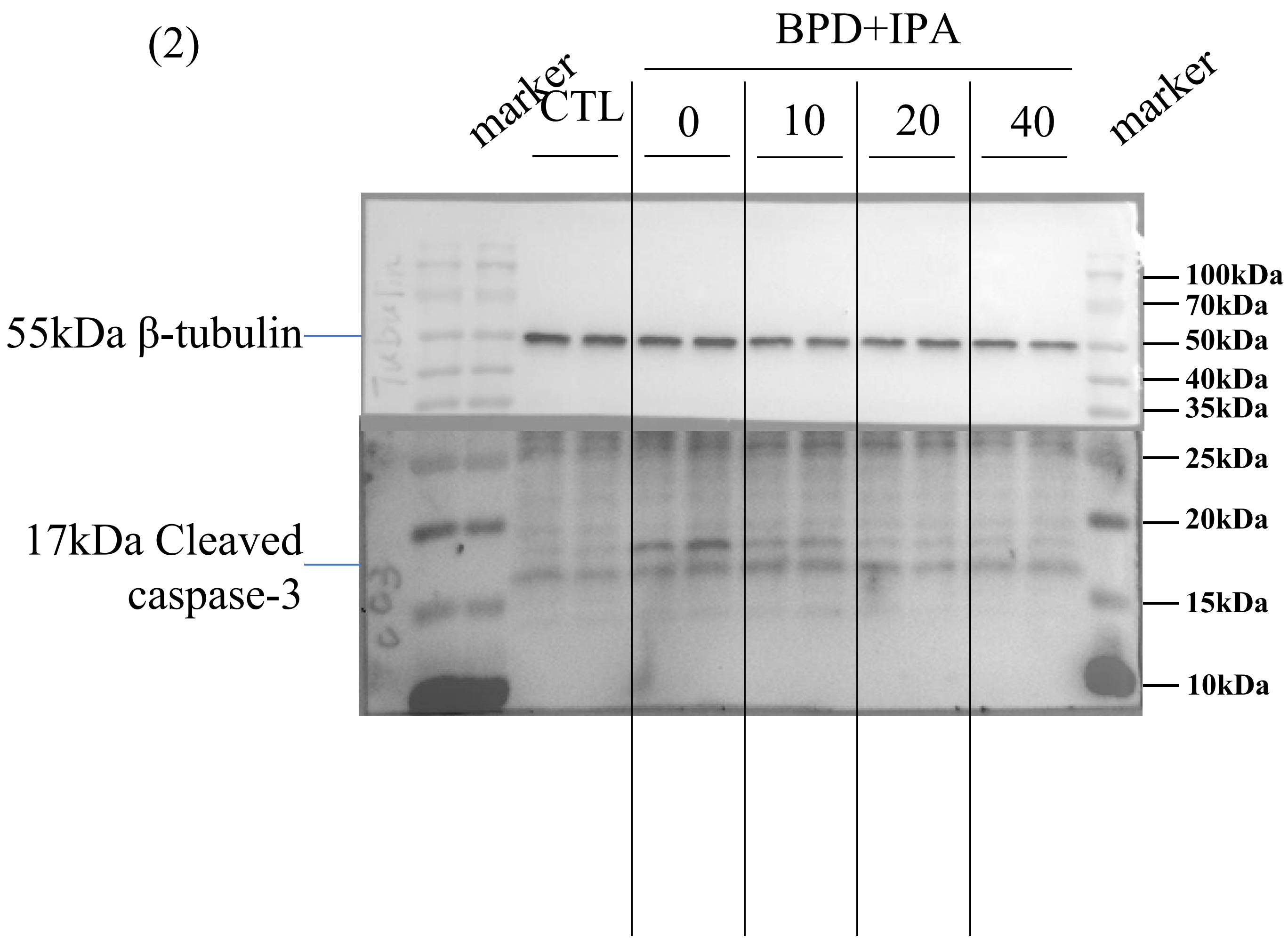

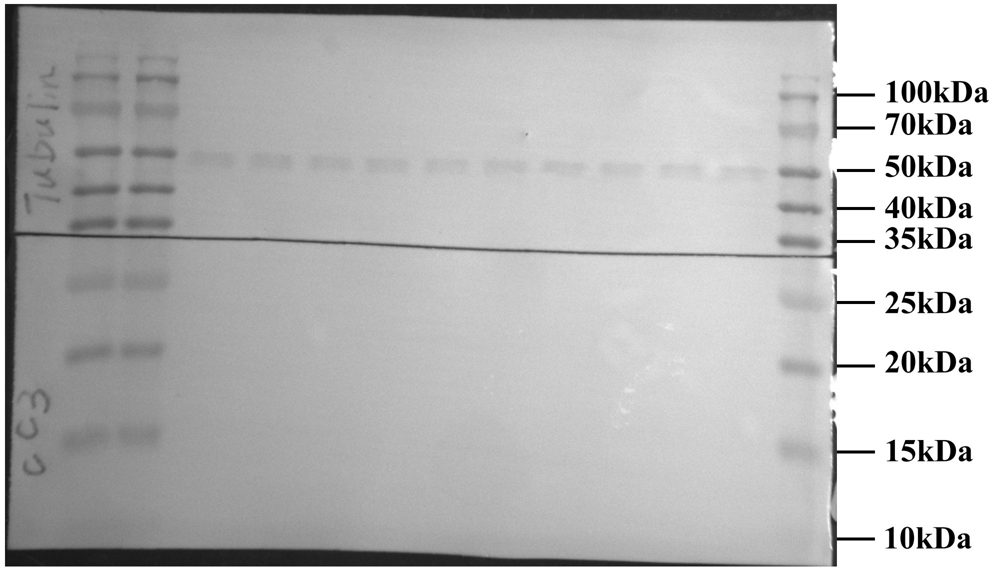

Full unedited gel for Figure S2F";

(3)

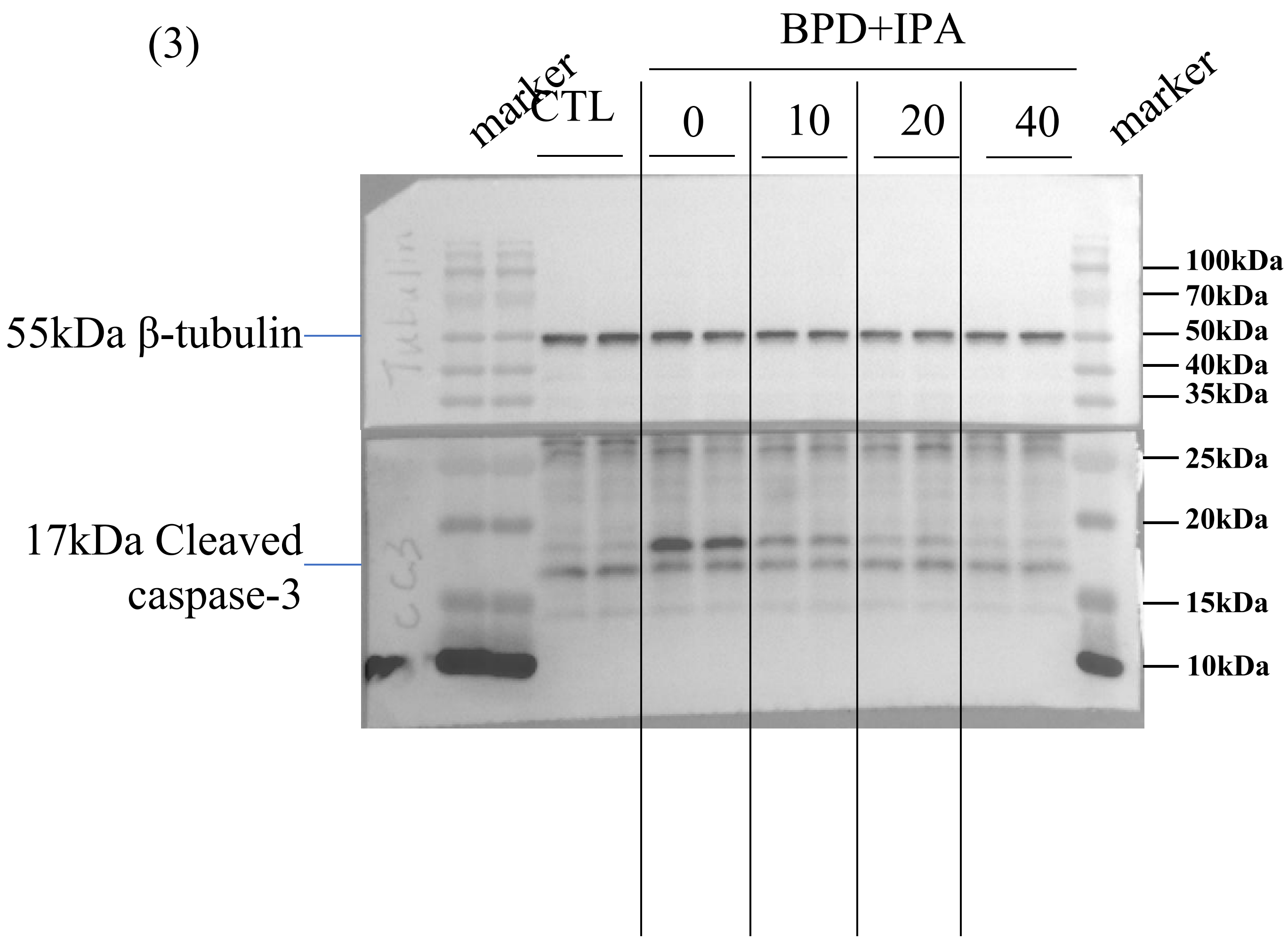

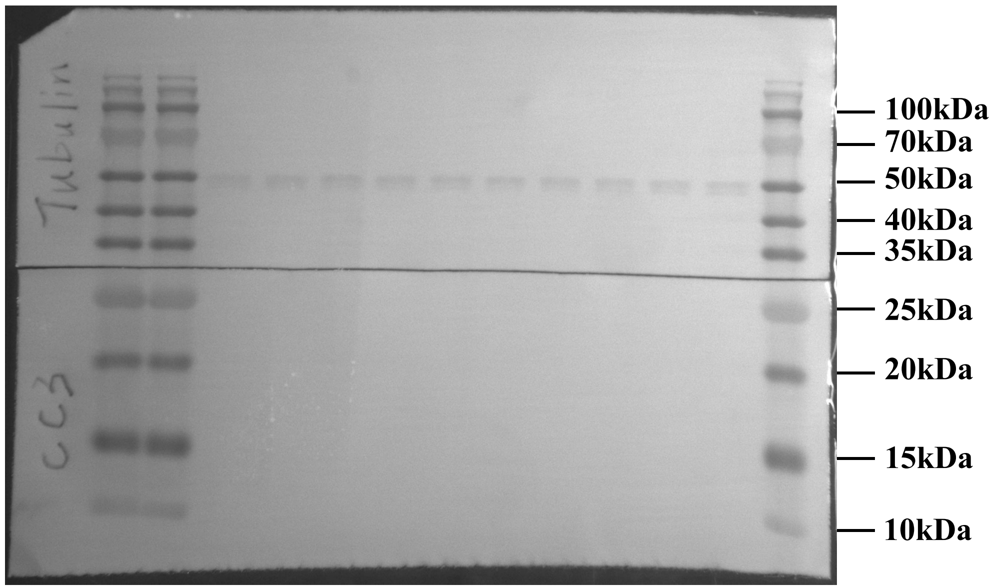

Full unedited gel for Figure S4";

Full unedited gel for Figure S4A";

(1)

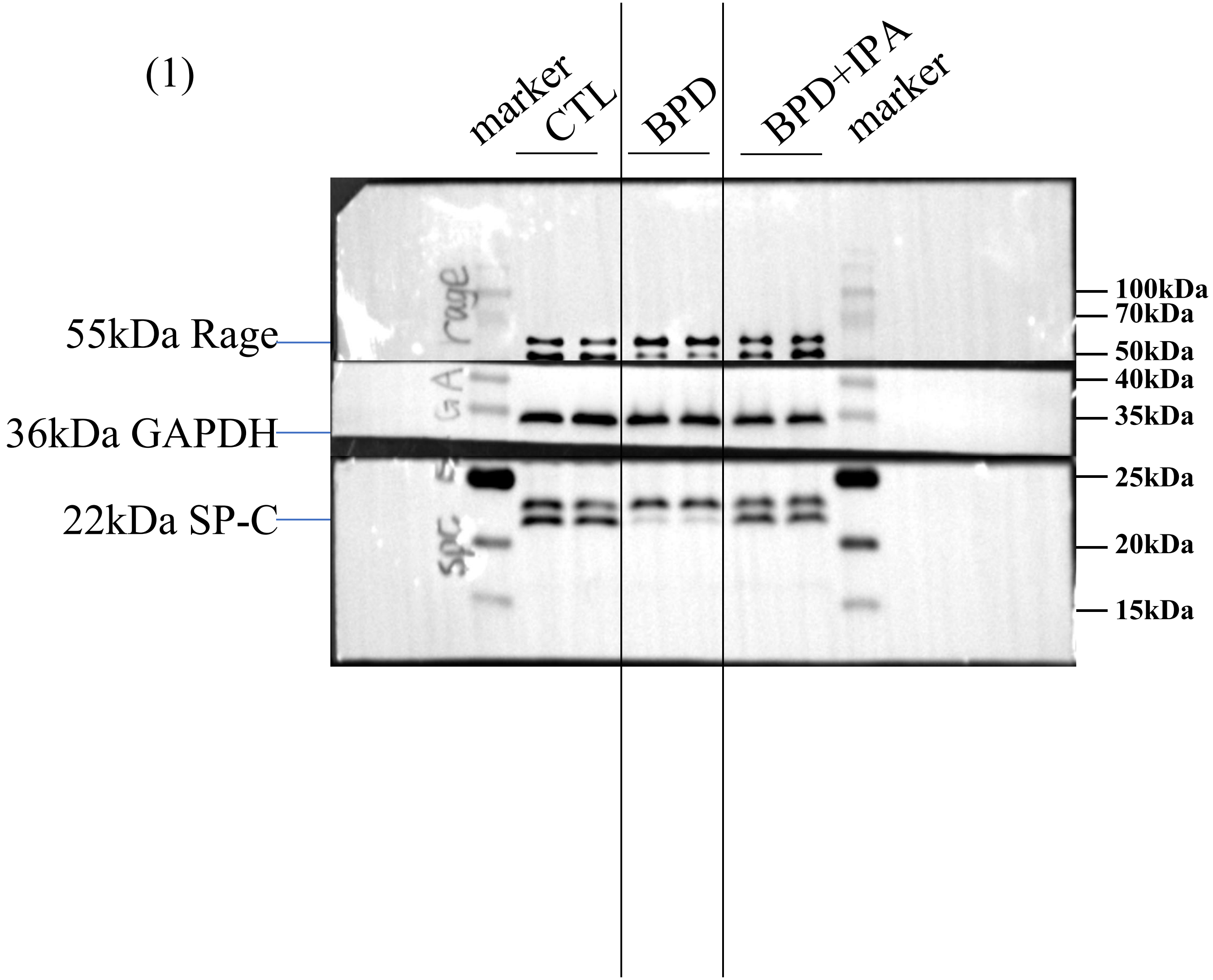

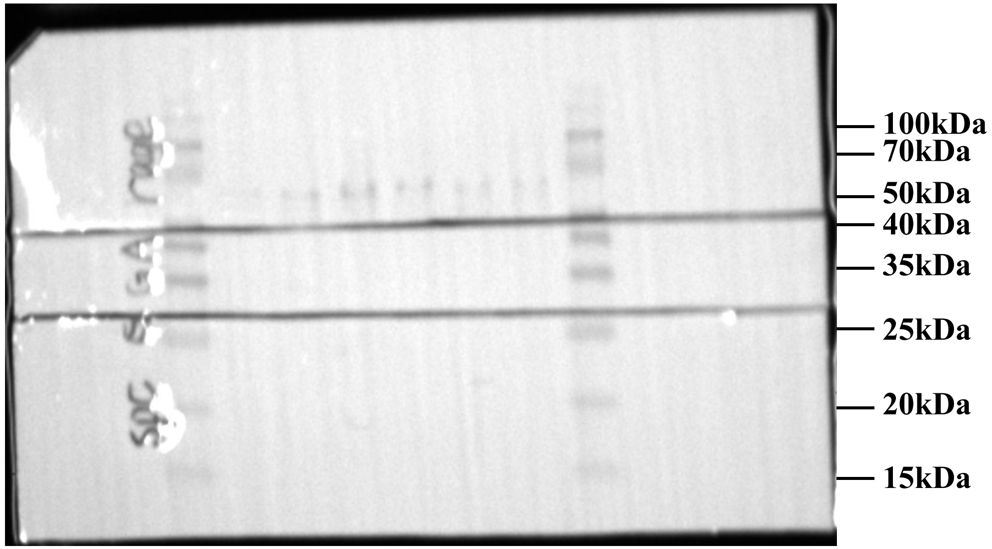

Full unedited gel for Figure S4A";

(2)

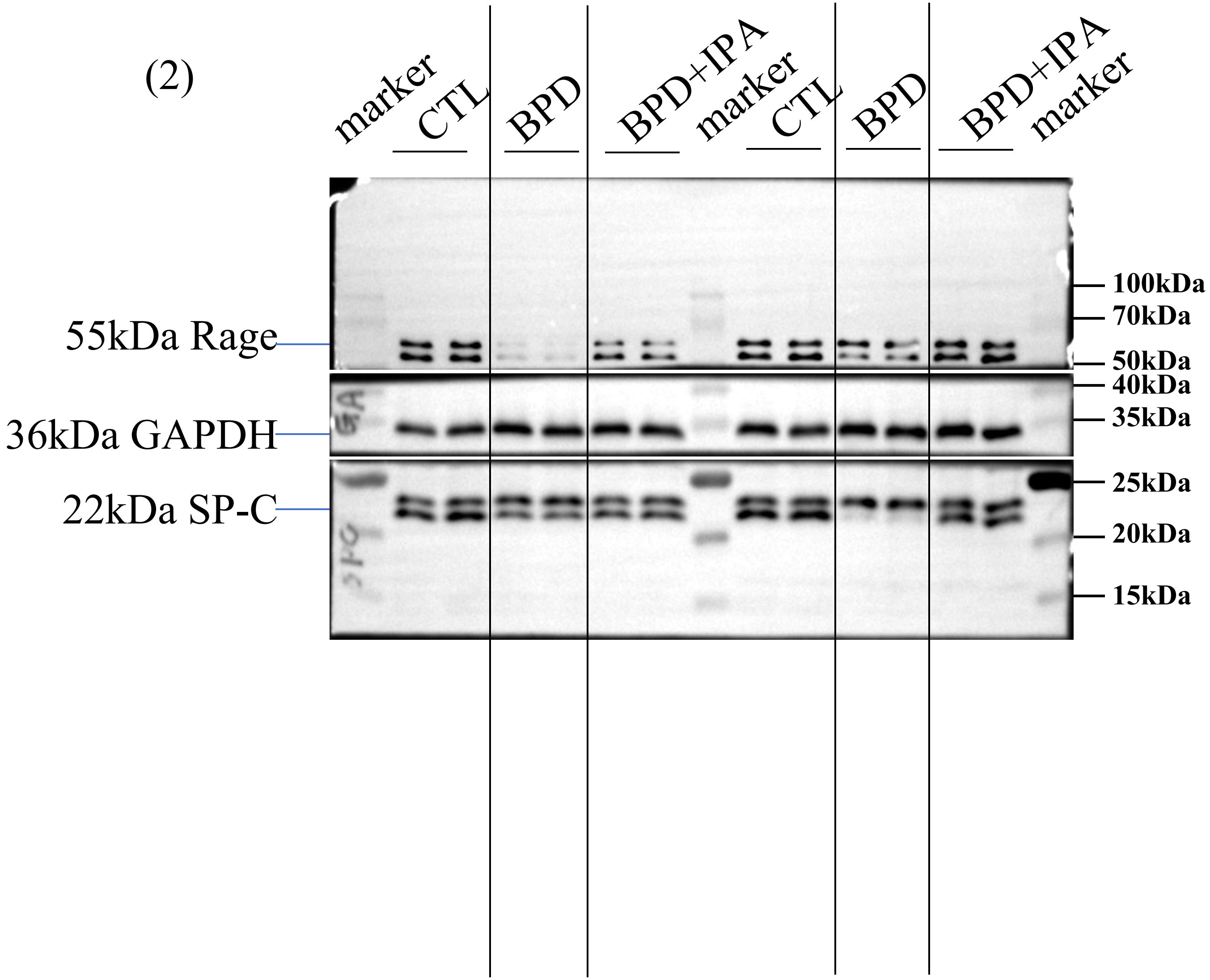

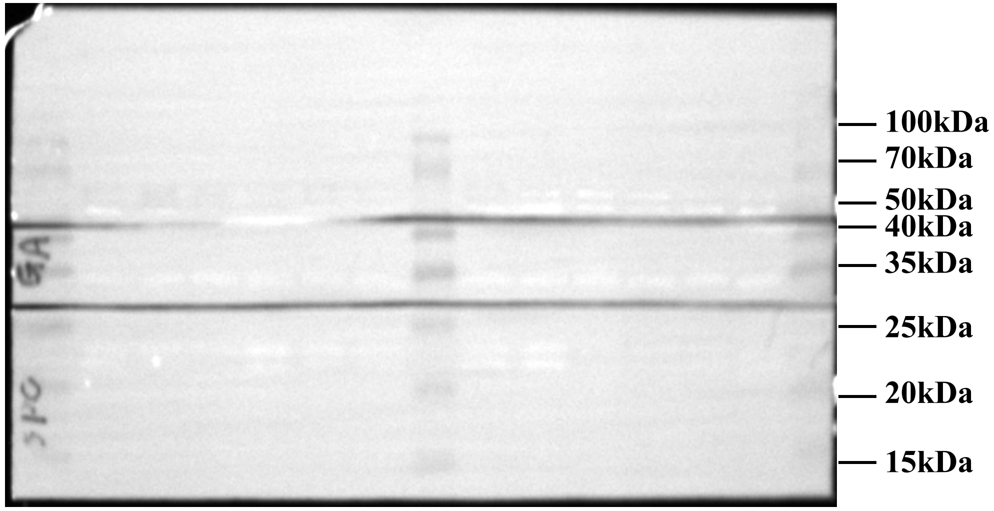

Full unedited gel for Figure S4H";

(1)

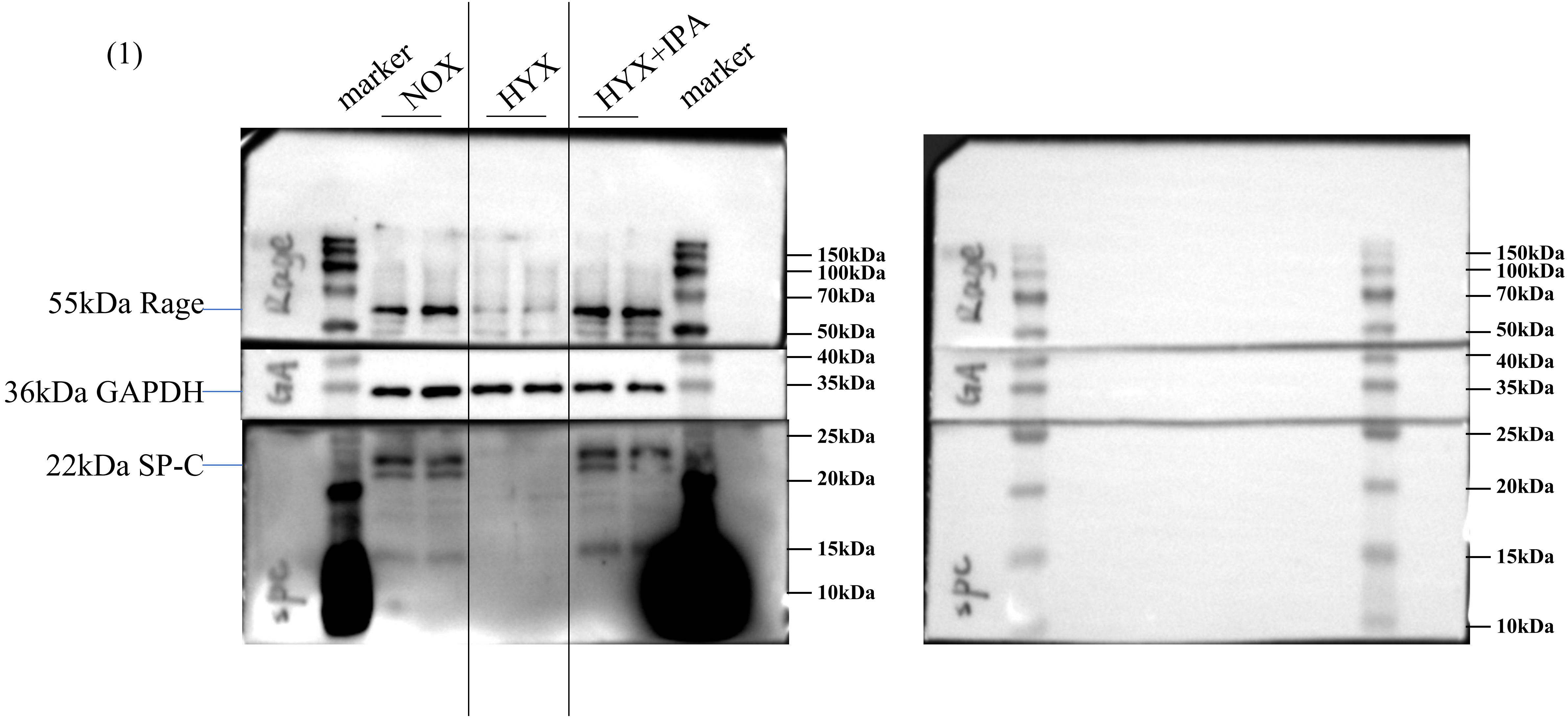

Full unedited gel for Figure S4H";

(2)

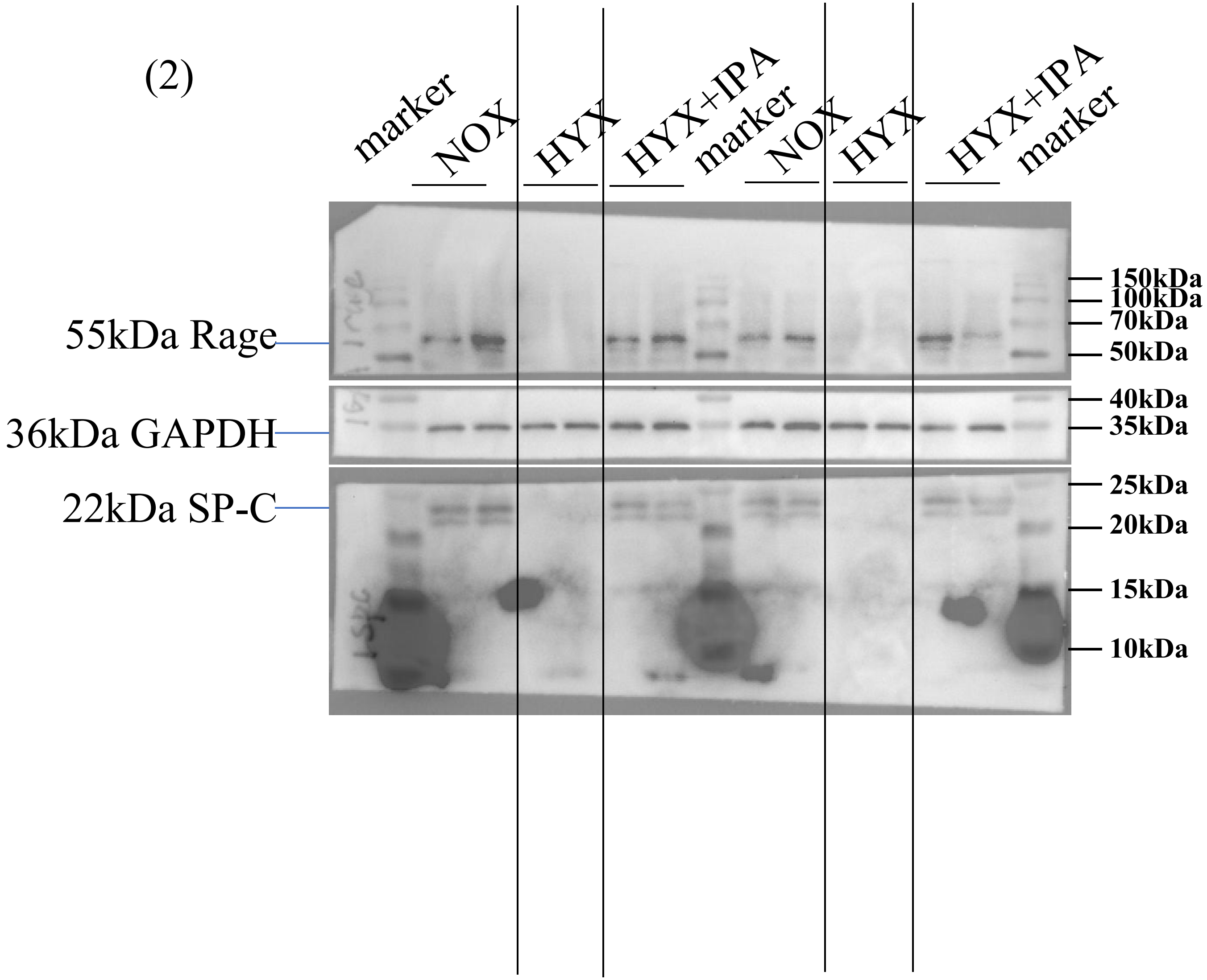

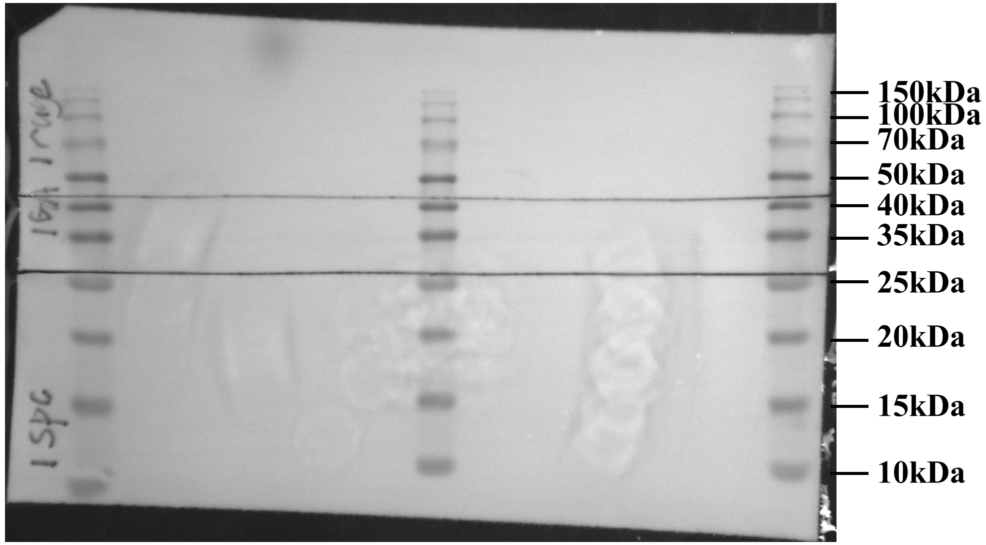

Full unedited gel for Figure S5";

Full unedited gel for Figure S5A";

(1)

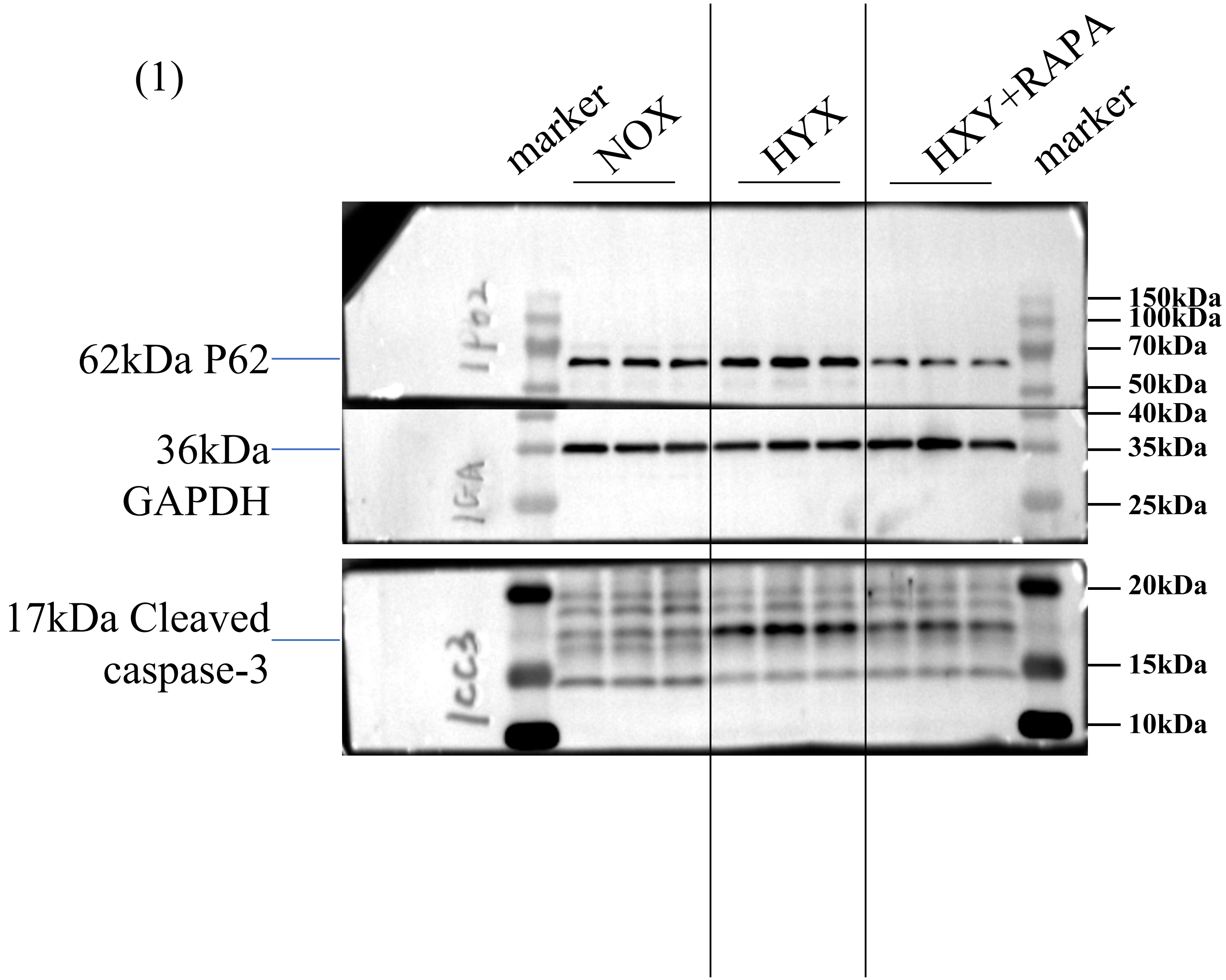

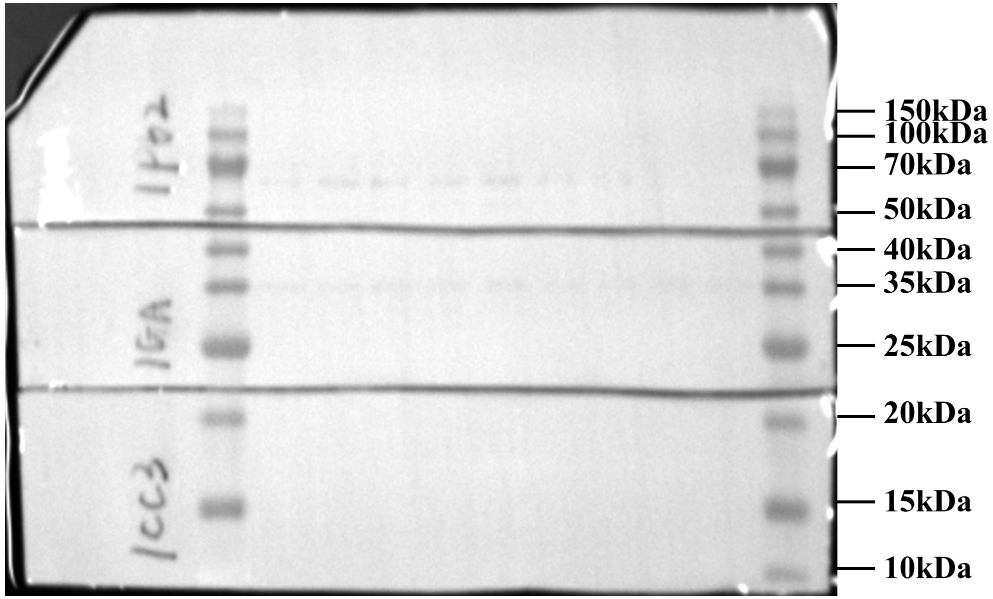

Full unedited gel for Figure S5A";

(2)

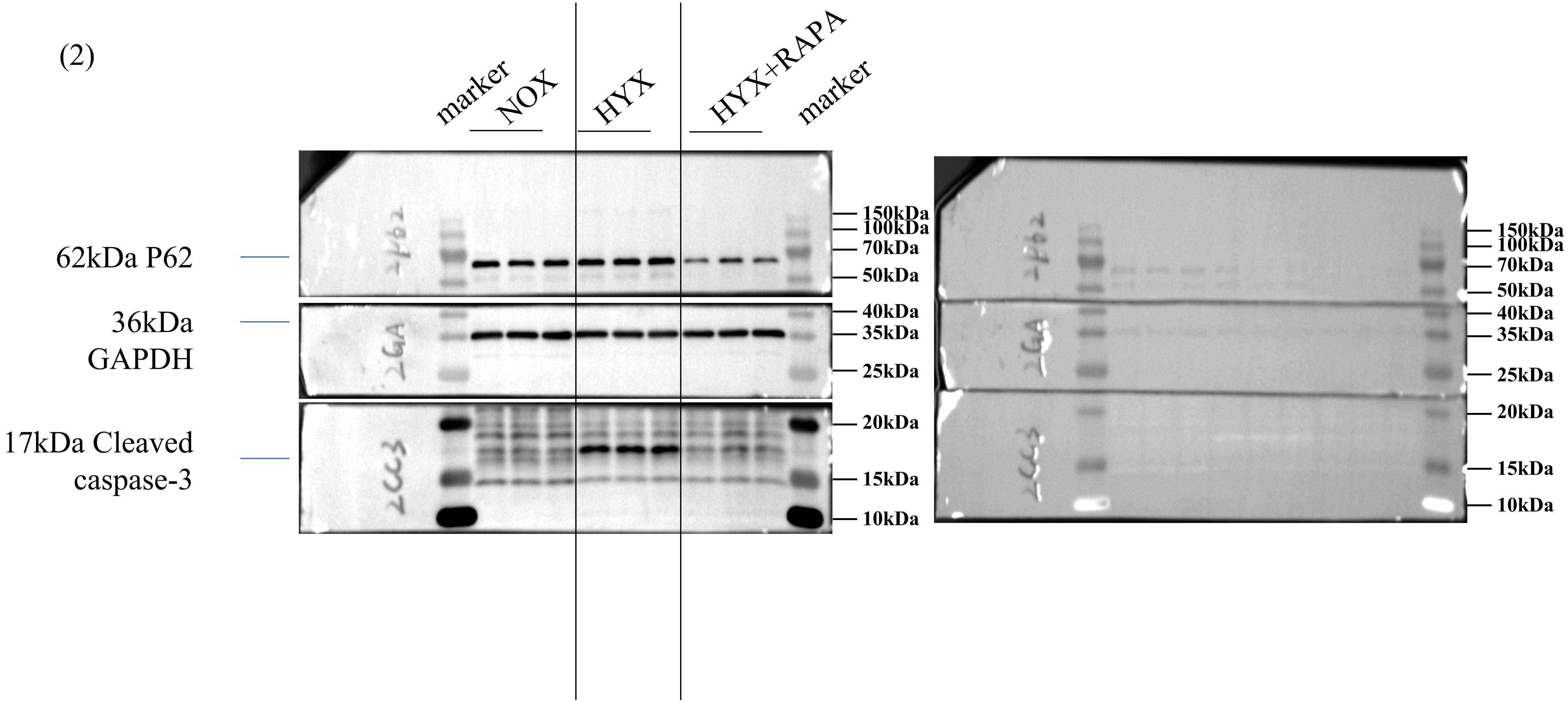

Full unedited gel for Figure S6";

Full unedited gel for Figure S6D";

(1)

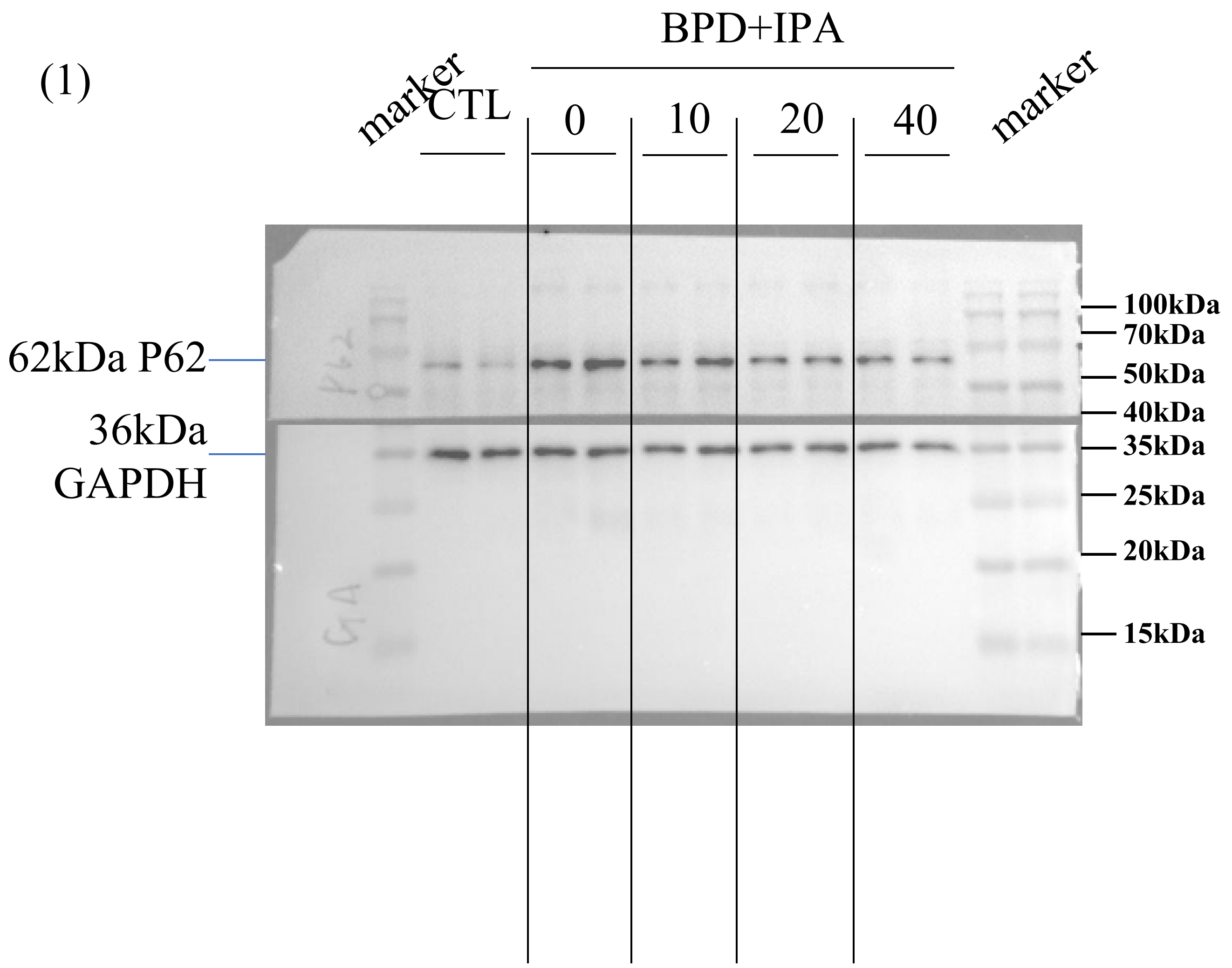

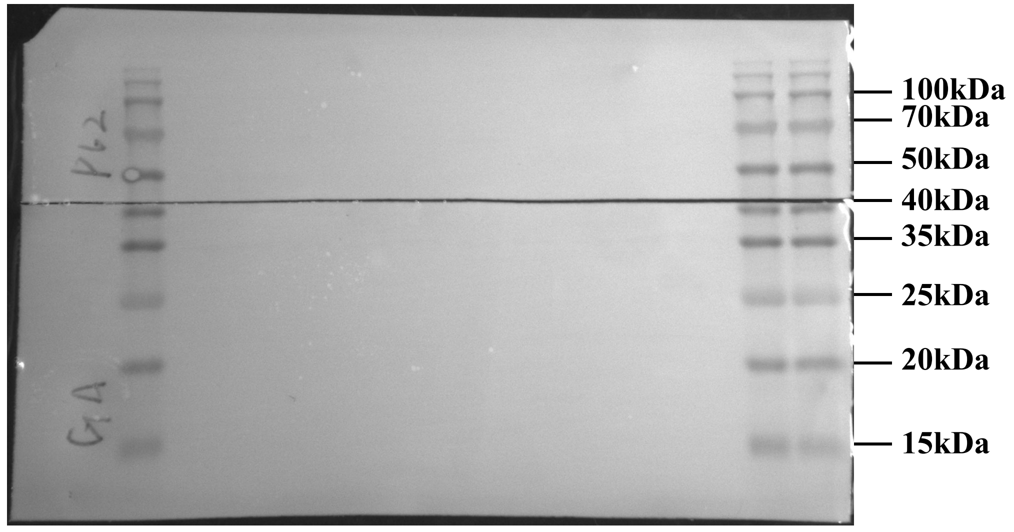

Full unedited gel for Figure S6D";

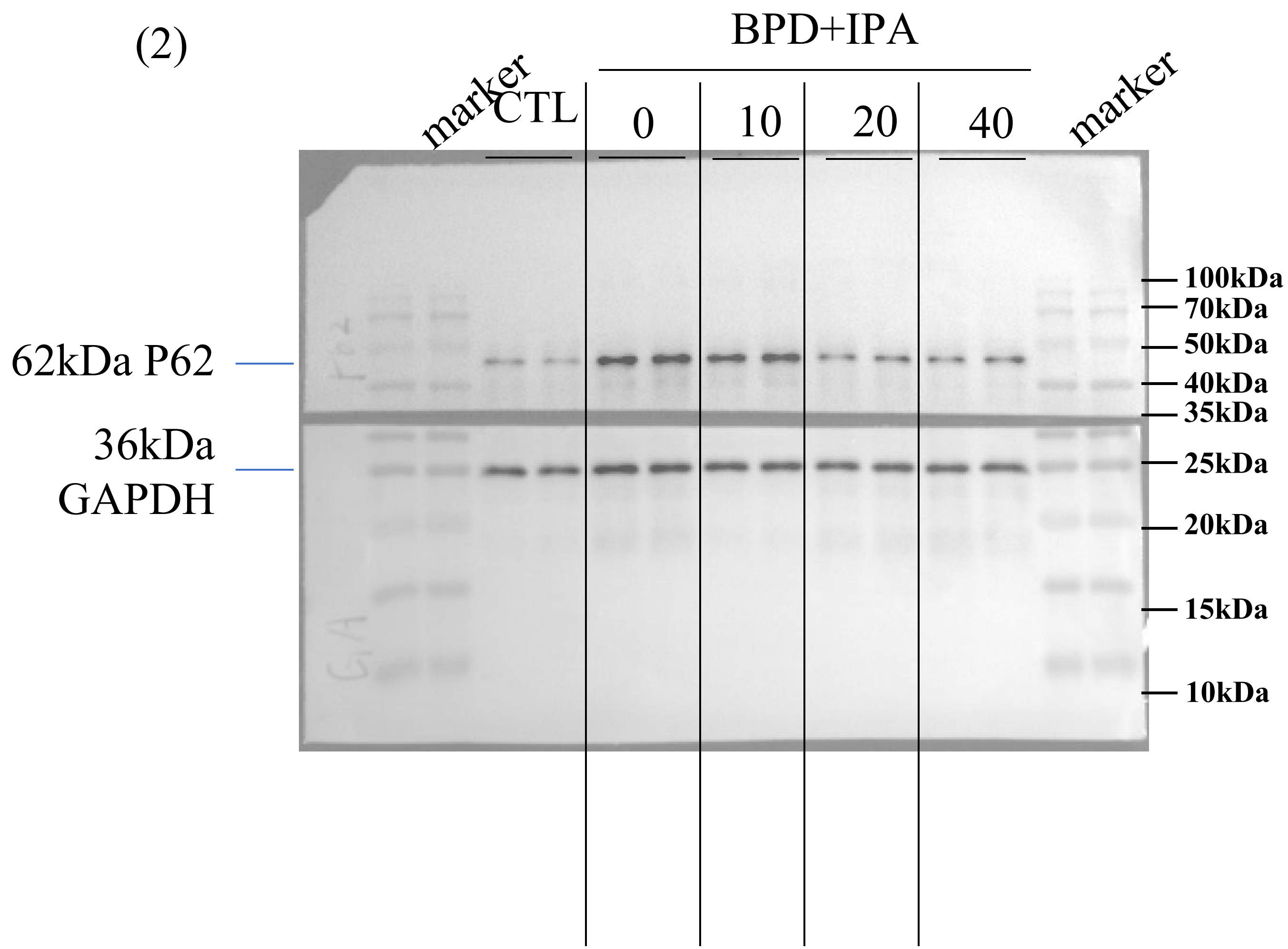

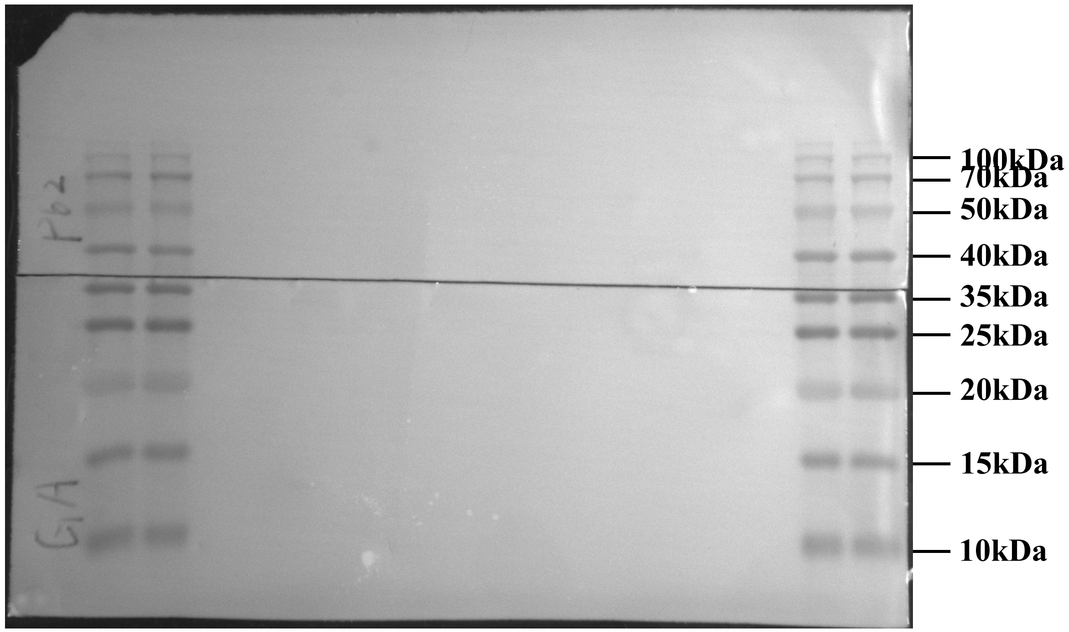

Full unedited gel for Figure S6D";

(3)

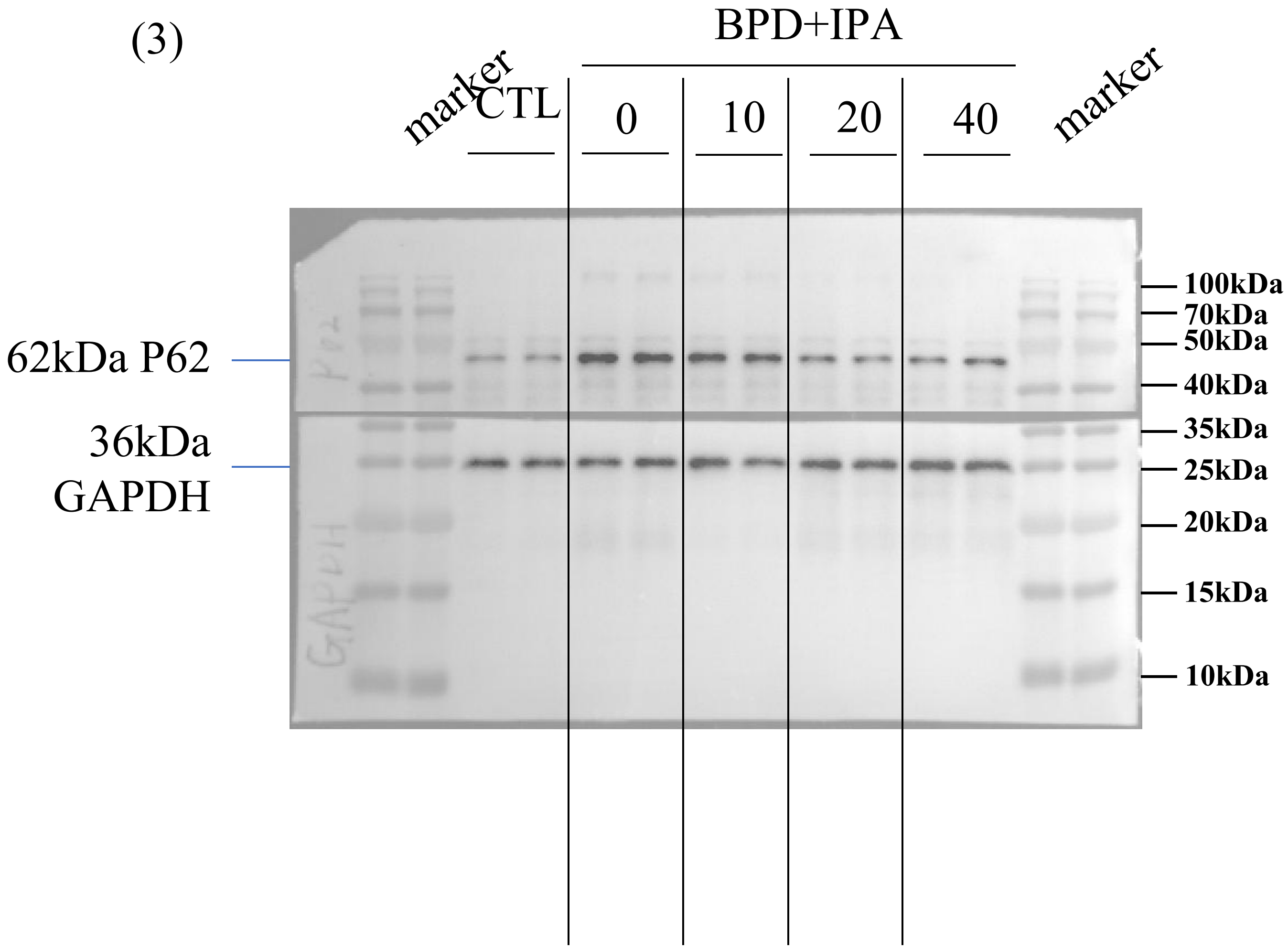

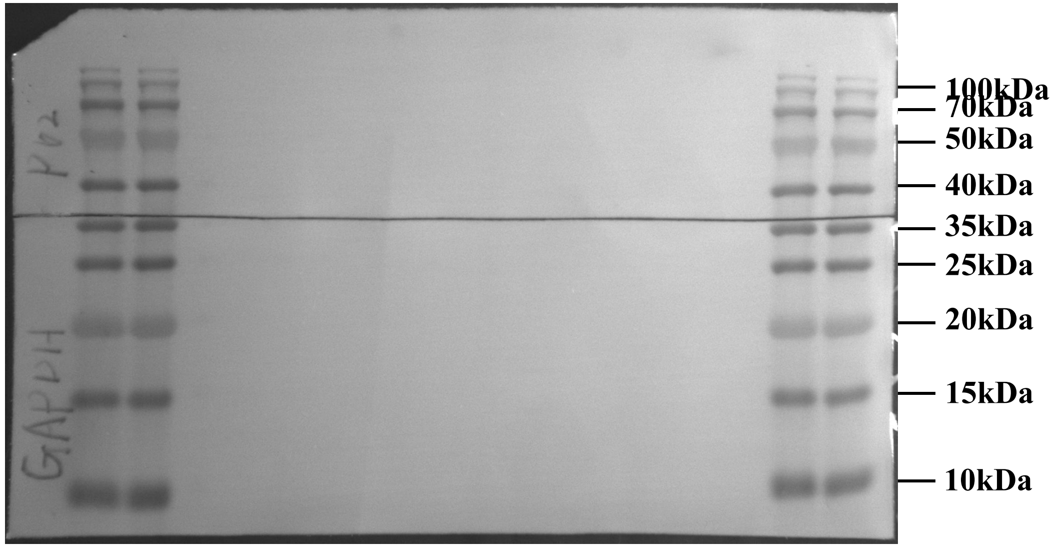

Full unedited gel for Figure S7";

Full unedited gel for Figure S7A";

(1)

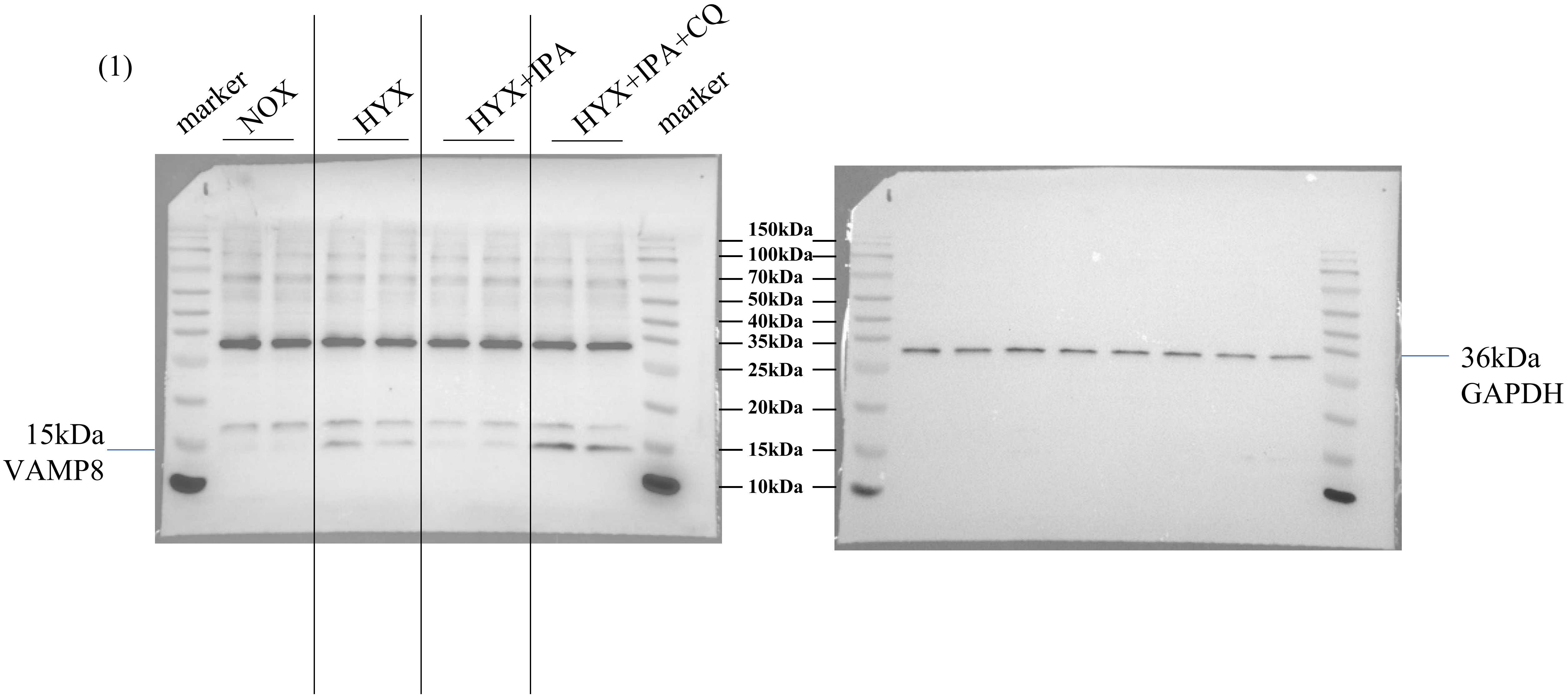

Supplement: Supplementary file 2 — Supporting File: advs74104‐supp‐0002‐DataFile. [file ADVS-13-e02610-s001.pdf]
